# p38γ and p38δ regulate postnatal cardiac metabolism through glycogen synthase 1

**DOI:** 10.1371/journal.pbio.3001447

**Published:** 2021-11-10

**Authors:** Ayelén M. Santamans, Valle Montalvo-Romeral, Alfonso Mora, Juan Antonio Lopez, Francisco González-Romero, Daniel Jimenez-Blasco, Elena Rodríguez, Aránzazu Pintor-Chocano, Cristina Casanueva-Benítez, Rebeca Acín-Pérez, Luis Leiva-Vega, Jordi Duran, Joan J. Guinovart, Jesús Jiménez-Borreguero, José Antonio Enríquez, María Villlalba-Orero, Juan P. Bolaños, Patricia Aspichueta, Jesús Vázquez, Bárbara González-Terán, Guadalupe Sabio

**Affiliations:** 1 Centro Nacional de Investigaciones Cardiovasculares (CNIC), Madrid, Spain; 2 CIBER de Enfermedades Cardiovasculares (CIBERCV), Madrid, Spain; 3 Department of Physiology, Faculty of Medicine and Nursing, University of the Basque Country UPV/EHU, Leioa, Spain; 4 Institute of Functional Biology and Genomics (IBFG), Universidad de Salamanca, CSIC, Salamanca, Spain; 5 Institute of Biomedical Research of Salamanca (IBSAL), Hospital Universitario de Salamanca, CSIC, Universidad de Salamanca, Salamanca, Spain; 6 Centro de Investigación Biomédica en Red de Fragilidad y Envejecimiento Saludable (CIBERFES), Madrid, Spain; 7 Institute for Research in Biomedicine (IRB Barcelona), The Barcelona Institute of Science and Technology, Barcelona, Spain; 8 Centro de Investigación Biomédica en Red de Diabetes y Enfermedades Metabólicas Asociadas (CIBERDEM), Madrid, Spain; 9 BioCruces Bizkaia Health Research Institute, Cruces University Hospital, Barakaldo, Spain; 10 Centro de Investigación Biomédica en Red de Enfermedades Hepáticas y Digestivas (CIBERehd), Madrid, Spain; Columbia University, UNITED STATES

## Abstract

During the first weeks of postnatal heart development, cardiomyocytes undergo a major adaptive metabolic shift from glycolytic energy production to fatty acid oxidation. This metabolic change is contemporaneous to the up-regulation and activation of the p38γ and p38δ stress-activated protein kinases in the heart. We demonstrate that p38γ/δ contribute to the early postnatal cardiac metabolic switch through inhibitory phosphorylation of glycogen synthase 1 (GYS1) and glycogen metabolism inactivation. Premature induction of p38γ/δ activation in cardiomyocytes of newborn mice results in an early GYS1 phosphorylation and inhibition of cardiac glycogen production, triggering an early metabolic shift that induces a deficit in cardiomyocyte fuel supply, leading to whole-body metabolic deregulation and maladaptive cardiac pathogenesis. Notably, the adverse effects of forced premature cardiac p38γ/δ activation in neonate mice are prevented by maternal diet supplementation of fatty acids during pregnancy and lactation. These results suggest that diet interventions have a potential for treating human cardiac genetic diseases that affect heart metabolism.

## Abstract 2

This study elucidates the role of the protein kinases p37γ and p38δ in regulating the metabolic switch that occurs in early postnatal development, revealing that they inhibit glycogen synthase 1 and glycogen metabolism. Deregulation of this mechanism results in cardiac defects and metabolic alterations which can be prevented by maternal fatty acid diet supplementation during pregnancy and lactation.

## Introduction

Adaptation of cardiomyocyte metabolism to heart growth is essential throughout life [1]. Production of ATP in fetal cardiomyocytes is highly dependent on glycolysis [2]. Soon after birth, high-energy demands and increasing levels of circulating non-esterified fatty acids (NEFAs; also called free fatty acids) trigger a shift in cardiomyocyte metabolism to a predominant reliance on fatty acid oxidation, which consumes more oxygen but also yields more ATP per molecule than glucose oxidation [3]. This critical metabolic switch from glycolytic to lipolytic metabolism is a key transition in cardiomyocyte maturation, and deregulation of this process can affect heart function. For example, deficient cardiomyocyte glycogen storage results in impaired cardiac function in neonatal animals [4], and disruption of respiratory chain function during cardiac development compromises the ability of cardiomyocytes to switch their metabolic profile and reorganize their mitochondria, thus impairing their contractile machinery [5]. Moreover, heart failure (HF) is characterized by a reversion to cardiomyocyte reliance on glycolysis [6], and it can be induced by a metabolic switch from fatty acid to glucose use in the adult heart [7]. Hence, a correct control of heart metabolism is essential for maintaining its functionality, and its deregulation can lead to heart disease.

Glycogen in heart is synthetized by muscle glycogen synthase 1 (GYS1) [8], which is a distinct isoform from the liver-specific glycogen synthase 2 (GYS2) [9]. GYS1 is regulated by phosphorylation at multiple sites by several kinases, which lead to its inactivation [10]. Cardiac glycogen is abundant during prenatal development but declines rapidly after birth, when cardiomyocytes become dependent on fatty acid metabolism [11,12], suggesting that glycogen may have a determinant role in heart development and may contribute to proper cardiomyocyte function [11,13]. Although glycogen storage during embryonic development has been studied, little is known about its function or its regulation during the early postnatal period. Stress-activated protein kinases (SAPKs) transform extracellular stimuli into a wide range of cellular processes and are key regulators of tissue homeostasis and metabolism [14]. The SAPKs p38γ and p38δ (herein referred to as p38γ/δ) promote cardiomyocyte hypertrophic growth, and their deficiency in mice lead to reduced heart size [15]. Undetectable in the fetal heart, the expression and activation of p38γ/δ increase in the first 2 weeks after birth [15]—a crucial period in heart development that requires a tight coordination between cardiac growth and the metabolic switch.

Here, we report that p38γ/δ control heart metabolism during the early postnatal period of cardiac development by regulating the activity of GYS1 and glycogen metabolism. We observed that the gradual up-regulation of p38γ/δ during postnatal development controls the metabolic switch by inducing GYS1 phosphorylation and inactivation, thereby reducing the cardiac glycogen storage. Indeed, forced overexpression of cardiomyocyte-specific, active p38γ/δ on postnatal day 1 (PD1) resulted in premature reduction of glycogen storage as well as a premature metabolic shift from glycolytic energy production to fatty acid oxidation that triggered maladaptive cardiac pathogenesis. Strikingly, cardiomyocyte metabolic changes led to altered whole-body homeostasis, including dyslipidemia, hyperglycemia, and insulin resistance. In contrast, mice with cardiomyocyte neonatal-specific deletion of p38γ/δ showed increased glycogen storage. Depletion of glycogen storage soon after birth by conditional deletion of *Gys1* in cardiomyocytes led to perinatal cardiac dysfunction and metabolic changes similar to the ones observed in the p38γ/δ–overexpressing model. Thus, our study provides strong evidence that cardiomyocyte p38γ/δ expression and activation after birth are important regulators of heart glycogen metabolism and are responsible for driving the cardiomyocyte postnatal metabolic switch. In addition, we demonstrate that premature induction of this metabolic switch resulted in cardiac dysfunction and alteration of whole-body metabolism, which could be prevented by maternal fatty acid diet supplementation during pregnancy and lactation.

## Results

### Premature neonatal activation of p38γ/δ signaling in cardiomyocytes causes cardiac dysfunction

**Fig 1:**
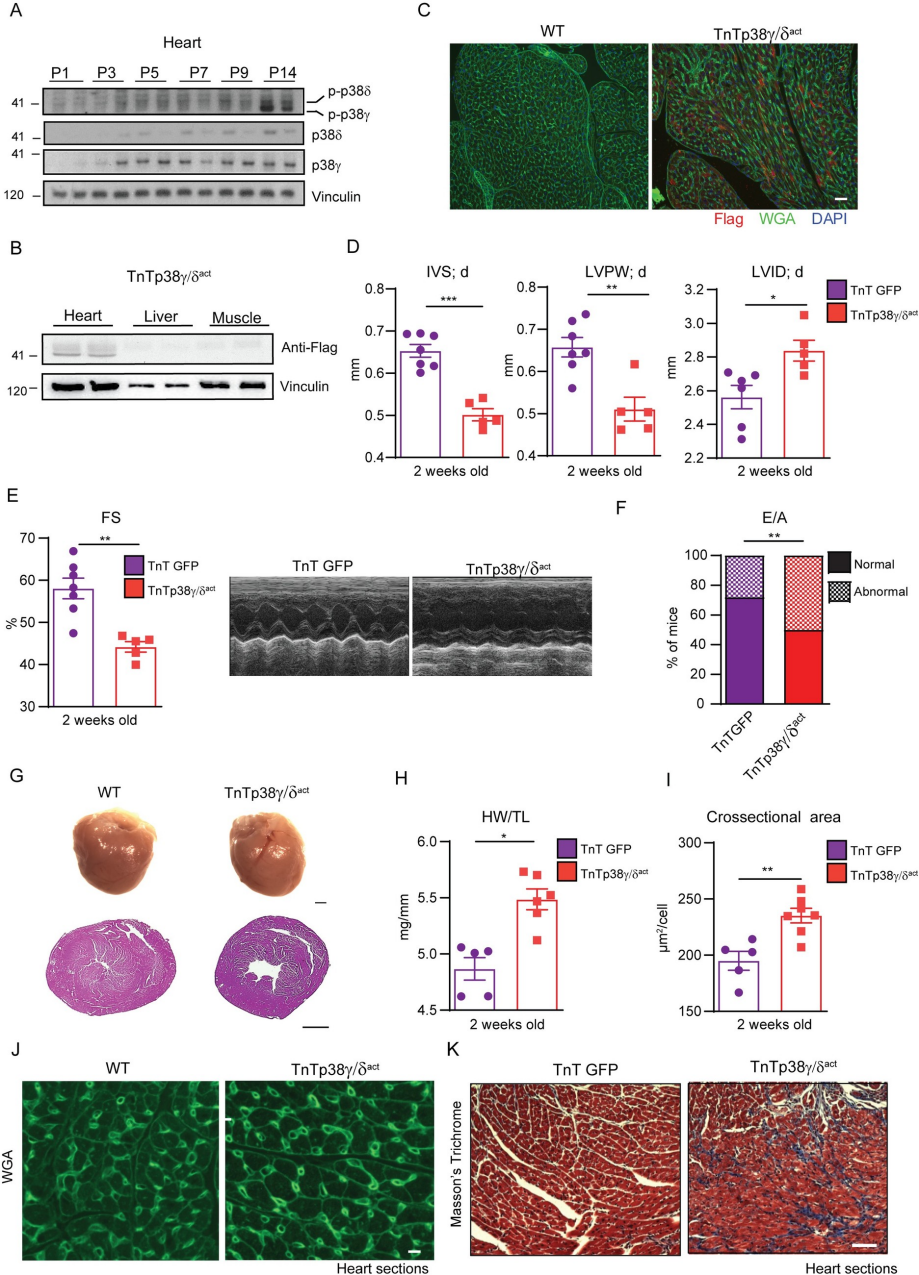
Cardiomyocyte-specific postnatal overexpression of p38γ/δ^act^ causes cardiac eccentric hypertrophy and heart dysfunction. **(A)** Immunoblot analysis of endogenous p38γ/δ in heart extracts from WT (non-injected) mice at PD 1, 3, 5, 7, 9, and 14. (B) Immunoblot using anti-FLAG antibody showing p38γ/δ^act^ specific cardiac overexpression at PD14 after AAV-cTnT-p38γ/δ^act^ injection (at PD1), in heart, liver, and muscle extracts. (C) Immunofluorescence of FLAG-p38γ/δ^act^ (red), WGA (green), and DAPI (blue) on heart sections from WT or TnTp38γ/δ^act^ mice. Scale bar: 200 μm. (D–K) Analyses of hearts at PD14 of AAV-cTnT-GFP-Luc (TnTGFP; control mice) or TnTp38γ/δ^act^ mice (with AAV injection at PD1), showing the following: (D) echocardiography measurements. (E) FS and M-mode short-axis echocardiography traces; (F) percentage of mice at PD14 with normal or abnormal mitral valve flow (E/A) as an indicator of diastolic dysfunction; (G) images of whole heart (scale bar: 1 mm) and H&E staining of transverse heart sections (scale bar: 1 mm); (H) HWTL ratio; (I, J) chart of FITC-WGA staining of hearts (green), with cardiomyocyte cross-sectional area quantification (scale bar: 50 μm); (K) Masson’s trichrome staining images from heart sections (the respective fibrosis quantification is shown in S1B Fig). Scale bar: 200 μm. Data are mean ± SEM (*n =* 5–8). *p < 0.05; **p < 0.01; ***p < 0.001 by two-tailed Student *t* test. Raw data are given in S14 Fig. AAV, adeno-associated virus; FS, fractional shortening; HWTL, heart weight-to-tibia length; H&E, hematoxylin and eosin; IVS; d, interventricular septum thickness in diastole; LVID; d, left ventricular internal diameter in diastole; LVPW; d, left ventricle posterior wall thickness in diastole; PD, postnatal day; WT, wild-type.

Cardiac expression of p38γ/δ and their subsequent phosphorylation, which triggers their activation, is very low at birth and increase postnatally in the first 2 weeks [15] (Fig 1A). This period coincides with the metabolic adaptations of the fetus/newborn with respect to nutritional transition and to the cardiomyocyte hypertrophic growth, which requires a higher energy demand [16]. To understand the role of p38γ/δ in early postnatal cardiac development, we injected mice with serotype 9 adeno-associated virus (AAV) that overexpressed the constitutively active forms of p38γ/δ (p38γ/δ^act^) under the control of the cardiac troponin T promoter (TnTp38γ/δ^act^). Cardiomyocyte-specific overexpression was verified by immunoblot analyses of heart, liver, and muscle lysates, as well as by immunofluorescence using anti-FLAG antibody in TnTp38γ/δ^act^ cardiac tissue. No signal was detected in liver or muscle from TnTp38γ/δ^act^ mice or in heart from control mice not infected with virus (Fig 1B and 1C, S1 Fig).

To study the effects of premature p38γ/δ expression and activation in cardiomyocytes, we compared mice injected at PD1 with either TnTp38γ/δ^act^ or a control virus encoding luciferase under the cTNT promoter (AAV-cTnT-GFP-Luc, referred hereafter as TnTGFP). Echocardiographic analyses of PD14 mouse hearts demonstrated eccentric hypertrophy in TnTp38γ/δ^act^ mice as compared to control (TnTGFP) mice, with: (i) thinning of the diastolic interventricular septum (IVS; d); (ii) thinning of the diastolic left ventricular posterior wall (LVPW; d); and (iii) an increased left ventricular diameter (LVID; d) (Fig 1D). This cardiac enlargement compromised systolic and diastolic function, evidenced by a decreased fractional shortening (FS) and an increased percentage of mice with abnormal E/A ratios (Fig 1E and 1F). As no differences were observed between males and females, data were analyzed as a whole (S2 Fig). Gross anatomic and histologic analyses confirmed these noninvasive findings: TnTp38γ/δ^act^ hearts were larger than control hearts when normalized to tibia length (using heart weight-to-tibia length (HW/TL)) (Fig 1G and 1H, S3A Fig), and the larger size correlated with an increased cardiomyocyte cross-sectional area (Fig 1I and 1J). Further histological cardiac examination revealed fibrosis and altered structure, in line with the systolic and diastolic dysfunction seen by echocardiography (Fig 1K, S3B and S3C Fig). TnTp38γ/δ^act^ were sensitized to myocardial infarction (MI) and showed greater cardiac dilation and decreased heart function at 4 weeks after MI, as compared to control mice (S3D Fig). Altogether, premature p38γ/δ activation in cardiomyocytes of newborn mice led to early eccentric cardiac hypertrophy with decreased systolic and diastolic function accompanied by cardiac fibrosis, all hallmarks of dilated cardiomyopathy. Further, this phenotype gave a predisposition for a worse recovery after a cardiac insult.

### Premature cardiomyocyte p38γ/δ activation decreases cardiac glycogen storage and modulates the postnatal cardiac metabolic switch

**Fig 2:**
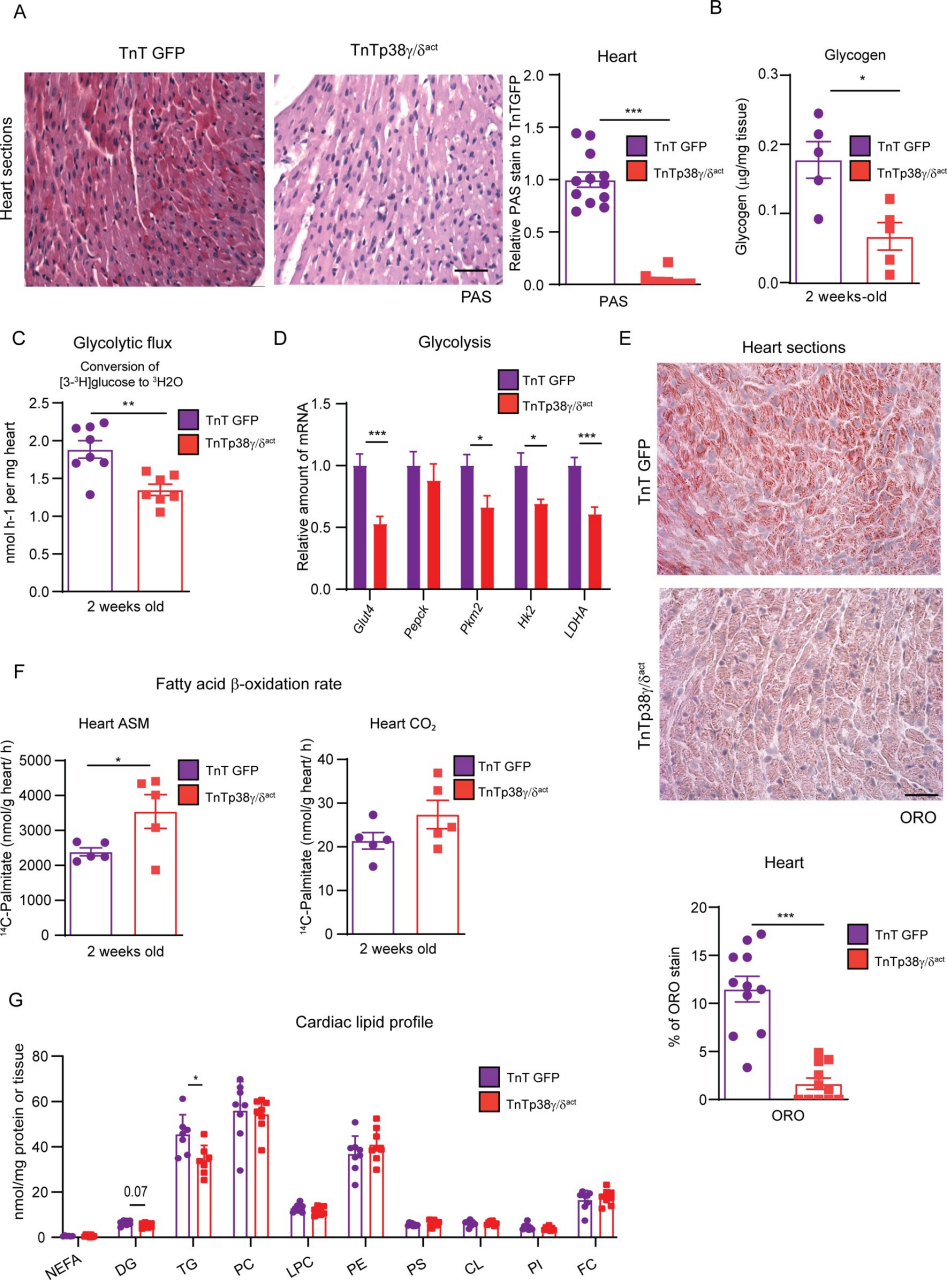
Cardiac-specific p38γ/δ^act^ overexpression decreases cardiac glycogen storage and lipid accumulation. Mice were IV injected at PD1 with AAV-cTnT-GFP-Luc (TnTGFP) or AAV-cTnT-p38γ/δ_act_ (TnTp38γ/δ^act^) and killed at PD14. **(A)** PAS staining in heart sections of TnTp38γ/δ^act^ or TnTGFP mice (left) and quantification (right). Scale bar: 200 μm**. (B)** Cardiac glycogen quantification. **(C)** Conversion of [3-^3^H] glucose into ^3^H_2_O, reflecting the glycolytic rate in hearts from TnTGFP and TnTp38γ/δ^act^ mice. **(D)** qRT-PCR analysis of glycolytic enzymes expression in cardiac tissue. **(E)** ORO staining in heart sections, showing representative images. Quantification chart below. Scale bar: 50 μm**. (F)** Cardiac fatty acid oxidation rate of ^14^C-palmitate determined by the production of CO_2_ and ASMs. **(G)** Cardiac lipid profile. All lipid amounts were normalized by mg of protein except for NEFAs, which were relativized by mg of tissue. Data are mean ± SEM. (*n =* 5–12). *p < 0.05; **p < 0.01; ***p < 0.001 by Student *t* test. Raw data are given in S14 Fig. ASM, acid-soluble metabolite; CL, cardiolipin; DG, diglycerides; FC, free cholesterol; LPC, lysophosphatidylcholine; NEFA, non-esterified fatty acid; ORO, oil red O; PAS, periodic acid–Schiff; PC, phosphatidylcholine; PE, phosphatidylethanolamine; PI, phosphatidylinositol; PS, phosphatidylserine; qRT-PCR, real-time quantitative PCR; TG, triglyceride.

As neonatal hearts rely on glycogen metabolism, we first studied whether premature activation of p38γ/δ in cardiomyocytes affected cardiac metabolism by altering glycogen deposition. Periodic acid–Schiff (PAS) staining and biochemical quantification showed that hearts from TnTp38γ/δ^act^ mice had reduced glycogen as compared to TnTGFP mice (Fig 2A and 2B). Using these mice, we next evaluated the effects of the reduced glycogen storage. We observed that TnTp38γ/δ^act^ hearts showed a decreased glycolytic flux, based on the conversion of [3-^3^H] glucose into ^3^H_2_O that takes place during triose-phosphate isomerase production (Fig 2C). Notably, the expression levels of multiple enzymes involved in glycolysis were significantly reduced in hearts from TnTp38γ/δ^act^ mice as compared to those from TnTGFP controls (Fig 2D). In agreement with a premature metabolic switch, TnTp38γ/δ^act^ hearts presented reduced heart lipid deposition measured by oil red O (ORO) staining correlating with an increased cardiac fatty acid oxidation at PD14 as compared to control hearts. This was indicated by the measurement of ^14^C-palmitate oxidation through the quantification of the resulting levels of acid-soluble metabolites (ASMs), which were mainly comprised of acetyl-CoA from incomplete β-oxidation (Fig 2E and 2F). A similar tendency was observed for CO_2_ levels resulting from radiolabeled palmitate complete oxidation through the TCA cycle (Fig 2F). Quantification of cardiac lipids indicated that TnTp38γ/δ^act^ hearts have reduced triglycerides (to a large degree) and diglycerides (to a lesser degree) (Fig 2G). In fact, these hearts presented slightly increased AMPK and acetyl-CoA carboxylase (ACC) protein levels, suggesting a deficit in cardiac energy (S4A Fig). However, we did not observe any alterations in the relative abundance of mitochondrial complexes (measured as total number or activity) (S4B–S4D Fig) or in the expression of genes involved in lipid metabolism (S4E Fig), suggesting that the metabolic differences did not stem from dysfunctional mitochondria but rather from differences in substrate availability. Overall, these results strongly suggest that early p38γ/δ overexpression produced a change in cardiac fuel, triggering an early metabolic shift from glycolytic energy production to fatty acid oxidation.

### Premature activation of cardiomyocyte p38γ/δ signaling alters whole-body metabolism

**Fig 3:**
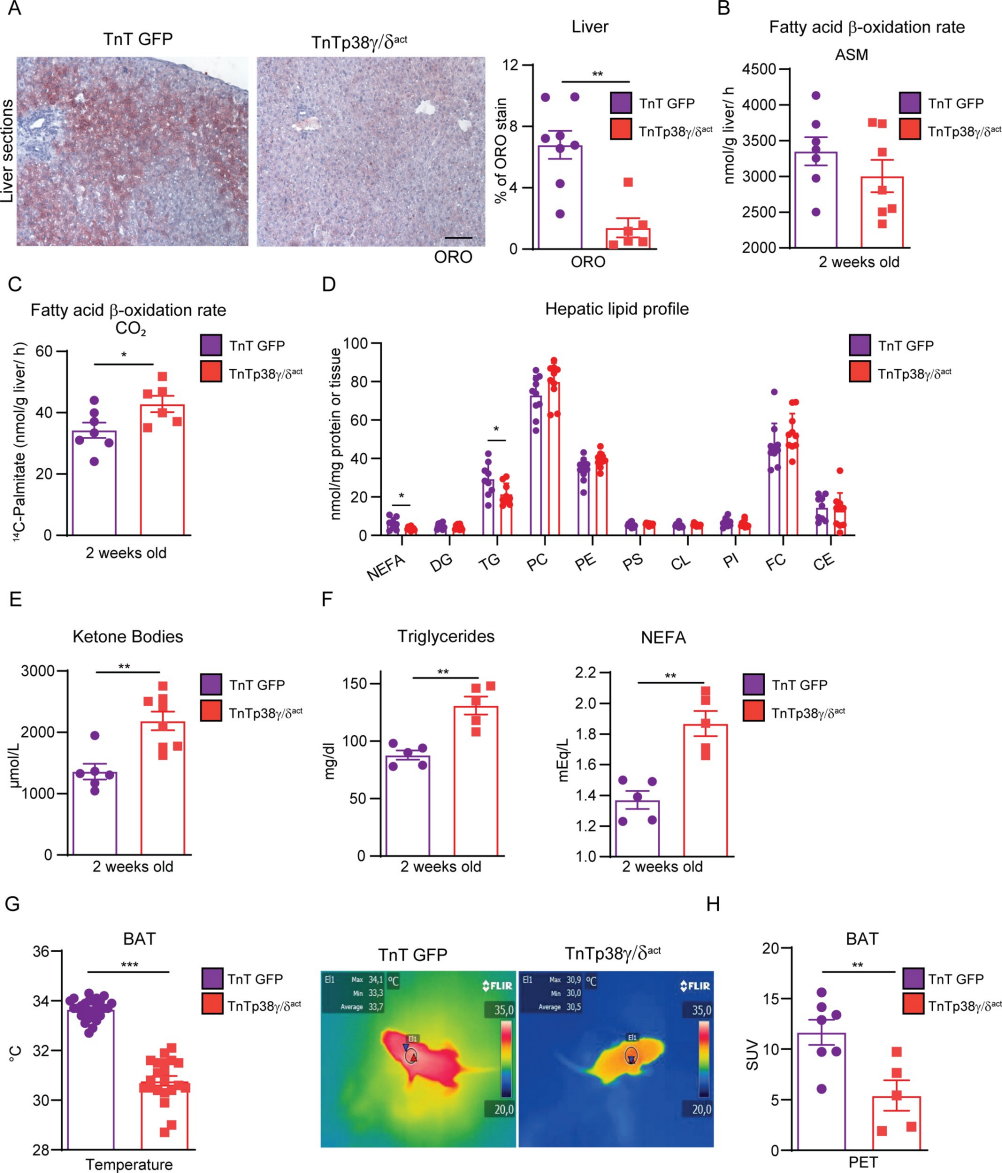
Cardiac-specific p38γ/δ^act^ overexpression has whole-body metabolic consequences. Mice were IV injected at PD1 with AAV-cTnT-GFP-Luc (TnTGFP) or AAV-cTnT-p38γ/δ_act_ (TnTp38γ/δ^act^); after metabolic tests were performed at PD14, mice were killed. **(A)** ORO staining (left) and quantification (right) of liver sections. Scale bar: 50 μm. **(B, C)** Hepatic fatty acid oxidation rate of ^14^C-palmitate, determined by the production of ASMs and CO_2_. **(D)** Hepatic lipid profile. All lipid amounts were normalized by mg of protein except for NEFAs, which were relativized to mg of tissue. **(E)** Plasma ketone bodies. **(F)** Plasma TG and NEFA. **(G)** Mice BAT temperatures, with its representative thermographic images. **(H)** BAT glucose uptake measured by PET-CT. Regions of interest were delimited to the BAT area to obtain the mean SUV. Data are mean ± SEM (*n =* 7–10). *p < 0.05, **p < 0.01; ***p < 0.001, by Student *t* test. Raw data are given in S14 Fig. ASM, acid-soluble metabolite; BAT, brown adipose tissue; CE, cholesteryl ester; CL, cardiolipin; DG, diglyceride; FC, free cholesterol; LPC, lysophosphatidylcholine; NEFA, non-esterified fatty acid; ORO, oil red O; PC, phosphatidylcholine; PE, phosphatidylethanolamine; PET-CT, positron emission tomography–computed tomography; PI, phosphatidylinositol; PS, phosphatidylserine; SUV, standard uptake value; TG, triglyceride.

We next studied liver metabolism to determine whether a switch in cardiac fuel use has whole-body metabolic consequences. We found that livers from TnTp38γ/δ^act^ mice at PD14 had a reduced lipid deposition, as measured by ORO staining (Fig 3A). Concordantly, livers from TnTp38γ/δ^act^ mice showed increased fatty acid oxidation through the TCA cycle as compared to controls, as indicated by the higher CO_2_ levels generated upon incubation of liver homogenates with radiolabeled ^14^C-palmitate. However, no significant differences were observed in ASM levels generated from incomplete palmitate oxidation (Fig 3B and 3C) or in the expression or phosphorylation of the metabolic enzymes FAS and AMPK (S5A Fig). Quantification of hepatic lipids indicated that TnTp38γ/δ^act^ livers had reduced triglycerides and NEFAs as compared with livers from TnTGFP mice at PD14 (Fig 3D). Notably, circulating ketone bodies, which are a feature of failing hearts [17,18], were also elevated in TnTp38γ/δ^act^ serum (Fig 3E), although we cannot rule out that they were being used by the heart as a source of energy. Additionally, blood circulating levels of triglycerides and NEFAs were elevated in TnTp38γ/δ^act^ mice (Fig 3F).

Under severe cardiac stresses, the heart can increase its fatty acid demand by stimulation of adipose tissue lipolysis [19]. In this regard, we found increased expression of PKA and increased p38 phosphorylation in white adipose tissue (WAT), both indicative of enhanced lipolysis [20], that correlated with increased phosphorylation of HSL in Ser660 in TnTp38γ/δ^act^ [21]. No changes in either phosphorylation or expression were detected for ACC or AMPK (S5B Fig). We therefore evaluated the impact of this cardiac metabolic shift on brown adipose tissue (BAT) thermogenesis. We found that TnTp38γ/δ^act^ mice presented lower BAT temperatures than TnTGFP mice (Fig 3G). Further, positron emission tomography (PET) analysis indicated reduced glucose uptake by BAT (Fig 3H), suggesting that cardiac metabolic requirements in TnTp38γ/δ^act^ mice significantly affected BAT functionality. In addition, cardiac metabolic alterations in TnTp38γ/δ ^act^ mice at PD14 led to a reduced body weight and fat mass (S5C and S5D Fig), suggesting that cardiac premature postnatal overexpression of p38γ/δ led to cardiomyocyte metabolic reprogramming and affected whole-body lipid metabolism.

**Fig 4:**
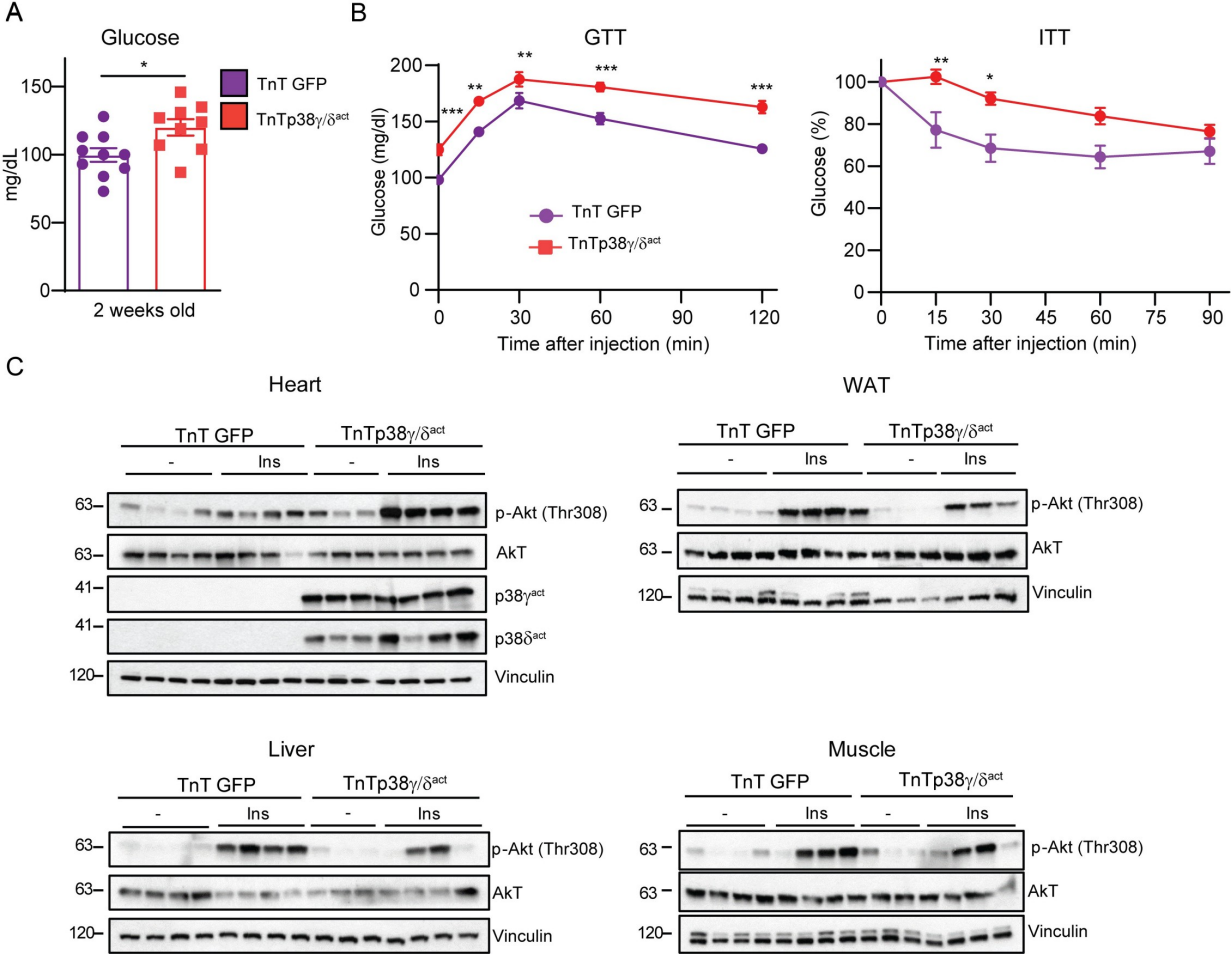
Cardiac-specific p38γ/δ^act^ overexpression leads to glucose intolerance and insulin resistance. Mice were treated as for Fig 3. **(A)** Blood glucose levels. **(B)** GTT and ITT. **(C)** Immunoblot analysis of insulin-stimulated Akt phosphorylation (at Thr308) in heart, WAT, liver, and skeletal muscle. Mice at PD14 were killed 15 min after IP insulin injection. Data are mean ± SEM (*n =* 7–10). *p < 0.05; **p < 0.01; ***p < 0.001 by Student *t* test or two-way ANOVA coupled to Tukey posttest. Raw data are given in S14 Fig. GTT, glucose tolerance test; ITT, insulin tolerance test; WAT, white adipose tissue.

We then evaluated the effect on whole-body glucose metabolism and insulin resistance. In TnTp38γ/δ^act^ mice at PD14, cardiac-specific overexpression of p38γ/δ increased blood glucose levels and insulin resistance (Fig 4A and 4B) but did not change blood insulin levels (S5E Fig). Increased phosphorylation levels of the serine/threonine kinase Akt upon insulin stimulation has been used as indicator of tissue insulin sensitivity [22]. We thus analyzed mice at PD14 after injecting with insulin and collected tissues 15 min later. Immunoblot analysis revealed elevated insulin-stimulated Akt phosphorylation in heart of TnTp38γ/δ^act^ mice as compared to wild-type mice (Fig 4C, S6A Fig), suggesting that reduced glycogen storage might lead to increased glucose uptake after insulin release. In contrast, insulin-stimulated Akt phosphorylation was strongly reduced in WAT, muscle, and liver (Fig 4C, S6B–S6D Fig), indicating the development of insulin resistance in peripheral tissues of TnTp38γ/δ^act^ mice (in line with the higher circulating levels of NEFAs and triglycerides in these mice; see Fig 3G).

To rule out that residual expression of p38γ/δ^act^ in muscle under the cTnT promoter was responsible for the observed systemic metabolic effects, we induced the expression of recombinant active p38γ/δ in newborn mice by injection at PD1 of AAV-Myf4-p38γ/δ_act_ (or AAV-Myf4-EGFP in control mice) and analyzed the mice at PD14 (S7A Fig). Notably, only having skeletal muscle–specific expression of recombinant active p38γ/δ after birth did not lead to the whole-body metabolic changes detected in TnTp38γ/δ^act^ mice at PD14 (S7B and S7C Fig). We concluded that cardiac-specific over expression of active p38γ/δ had a profound effect in cardiomyocyte metabolism and fuel usage, which triggered substantial whole-body metabolic alterations, including reduced BAT thermogenesis, dyslipidemia, hyperglycemia, and insulin resistance.

### p38γ/δ regulate early postnatal cardiac and whole-body metabolism

During HF, altered cardiomyocyte metabolism and insufficient energy supply can lead to cardiomyopathy [23]. We therefore analyzed whether the metabolic changes observed in TnTp38γ/δ^act^ hearts at PD14 were secondary to pathological dilated cardiomyopathy or causal for cardiac disease development. At PD7, TnTp38γ/δ^act^ mice had normal-sized hearts with no fibrosis and showed no signs of eccentric cardiac hypertrophy; however, their cardiomyocytes already presented low glycogen levels (S8A–S8D Fig). Moreover, these mice had normal cardiac functions (S8E Fig), albeit with some metabolic alterations (e.g., elevated blood glucose; S8F Fig) but without changes in circulating triglycerides or NEFA (S8G Fig). This early appearance of metabolic alterations at PD7 in TnTp38γ/δ^act^ mice (e.g., reduced cardiac glycogen storage and altered glucose metabolism) suggested that these changes led to cardiac disease development.

### Premature postnatal activation of p38γ/δ kinases in cardiomyocytes causes permanent cardiac dysfunction

**Fig 5:**
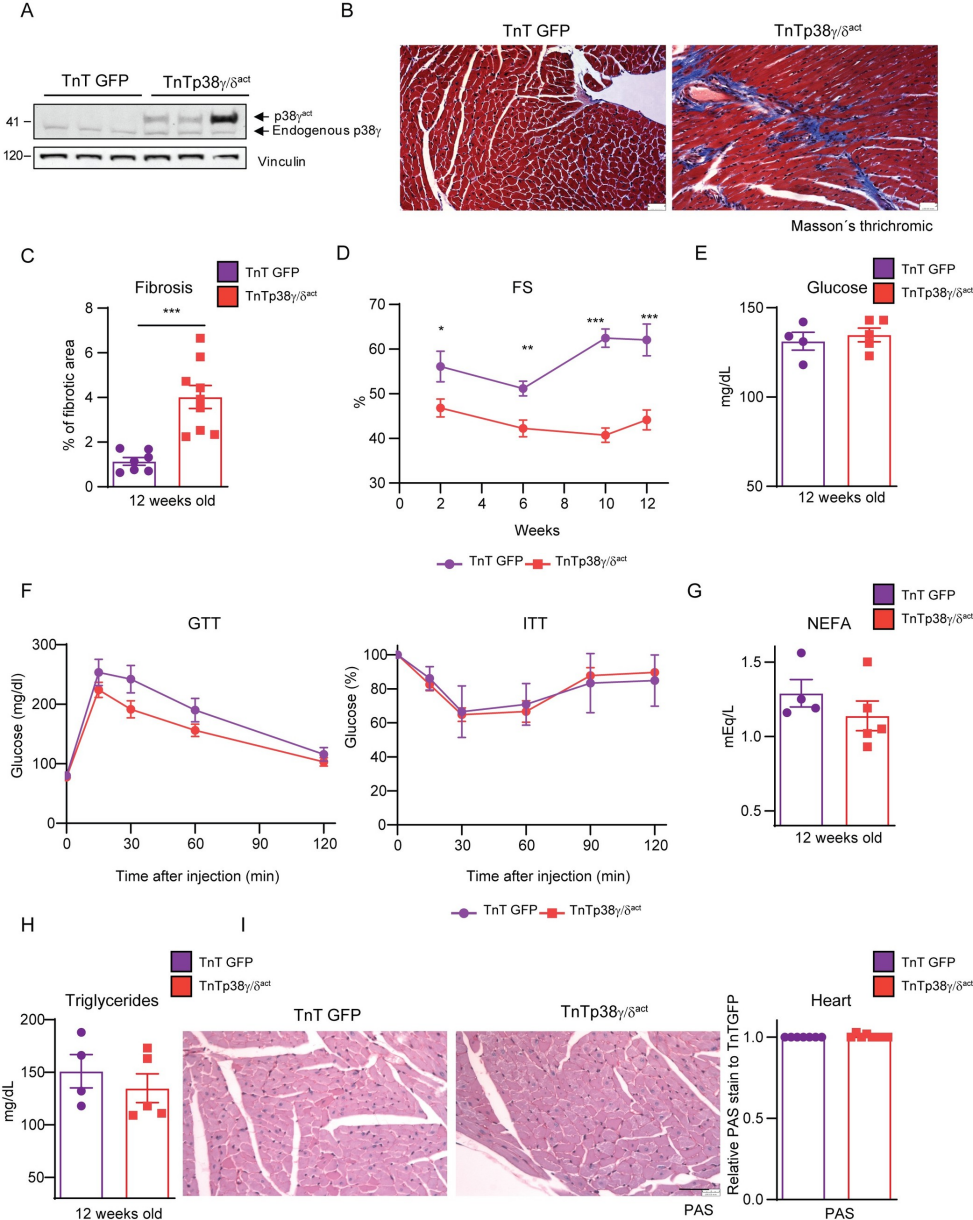
Postnatal cardiac-specific p38γ/δ^act^ overexpression leads to heart defects that persist throughout life with no metabolic consequences in adulthood. Mice were IV injected at PD1 with AAV-cTnT-GFP-Luc (TnTGFP) or AAV-cTnT-p38γ/δ_act_ (TnTp38γ/δ^act^), analyzed for phenotype progression over 12 weeks, and then killed. **(A)** Immunoblot analysis of exogenous active (p38γ^act^) and endogenous p38γ in hearts extracts of 12-week-old mice. **(B, C)** Masson’s trichrome staining in heart sections from 12-weeks-old mice (**B**), and quantification (**C**). Scale bar: 200 μm. **(D)** Left ventricular FS progression from weeks 2 to 12**. (E)** Plasma glucose. **(F)** GTT and ITT. **(G)** Plasma NEFA. **(H)** Plasma triglycerides. **(I)** Representative PAS staining of heart sections with its respective quantification relative to TnTGFP mice. Scale bar: 200 μm. Data are mean ± SEM (*n =* 4–8). *p < 0.05; **p < 0.01; ***p < 0.001 by two-way ANOVA coupled to Tukey posttest or Student *t* test. Raw data are given in S14 Fig. FS, fractional shortening; GTT, glucose tolerance test; ITT, insulin tolerance test; NEFA, non-esterified fatty acid; PAS, periodic acid–Schiff.

We next evaluated whether the pathological effects of premature postnatal p38γ/δ^act^ expression persisted in adulthood. Hearts from adult (12-weeks-old) TnTp38γ/δ^act^ mice still expressed exogenous active p38γ and p38δ (Fig 5A, S9A Fig) and exhibited significantly increased left ventricular fibrosis (Fig 5B and 5C) and systolic dysfunction (Fig 5D). TnTp38γ/δ^act^ mice also had below-normal exercise capacity, as measured by maximum running time and exercise endurance (S9B Fig). Despite the persistent altered cardiac function, the whole-body metabolic alterations detected in mice at PD14 were resolved by this stage: adult TnTp38γ/δ^act^ mice had normal ranges of blood glucose levels, glucose tolerance, and insulin sensitivity (Fig 5E and 5F). Moreover, compared to TnTGFP control mice, no changes were detected in plasma NEFA and triglycerides levels, or cardiac glycogen content (Fig 5G–5I). Thus, premature p38γ/δ signaling activation in neonatal cardiomyocytes had permanent deleterious effects on heart function, whereas the deregulated body metabolism resolved once the cardiomyocyte metabolic shift occurs.

### Early postnatal cardiac-specific p38γ/δ deletion increases cardiac glycogen storage and affects whole-body metabolism

**Fig 6:**
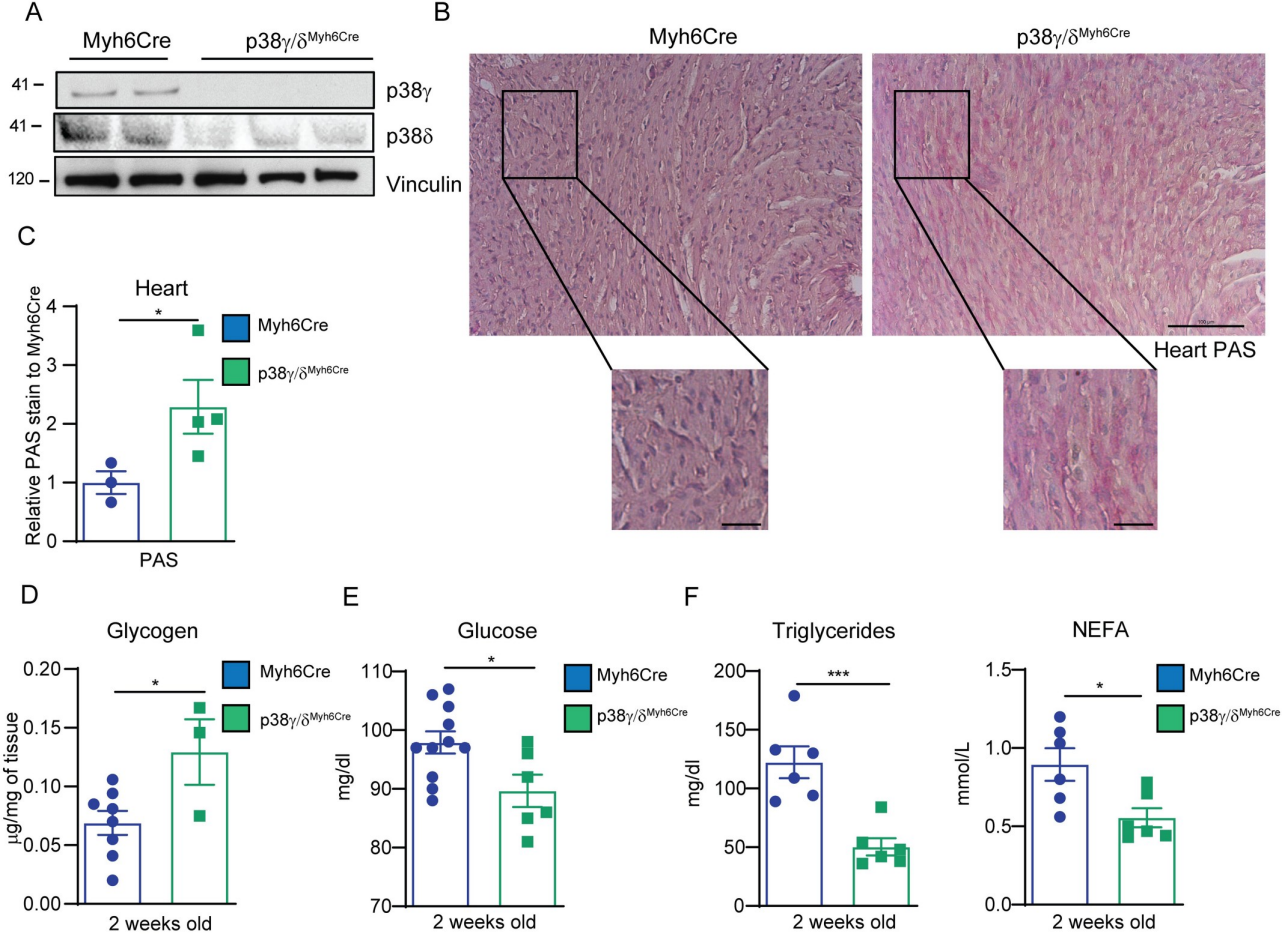
Early postnatal cardiac-specific p38γ and p38δ deletion increases cardiac glycogen storage and affects whole body metabolism. Mice were IP injected with tamoxifen (p38γ/δ ^Myh6-Cre^) or vehicle (Myh6-Cre; control mice) at PD1, PD2, and PD3, and killed at PD14. **(A)** Immunoblot analysis of p38γ and p38δ in heart extracts. **(B, C)** Representative images of PAS staining of heart sections (**B**) and its quantification, normalized to Myh6-Cre (**C**). Scale bar: 100 μm and 25 μm (amplification). **(D)** Glycogen quantification. **(E)** Blood glucose levels. **(F)** Plasma triglycerides and NEFA. Data are mean ± SEM (*n =* 4–6). *p < 0.05; ***p < 0.001 by Student *t* test. Raw data are given in S14 Fig. NEFA, non-esterified fatty acid; PAS, periodic acid–Schiff.

To verify a direct involvement of p38γ/δ in the regulation of cardiac glycogen storage in the neonatal heart, we evaluated the effects of p38γ/δ deletion with a specific cardiomyocyte tamoxifen-inducible Cre recombinase system (p38γ/δ^Myh6Cre^). Heart deletion of p38γ/δ was confirmed after tamoxifen treatment (Fig 6A). p38γ/δ^Myh6Cre^ mice at PD14 presented increased cardiac glycogen levels (Fig 6B–6D). These cardiac effects were associated with decreased levels of glucose, NEFA, and triglycerides in blood (Fig 6E and 6F). Overall, our data demonstrated that p38γ/δ kinases control cardiac glycogen storage and highlight that their dysregulation impacted the whole-body metabolic state.

### p38γ/δ phosphorylation of GYS1 promotes GYS1 phosphorylation by GSK3

**Fig 7:**
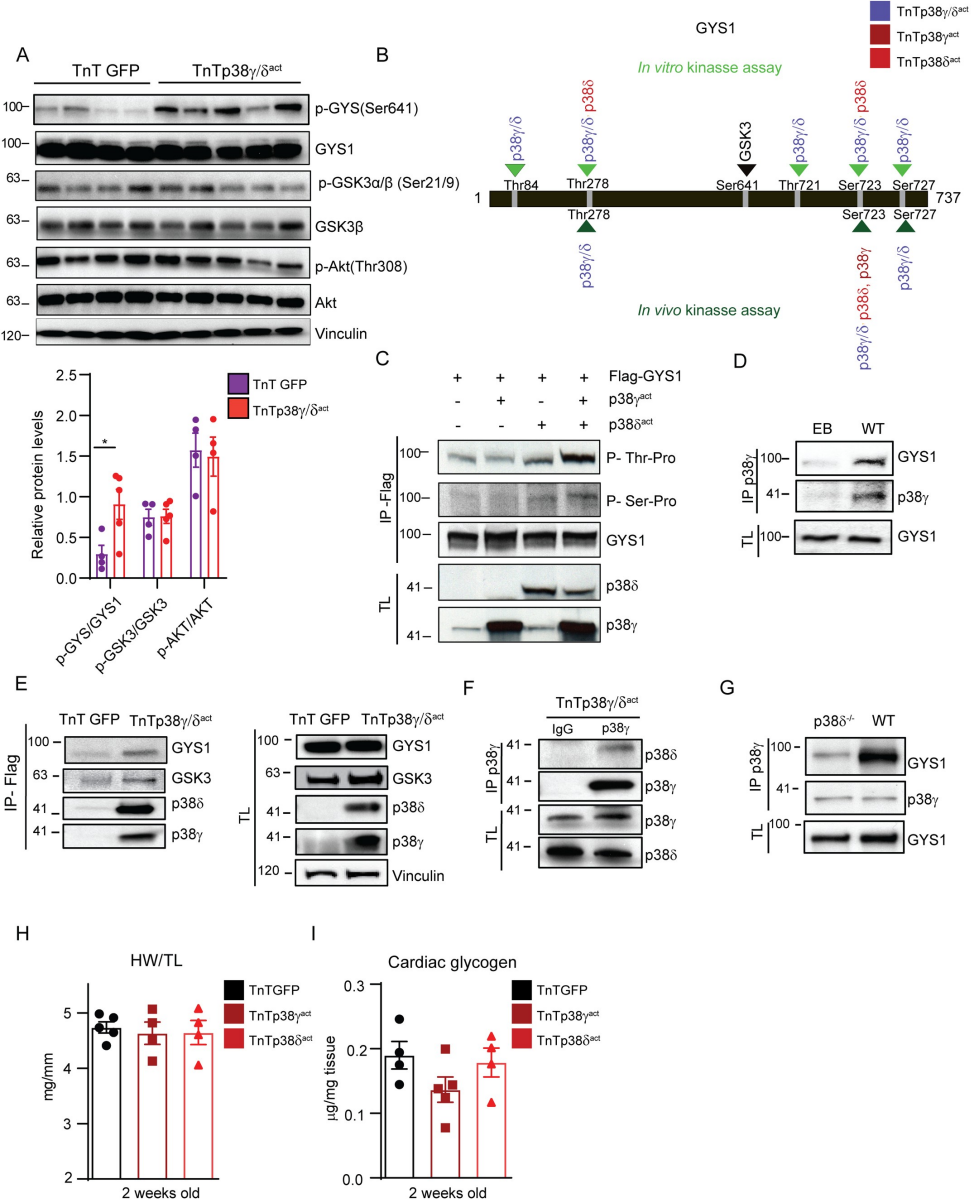
p38γ/δ cooperatively interact with GYS1 and GSK3 to promote GYS1 phosphorylation at its canonical site (Ser641). **(A)** Immunoblot analysis of the Akt-GSK3–GYS axis in cardiac homogenates from AAV-cTnT-GFP-Luc (TnTGFP) or AAV-cTnT-p38γ/δ_act_ (TnTp38γ/δ^act^) mice killed at PD14 with its respective quantification (lower panel). **(B)** Scheme showing GYS1 sites phosphorylated by p38γ, p38δ, or both, in an in vitro kinase assay (top, light green) or an in vivo kinase assay in HEK-293 cells (bottom, dark green). The GYS1 canonical site for GSK3 phosphorylation is Ser641 (shown in bold). Data are representative of at least 3 independent experiments (biological replicates). **(C)** In vivo phosphorylation of GYS1 in HEK-293 cells that had been transfected with Flag-GYS1 alone or together with p38γ^act^ or p38δ ^act^, or both. Phosphorylation of transfected GYS1 was evaluated in the Flag-immunoprecipitate with phospho-MAPK substrates for Ser or Thr. TL before immunoprecipitation is shown as control. **(D)** Immunoprecipitation and immunoblot analysis of GYS1 and endogenous p38γ association in WT mice. **(E)** Immunoblot analysis of GYS1 and GSK3 in Flag-p38γ/δ^act^ immunoprecipitates from heart lysates, to detect interactions between these proteins and the exogenous p38γ/δ^act^. **(F)** Immunoprecipitation and immunoblot analysis of the association between p38δ and p38γ in TnTp38γ/δ^act^ mice. **(G)** Immunoprecipitation/immunoblot analysis of the interactions between GYS1 and endogenous p38γ in WT or p38δ^−/−^ mice. **(H, I)** Analysis of hearts from mice that were IV injected at PD1 with AAV-cTnT-GFP-Luc (TnTGFP), AAV-cTnT-p38γ_act_ (TnTp38γ^act^), or AAV-cTnT-p38δ_act_ (TnTp38δ^act^) and killed at PD14, showing (**H)** HWTL ratio and (**I)** cardiac glycogen content. Data are mean ± SEM (*n =* 10). One-way ANOVA coupled to Tukey posttest or Student *t* test. Raw data are given in S14 Fig. EB, empty bead; GSK3, glycogen synthase kinase-3; GYS1, glycogen synthase 1; HWTL, heart weight to tibia length; TL, total lysate; WT, wild-type.

GYS1, which is responsible for glycogen synthesis, is inactivated by phosphorylation at its canonical site Ser641 (p-Ser641) by glycogen synthase kinase-3 (GSK3) [24]. GSK3, in turn, is activated by phosphorylation by the Akt kinase, which facilitates GSK3’s interaction and phosphorylation of its substrates [25]. Of note, in TnTp38γ/δ^act^ mice at PD14, the phosphorylation levels of GYS1 at Ser641 were increased compared with controls, while the phosphorylation levels of GSK3 and its upstream kinase Akt remained unchanged (Fig 7A). This suggested that another route besides the canonical phosphorylation pathway led to increased p-Ser641-GYS1 levels. We thus evaluated whether p38γ/δ can directly phosphorylate GYS1 (and act as a priming site for GSK3) (S10 Fig) in an in vitro kinase assay followed by MS/MS analysis. These analyses revealed that (i) p38γ alone was not sufficient to phosphorylate GYS1 (at any site); (ii) p38δ alone phosphorylated GYS1 at Ser723 and Thr278; and (iii) p38γ and p38δ together phosphorylated GYS1 at 5 residues (Thr84, Thr721, Ser723, Ser727, and Thr278) (Fig 7B, S11A Fig). Indeed, in HEK-293 cells transfected with GYS1 and constitutively active p38γ and/or p38δ, GYS1 was phosphorylated at its p38MAPK canonical (Ser/Thr-Pro) residues to a low degree when only active p38δ was present, and to a higher degree when both active p38γ and p38δ were present, as shown by immunoprecipitation and immunoblot analyses (Fig 7C). Immunoprecipitation of GYS1 protein from HEK-293 transfected cells followed by MS/MS confirmed that the both kinases together phosphorylated GYS1 at Ser723, Ser727, and Thr278 in vivo, while having either p38γ or p38δ alone, only phosphorylated Ser723 (Fig 7B, S11B and S11C Fig). Furthermore, immunoprecipitation of p38γ from heart lysates of wild-type (non-infected mice) showed that the cardiac GYS1 protein physically interacts with p38γ, reinforcing the hypothesis that p38γ (and likely p38δ) have a role in regulating the GYS1 protein in the heart (Fig 7D). Overall, these results suggested that p38γ and p38δ work cooperatively (perhaps as a complex) to phosphorylate GYS1.

We next evaluated whether phosphorylation of GYS1 residues by p38γ/δ acts as a priming event to facilitate GSK3 interactions with GYS1 (and thereby promote GYS1 phosphorylation at Ser641). A FLAG-pulldown using recombinant FLAG-p38γ^act^ or FLAG-p38δ^act^ coprecipitated GYS1 and GSK3 from heart lysates of TnTp38γ/δ^act^ mice but not from control (TnTGFP) mice, indicating that the p38γ and p38δ proteins physically interact in vivo and are part of the same protein complex (Fig 7E). These results suggest that p38γ/δ might work as a docking platform for mediating the posttranslational modification of GYS1 by GSK3.

Notably, in TnTp38γ/δ^act^ mice, cardiac p38δ coimmunoprecipitated with p38γ (Fig 7F), further supporting the hypothesis that these 2 proteins form a complex. Furthermore, GYS1 coimmunoprecipitated with p38γ at PD14 from hearts of wild-type mice but not of p38δ^−/−^ mice (Fig 7G), showing that p38δ was required for a stable GYS1–p38γ interaction. Additionally, increased phosphorylation of GYS1 at Ser641 was only induced if both p38γ/δ kinases were prematurely activated in cardiomyocytes in neonatal hearts, but not if only one was activated (using TnTp38γ^act^ or TnTp38δ^act^) (S12A Fig).

To further evaluate the possibility that both kinases act in a cooperative manner, we studied the effects of each kinase alone. Neither TnTp38γ^act^ nor TnTp38δ^act^ mice at PD14 showed pathological hypertrophy (Fig 7H) or alterations in heart glycogen deposition (after infection at PD1) (Fig 7I, S12B Fig). Moreover, glucose and lipid metabolic parameters were unchanged in TnTp38γ^act^ or TnTp38δ^act^ mice at PD14 as compared to control mice, except for increased blood basal glucose levels (S12C–S12E Fig). These data are in concordance with both kinases being required to phosphorylate and inactivate cardiac GYS1, resulting in reduced cardiac glycogen storage and whole-body metabolic changes.

### Postnatal GYS1 deletion leads to whole-body metabolic alterations

**Fig 8:**
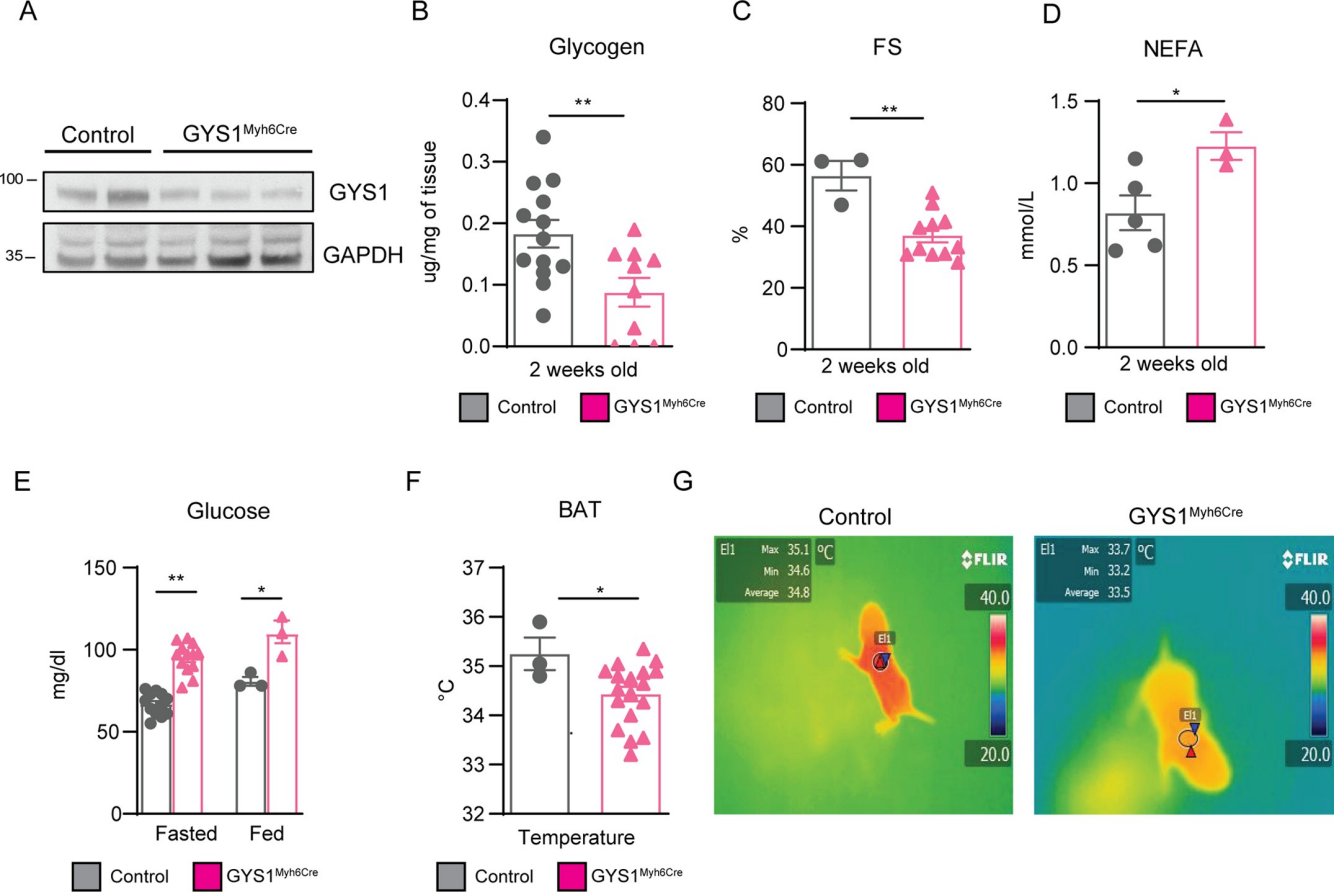
Postnatal GYS1 deletion leads to whole-body metabolic alterations and cardiac dysfunction. *Gys1*^Myh6Cre^ or Myh6-Cre (control) mice were IP injected with 62.5 mg/kg tamoxifen at PD1, PD2, and PD3, and killed at PD14. **(A)** Immunoblot showing partial GYS1 deletion in heart extracts. **(B)** Heart glycogen content. **(C)** Echocardiography-measured FS. **(D)** NEFA plasma levels. **(E)** Blood plasma glucose in fed or fasted (e.g., food deprived for 4 h) conditions. **(F, G)** BAT temperature chart and representative thermographic images mice at PD14. Data are mean ± SEM (*n =* 3–17). *p < 0.05; **p < 0.01; ***p < 0.001 by Student *t* test or two-way ANOVA coupled to Tukey posttest. Raw data are given in S14 Fig. BAT, brown adipose tissue; FS, fractional shortening; GYS1, glycogen synthase 1; NEFA, non-esterified fatty acid.

We next used the conditional *Gys1* mouse line [26] to generate mice with specific cardiomyocyte inducible deletion of GYS1 (*Gys1*^Myh6Cre^), to further investigate the effects of the dysregulated p38γ/δ activation on whole-body metabolism. We achieved GYS1 deletion by 3 consecutive tamoxifen IP injections starting at PD1 (Fig 8A). *Gys1*^Myh6Cre^ mice presented the following: (i) reduced cardiac glycogen storage and impaired cardiac function (Fig 8B and 8C); (ii) increased NEFA and glucose circulating levels (Fig 8D and 8E); and (iii) impaired BAT thermogenesis (Fig 8F and 8G). Altogether, these results suggested that defects in glycogen cardiac metabolism led to whole-body metabolic alterations, similar to the alterations observed in TnTp38γ/δ^act^ mice.

### Maternal HFD feeding suppresses cardiac dysfunction in pups overexpressing p38γ/δ^act^

**Fig 9:**
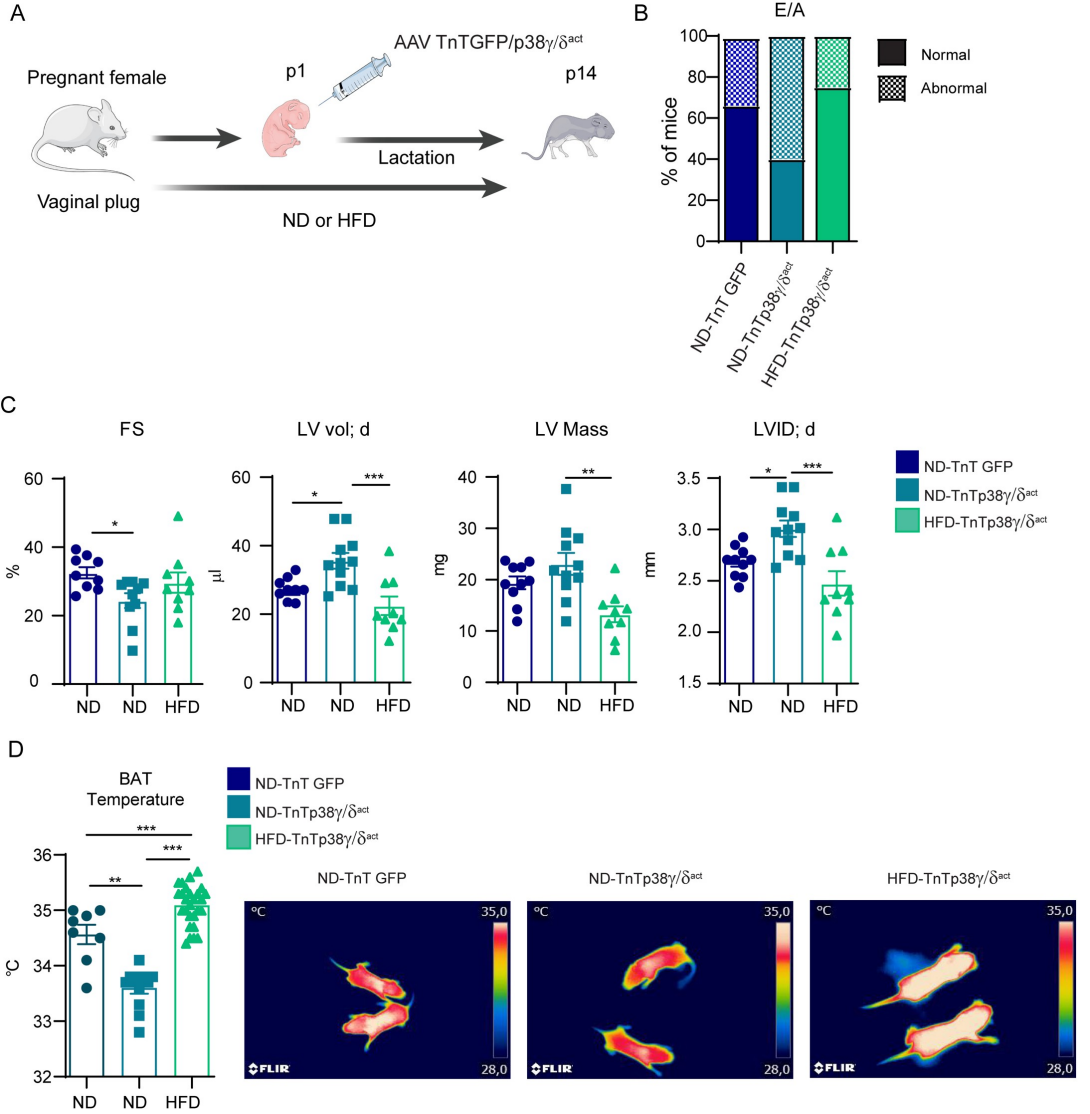
Maternal HFD feeding suppresses cardiac dysfunction in pups overexpressing p38γ/δ^act^. **(A)** Schematic protocol: CD1 females were crossed; after pregnancy confirmation by vaginal plug appearance, they were fed a HFD for the entire experiment (e.g., pregnancy and lactation). Neonates were IV injected at PD1 with AAV-cTnT-GFP-Luc (TnTGFP) or AAV-cTnT-p38γ/δ_act_ (TnTp38γ/δ^act^); during their lactation, mother remained on the same diet (e.g., ND or HFD) as during pregnancy. Pups were killed at PD14. **(B)** Percentage of mice at PD14 with normal or abnormal mitral valve flow (E/A) as an indicator of diastolic dysfunction. **(C)** Echocardiography measured parameters. (D) BAT temperature chart and representative thermographic images from 2-week-old mice. Data are mean ± SEM (*n =* 9 or 10). *p < 0.05, **p < 0.01, ***p < 0.001 by one-way ANOVA coupled to Tukey posttest or chi-squared test. Raw data are given in S14 Fig. The figure was prepared using Servier Medical Art (https://smart.servier.com/). BAT, brown adipose tissue; FS, fractional shortening; HFD, high-fat diet; LVID; d, left ventricular internal diameter in diastole; LV mass, left vetricular mass; LVvol; d, left ventricular volume in diastole; ND, normal diet.

Can a metabolic intervention rescue/prevent the deleterious cardiovascular effects of overexpressing p38γ/δ (which creates deficient cardiac glycogen storage)? To test this idea, we fed mouse mothers a high-fat diet (HFD, with 60% kcal derived from fat) starting from pregnancy confirmation until PD14 of lactating pups (Fig 9A); note that, at PD14, TnTp38γ/δ^act^ hearts show an increased dependency on fatty acids (rather than glycolysis) as an energy source. We then evaluated pup hearts by echocardiography at PD14, with maternal (M)–normal diet (ND) or HFD-fed TnTp38γ/δ^act^ mothers or M-ND-fed TnTGFP mothers (as a control). Strikingly, the diastolic function, measured by early (E) to late (A) ventricular filling velocities (E/A), was normal for the M-HFD-TnTp38γ/δ^act^ pups, as compared to the M-ND-TnTGFP (control) pups, but showed increased frequency of abnormal function for M-ND-TnTp38γ/δ^act^ pups (Fig 9B). Further, heart measurements from M-HFD-TnTp38γ/δ^act^ pups were indistinguishable to those from M-ND-TnTGFP pups, including diastolic ventricular diameter (LVID;d), left ventricular volume in diastole (LVvol;d), and FS (indicative of a protection against systolic dysfunction) (Fig 9C). Furthermore, BAT thermogenesis was normal in the M-HFD-TnTp38γ/δ^act^ pups (and impaired in the M-ND-TnTp38γ/δ^act^ pups) (Fig 9D). Finally, the cardiac function was similar between the M-HFD-TnTp38γ/δ^act^ and M-ND-TnTGFP pups (S13 Fig). Overall, these data indicated that M-HFD feeding during pregnancy and lactation prevented the development of cardiac dysfunction due to premature activation of the p38γ/δ signaling in cardiomyocytes; in other words, the anatomical and functional consequences of defects in cardiac glycogen storage and thermogenesis in heart can be metabolically circumvented.

## Conclusions

Cardiomyopathies are functional and structural disorders of the heart. In infants, around 5% to 26% of cardiomyopathies are related to inborn errors of metabolism, with glycogen storage diseases (GSDs), mitochondrial dysfunction, and lysosomal or fatty acids disorders associated to cardiac dysfunction in infancy [27,28]. However, genes related to infant cardiomyopathies must first be identified as a first step for personalized management and therapy [29]. In addition, a switch in cardiac metabolism appears at the same time that loss of the regenerative potential of the mammalian heart, suggesting that metabolism controls cell proliferation and differentiation. Moreover, following injury, the incapacity to regenerate correlates with a metabolic shift from fatty acid oxidation to glycolysis. Thus, understanding the mechanisms that regulate cardiac metabolism is key to developing metabolic interventions during disease, regeneration, and development [30].

**Fig 10:**
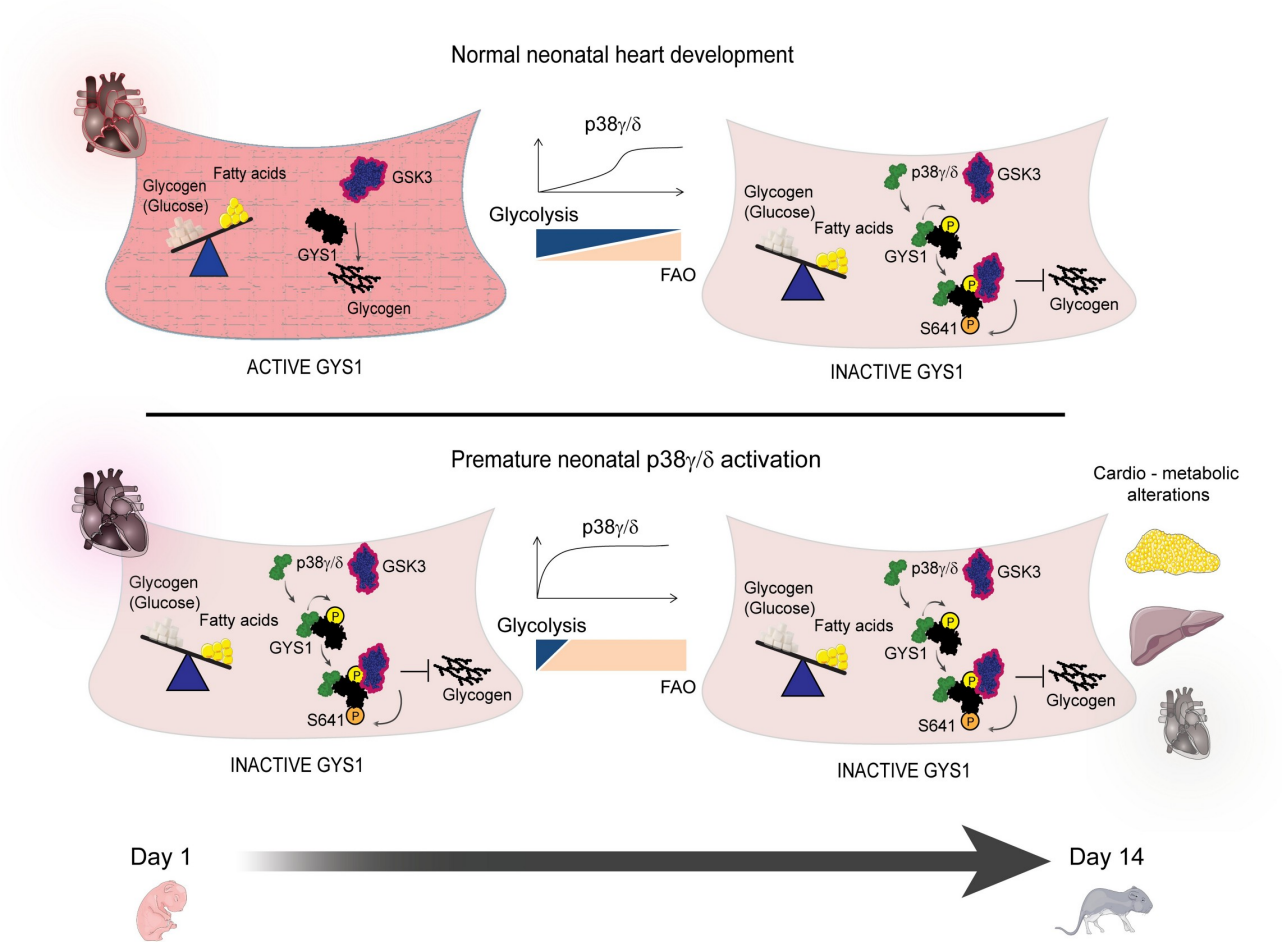
Early cardiac p38γ/δ expression inactivates GYS1 affecting cardiac and whole-body metabolism, but the effects can be mitigated by metabolic intervention. Schematic overview of our findings At early postnatal development, p38γ/δ are not present in heart and in consequence, they did not facilitate GYS phosphorylation and inactivation by GSK3 allowing glycogen storage. Premature postnatal cardiac-specific p38γ/δ overexpression triggers a premature metabolic switch to fatty acid oxidation, with whole-body metabolic alterations, including insulin resistance, glucose intolerance, altered hepatic lipid metabolism, and impaired thermogenesis, as well as permanent cardiac defects. The 2 kinases, p38γ/δ, work collaboratively to control cardiac glycogen storage by regulating GYS1’s interaction with its inhibitory kinase GSK3. Maternal metabolic intervention by HFD feeding during pregnancy and lactation mitigated the cardiac dysfunction and impaired thermogenesis in offspring, setting a precedent for treatment of neonatal cardiometabolic genetic diseases. The figure was prepared using Servier Medical Art (https://smart.servier.com/). FAO, fatty acid oxidation; GSK3, glycogen synthase kinase-3; GYS1, glycogen synthase 1; HFD, high-fat diet.

We have previously shown that p38γ/δ expression is low in cardiomyocytes at birth and sharply increases during the first 2 weeks of life [15], in parallel with changes in its fuel used by the heart, from glucose to fatty acid oxidation [15,31]. We have now found that the p38γ/δ kinases in heart modulate this transition, which is normally associated with cardiomyocyte maturation during postnatal heart development. Premature expression and activation of cardiac p38γ/δ resulted in alteration of heart glycogen deposition, which induced severe cardiomyopathy and altered the whole-body metabolism. Our results demonstrated that early postnatal cardiac expression and activation of p38γ/δ induced GYS1 phosphorylation at its GSK3 priming site, favoring a GYS1/GSK3 interaction—and consequentially, GYS1 phosphorylation at Ser641—by GSK3 (Fig 10). We observed that reduction in the cardiac glycogen storage drives cardiomyocyte metabolism toward a premature use of fatty acids, resulting in decreased cardiac lipid storage and elevated circulating levels of ketone bodies, triglycerides, and NEFAs, which suggests an increased adipose tissue lipolysis. High lipid demand in heart resulted in deficient BAT thermogenesis in TnTp38γ/δ^act^ mice, possibly due to reduced glucose uptake by this tissue, as shown by PET analyses and an increased lipid utilization by the heart.

### p38γ and p38δ control postnatal heart metabolic switch through GYS1 inactivation

The heart is one of the highest energy consumer organs in mammals and needs high amount of energy as soon as its first beats in utero. During fetal development, the heart’s energy metabolism relies essentially on carbohydrates, and the accumulation of glycogen in cardiomyocytes serves as a metabolic reserve in order to deal with the increased energy demands. Soon after birth, the glycogen pool in cardiomyocytes is significantly reduced (approximately 30% of cell volume in fetal cardiomyocytes, relative to 2% in adult cardiomyocytes), and the energy substrate metabolism rapidly switches to fatty acids oxidation in order to adjust to the new body requirements, substrate availability, oxygen pressure and cardiac workload [19]. Various signaling pathways, including insulin through AKT-GSK3 mediated inhibition, the β-adrenergic cascade through activation of PKA, and AMPK inhibition, have been implicated in the regulation of glycogen storage and fatty acid oxidation [32–35]. Moreover, exposure of cardiomyocytes to oxygen after birth leads to instability of hypoxia-inducible factor (HIF), triggering mitochondrial biogenesis and activation of lipid oxidation [36]. However, the contribution and regulation of these pathways during the early postnatal cardiac metabolic switch were not clear. The biological importance of a timely regulation of cardiac glycogen levels in cardiac development and function has been highlighted in previous studies in humans and in mouse models, in which alterations in glycogen metabolism genes led to heart disease [37,38]. Disruption of GYS1 during embryonic development leads to abnormal cardiac development and function [39]. In addition, impaired glycogen use due to mutations in enzymes involved in glycogen degradation (such as occurs in Pompe disease or GSDIII) has also been associated with cardiomyopathy and fibrosis, similar to the phenotype we observed in our animals [39–41]. This fibrosis is a hallmark of HF and has been related to cardiomyocyte death and replacement of lost cardiomyocytes by fibrotic material [42]. Therefore, glycogen metabolism seems to have a clear role in heart functionality. A better understanding of signaling pathways that regulate glycogen metabolism in cardiomyocytes has the potential to (i) give insight about underlying mechanisms of congenital heart disease; (ii) provide new therapeutic targets for infant cardiomyopathies; (iii) be used for regenerative therapies; and (iv) increase our understanding of "cardiac flexibility" in adapting to heart injury [43].

Previous work from our laboratory has shown that the alternative SAPKs, p38γ and p38δ, increase their expression and activity soon after birth, coinciding with the time window of key metabolic and structural changes that occur in cardiomyocytes [15]. p38γ and p38δ regulate tissue homeostasis at multiple levels through the phosphorylation of diverse substrates, such as SAP90, SAP97, and DEPTOR [14,44–46]. Recent evidence suggests that these kinases cooperate in the phosphorylation of some of their substrates [15,47]. Here, we identified a previously unreported p38γ/δ substrate, GYS1, which is the key enzyme in muscle glycogen synthesis. p38γ/δ activation during the first weeks after birth leads to GYS1 inhibitory phosphorylation and reduced glycogen accumulation in heart, at a time when cardiomyocytes switch into fatty acid oxidation as a main energy source. In vitro and in vivo kinase assays indicated a direct and cooperative function of p38γ/δ kinases in the phosphorylation of several GYS1 residues (e.g., Thr278, Ser723, and Ser727). Forced premature induction of p38γ/δ activation in cardiomyocytes of newborn mice results in reduced cardiac glycogen content due to early GYS1 phosphorylation and inhibition of cardiac glycogen production in vivo. On the other hand, deletion of these kinases leads to increased cardiac glycogen storage. Our results also suggested that the p38γ/δ complex acts as a docking platform, thereby coordinating the posttranslational modification (and inactivation) of GYS1 by GSK3. Further research is required to determine the exact physiological role of these interactions.

Our previous reports have demonstrated that p38γ/δ can regulate cardiomyocyte hypertrophic growth through the activation of the mTOR pathway [15], which has a key role inducing mitochondrial biogenesis and fatty acids oxidation [48]. Overall, these results suggest that p38γ/δ kinases may be key regulators of the postnatal metabolic switch, acting not only by coordinating the inhibition of glycogen storage, but also by promoting fatty acid utilization.

### Heart metabolism affects whole-body metabolism

Our findings suggest that cardiomyocytes are key regulators of whole-body metabolism during early postnatal development, which may be linked to the heart’s high-energy demand at this stage of rapid growth. Forced premature induction of p38γ/δ activation in cardiomyocytes led to inhibition of cardiac glycogen synthesis during the first weeks after birth, resulting in an early metabolic shift to lipid oxidation that induced a deficit in cardiomyocyte fuel supply—and ultimately, a whole-body metabolic deregulation with increased levels of circulating triglycerides, NEFA, and ketone bodies. We demonstrated that TnTp38γ/δ^act^ hearts had increased rates of fatty acid oxidation; however, it remains to be determined whether these hearts are also using ketone bodies as an energy source, similar to failing hearts [17,18].

To confirm the link between glycogen synthesis inhibition and whole-body metabolic alterations, we depleted GYS1 specifically in cardiomyocytes during early postnatal development. This led to a decreased cardiac function and deregulation of whole-body metabolism—the same phenotype obtained when we inactivate GYS1 by overexpressing the kinases p38γ and p38δ. Therefore, our results demonstrated that cardiac glycogen storage is (i) crucial for the correct cardiac development in the early postnatal period; and (ii) necessary for the heart’s contractile function. This highlights the importance of heart as a metabolic tissue in the postnatal period. Mechanisms underlying the crosstalk between heart and other tissues that affect whole-body metabolism could involve release of cardiokines. However, the cardiokines identified to date seem to act in an autocrine manner to regulate cardiac function and response to stress, with at least 2 examples of cardiokines that regulate systemic metabolism: the atrial natriuretic peptide (ANP) and the B-type/ventricular natriuretic peptide (BNP), both of which lead to browning of WAT [49].

The high fatty acid content of maternal milk in many species effectively provides for the high energy demand of the newborn heart [50,51]. Moreover, milk triglyceride and insulin levels are elevated in HFD dams at weaning, and they can affect the offspring metabolism [52]. In addition, HF induced by energy deficits might be prevented by feeding the animals with HFD [7]. Here, we show that in neonatal mice with premature induction of p38γ/δ activation in cardiomyocytes, the deficit in cardiomyocyte fuel supply was overcome by increasing the fatty acid content of maternal milk through maternal HFD feeding. Importantly, this metabolic intervention by maternal HFD feeding circumvented the cardiac dysfunction in pups. This provides evidence that the shortage of cardiac glycogen per se was responsible for the cardiac malfunction and that administration of an alternative lipid energy source can lead to functional recovery. However, since most cardiomyopathies are identified after birth, and most at later stages, future approaches may confirm the pathological reversion that we have observed. Of note, HFD interventions are currently used to treat other GSDs, such as GSDIII; however, there is not much information regarding cardiac impact [41]. Moreover, we showed that HFD feeding rescued impaired BAT thermogenesis, suggesting that the whole-body metabolic deregulation also comes from cardiac energy deficiency. Thus, understanding the molecular regulators of cardiac glycogen storage, and the tissue metabolic demands derived from its deficiency, could be crucial to find a possible treatment for these diseases.

In sum, we identified p38γ/δ as novel regulators of GYS1 activity and cardiac glycogen metabolism, highlighting the biological relevance of p38γ and p38δ in cardiac energy metabolism and function. Importantly, we also demonstrated the importance of cardiac glycogen during the early postnatal period and the requirement of its presence to guarantee the cardiac functionality and to maintain correct whole-body metabolism (Fig 10). Our findings may help us to better understand the mechanisms behind some adult cardiomyopathies with unknown genetic basis, with these kinases being potential therapeutic targets for treating congenital cardiac metabolic disorders. Further analysis of the function of these kinases in infant cardiomyopathies, and their role as metabolic cardiac regulators in MI and HF, need to be explored. Finally, our results suggest that cardiac genetic diseases associated with metabolic dysfunction might be treated with maternal diet intervention, setting a precedent for the treatment of congenital cardiometabolic disorders.

## Materials and methods

### Mice

Mice deficient for p38γ (B6.129-Mapk12tm1) or p38δ (B6.129-Mapk13tm1) [47] were backcrossed for 10 generations to the C57BL/6J background (Jackson Laboratory). CD1 mice were IV injected with 0.5 × 10^11^ viral particles encoding human p38γ and p38δ active mutants [53] under the control of the cTNT promoter or the myog promoter, in order to get cardio-specific or muscle-specific overexpression, respectively. Control mice were injected with virus encoding GFP luciferase.

For the HFD experiment, pregnant CD1 mice were fed a normal diet containing 22% fat, 28% proteins, and 50% carbohydrates (ND, Altromin, Ref.1410) or a HFD with 60% fat, 20% protein, and 20% carbohydrates (HFD with 60% kcal derived from fat, of 5% soy bean and 55% lard; D11103002, Research Diets) after positive plug confirmation and maintained during lactation until pups were killed at PD14.

For the tamoxifen-inducible cardio-specific deletion of both p38γ/δ or GYS1, transgenic mice B6.FVB(129)-A1cfTg(Myh6-cre/Esr1*)1Jmk/J from Jackson Laboratory [54] were used to cross with p38γ/δ [55] or GYS1 [26] flox mice. Tamoxifen was injected IP (62.5 mg/kg) for 3 consecutive days starting at PD1. Control mice for the tamoxifen experiments were injected with corn oil. MI surgeries were performed on PD7. Lateral thoracotomy at the fourth intercostal space was performed. A tapered needle attached to a 6–0 prolene suture (Ethicon) was passed through the midventricle below the origin of the left anterior descending coronary artery and tied to induce infarction [56]. All experiments were performed in fed condition unless specified. All animal procedures conformed to EU Directive 86/609/EEC and Recommendation 2007/526/EC regarding the protection of animals used for experimental and other scientific purposes, enacted under Spanish law 1201/2005. The project was evaluated by the Research Ethics Committee of the Autonomous University of Madrid (Approval number CEI 94-1704-A275).

### Histology

Tissue samples were fixed in 10% formalin for 48 h, dehydrated, and paraffin embedded. Sections (8 μm) were cut and stained with hematoxylin and eosin (H&E) (American Master Tech Scientific) or PAS (Sigma). Fat droplets were detected by ORO staining (0.7% in propylene glycol) in 8-mm slides in OCT compound (Tissue-Tek). Fibrosis was assessed with Masson’s trichrome staining. PAS, ORO, and Masson’s trichrome staining were quantified as percentage of stained area in relation to total tissue area using a custom-made macro in ImageJ. PAS was quantified as percentage of stained area relative to the controls TnTGFP or mhcCre. For wheat germ agglutinin (WGA) immunofluorescence, 8-μm heart sections were prepared, washed in PBS 1×, incubated for 2 h in WGA-Alexa 488 lectin (Invitrogen, Carlsbad, California), washed, and mounted in anti-fade reagent. Four images (20×) were taken from each heart, and the diameters and areas of 100 to 200 cross-sectionally oriented myocytes were measured and analyzed with ImageJ. For FLAG immunofluorescence, hearts were harvested and fixed in 4% paraformaldehyde at 4°C for 1 h. Tissues were then switched to 10% sucrose/PBS overnight followed by 18% sucrose/PBS at 4°C overnight before they were frozen, embedded, and sectioned. For immunostaining, slides were rinsed 3 times in PBS and blocked in 10% goat serum for 20 min, followed by 3 rinses in PBS. This was followed by overnight incubation with monoclonal ANTI-FLAG M2, Clone M2 (F1804, Sigma) primary antibody. The following day, slides were washed 3 times in PBS and incubated with anti-mouse or anti-rabbit secondary antibodies conjugated (1:400 dilution; Invitrogen) for 1 h. Slides were washed and mounted in anti-fade reagent.

### Glycogen content

Glycogen content was estimated from glucose released, as measured with the Glycogen Assay Kit (#MAK016, Sigma or ab65620, Abcam). Briefly, glycogen was homogenized in cold water and then boiled for 5 min as indicated by the manufacturer. Glycogen content was determined after hydrolyzation, and glucose units were analyzed colorimetrically (570 nm) with an appropriate standard curve [57].

### Plasma biochemistry measurements

Mouse serum levels analyses of glucose, triacylglycerides, and NEFAs were performed at UT Southwestern Metabolic Phenotyping Core. Serum triacylglycerides and glucose were quantified using a Vitros 250 clinical analyzer with chemical microslide technology (Ortho-Clinical Diagnosis, Raritan, NJ). NEFA levels in serum were quantified using an in vitro colorimetric method assay (Fujimilm Wako Diagnostics USA., Mountain View, CA). Total ketone bodies (acetoacetate [AcAc] + 3-hydroxybutyrate [3-HB]) were determined in plasma samples using colorimetric assays from Wako Diagnostics according to the manufacturer’s protocol (Wako: Autokit total ketone bodies R1 Set #415–73301; Autokit 3-HB R1 Set#413–73501; and Autokit 3-HB R2 Set#413–73601).

### ATP measurement in isolated mitochondria

Mitochondria were extracted from liver [58]. After isolation, mitochondrial protein concentration was determined by Bradford assay (BioRad), and 10 to 50 μg protein were incubated in 160 μl experimental buffer A (150  mM KCl, 25 mM Tris–HCl, 2 mM EDTA, 0.1% BSA fatty acid free, 10 mM K-phosphate, 0.1 mM MgCl_2_ (pH 7.4)), 1 mM substrate, and 20 μl fresh experimental buffer B (0.5 M Tris-acetate (pH 7.75); 0.8 mM luciferine, 20 mg·ml^−1^ luciferase). ATP synthesis was measured using a kinetic luminescence assay, as described in [59] with the following ATP standard curve: 10, 5, 2.5, 1.25, 0.6, 0.3, 0.15, and 0.075 mM.

### Echocardiography

Mice were anesthetized with isoflurane, and echocardiography was performed with a 30-MHz transthoracic echocardiography probe. Images were obtained with the Vevo 2100 micro-ultrasound imaging system (VisualSonics, Toronto, Canada). Short-axis, long-axis, B-mode, and 2D M-mode views were obtained as described [60]. These images were used to calculate interventricular septum, left ventricular posterior wall thicknesses, and left ventricular corrected mass; the short-axis M-mode quantification was chosen as the most representative. Cardiac function was estimated from fractional shortening values that were obtained from M-mode views by a blinded echocardiography expert. For FS measurements, a long- or short-axis view of the heart was selected to obtain an M-mode registration in a line perpendicular to the left ventricular septum and the posterior wall at the level of the mitral chordae tendineae. Normal E/A ratios were established between 1.2 and 2 for contingency analysis [61,62].

### Glucose and insulin tolerance tests

For the glucose tolerance test (GTT), adult mice were fasted overnight, and mice at PD14 were food deprived for 4 h. Mice were injected intraperitoneally (IP) with 1 g/kg of body weight of glucose, and blood glucose levels were quantified with an Ascensia Breeze 2 glucose meter at 0, 15, 30, 60, 90, and 120 min postinjection. For the insulin tolerance test (ITT), 0.75 IU/kg of insulin was given IP to fed mice, and blood glucose levels were measured at 0, 15, 30, 60, 90, and 120 min postinjection.

### Fatty acid oxidation assay

Fatty acid oxidation was assessed as described before [63,64]. Briefly, pieces of fresh liver (30 mg) or heart (30 mg) were homogenized in a Potter homogenizer (5 strokes) in cold buffer (25 mM Tris–HCl, 500 nM sucrose, 1 mM EDTA-Na_2_ (pH 7.4)) and sonicated for 10 s. Homogenates were centrifuged at 420 *g* for 10 min at 4°C. Samples (60 μl) from the homogenate supernatant were used for the assay, which started by adding 340 μl of assay mixture (500 μM palmitate/0.4 μCi [1-^14^C] palmitate per reaction). Samples were incubated for 30 min at 37°C with shaking in tubes with a Whatman filter-paper circle in the cap. The reaction was stopped by adding 200 μl of 1 M perchloric acid, and 45 μl of 1 M sodium hydroxide was added to impregnate the Whatman cap. After 1 h at room temperature, the Whatman caps (containing released CO_2_) were removed, and the radioactivity associated was measured in a scintillation counter. Tubes were centrifuged at 21,000 *g* for 10 min at 4°C, and samples (400 μl) were collected from the supernatant (containing ASMs). Radioactivity was counted in a scintillation counter.

### Protocol for lipid extraction and quantification

Hearts or livers (30 mg) were homogenized in 10 volumes of ice-cold saline buffer in a Potter homogenizer (20 strokes). Lipids were extracted from 1 mg of protein [65], separated by thin layer chromatography (TLC) and quantified as described [66]. For quantification, TLC plates were stained with a solution of 10% CuSO_4_ (w/v) in 8% H_3_PO_4_ (v/v), and an image of the plate was digitalized with GS-800 densitometer (Bio-Rad Laboratories, USA). Quantification was performed with Quantity One software (Bio-Rad Laboratories, USA). A part of the lipid extract was also dissolved in isopropanol (Scharlau Chemicals, Spain), and triglycerides were measured using a commercial kit (#TK41031 Spinreact) following manufacturer’s protocol.

### Immunoprecipitation and immunoblot analyses

For immunoblots, tissue extracts were prepared in Triton lysis buffer (20 mM Tris (pH 7.4), 1% Triton X-100, 10% glycerol, 137 mM NaCl, 2 mM EDTA, 25 mM β-glycerophosphate, 1 mM sodium orthovanadate, 1 mM phenylmethylsulfonyl fluoride, and 10 μg/ml each of aprotinin and leupeptin). Extracts (20 to 50 μg protein) and immunoprecipitates (prepared from 2 to 10 mg protein) were examined by immunoblot.

For immunoprecipitation assays, heart extracts were incubated with 4 μg of the specific antibody coupled to protein G-Sepharose beads. After incubation overnight at 4°C with agitation, the captured proteins were centrifuged at 10,000 *g*, supernatants collected, and beads washed 4 times in lysis buffer. Beads were then boiled for 5 min 95°C in 10 μl of Laemli buffer. Extracts and immunoprecipitates were examined by SDS-PAGE and blotted with antibodies to the following targets: p38γ or p38δ [44,67]; p38δ (#sc7585, Sta. Cruz), β-actin (#sc-47778, Sta. Cruz), GAPDH (#sc-25778, Sta. Cruz), vinculin (#V9131, Sigma), FLAG (#F1804, Sigma); phospho-threonine-proline (#9391S), phospho-serine-proline (#2325S), phospho-Akt (Thr308) (#2965S), Akt (#9272S), phospho-GSK3α/β (Ser21/9) (#9327S), GSK3β (#9315S), phospho-Gys (Ser641) (#3891S), phospho-ACC (Ser79) (#3661S), ACC (#3676S), hormone–sensitive lipase (HSL, #4107S), phospho-HSL (Ser660) (#4126S), Fatty acid synthase (FAS, #3189S), p-PKA C (Thr197) (#4781S), PKA C α (#4782S), phospho-AMPK (thr172) (#2535S), AMPKα (#2603S), p38γ (#2307S), phospho-p38 (T180/Y182) (#9211S), or Gys1 (#3893S) (Cell Signaling). Mitochondrial complexes were assayed using Total OXPHOS Rodent WB Antibody Cocktail (ab 110413). Secondary antibodies were purchased from Invitrogen. Immunocomplexes were detected by enhanced chemiluminescence (GE Healthcare Lifesciences).

### Cell lines and cell culture

HeLa and HEK-293T cells were cultured in DMEM supplemented with 10% heat-inactivated fetal bovine serum (FBS; Sigma), glutamine (2 mM), and penicillin/streptomycin (100 μg/ml).

### Adeno-associated virus (AAV) vector production and cell infection

AAV vector production, purification, and verification were performed as described [68]. All AAV vectors were produced by the triple transfection method, using HEK293A cells. AAV plasmids were cloned and propagated in the Stbl3 *E*. *coli* strain (Life Technologies). AAV plasmids were packaged into AAV-9 capsids with the use of the helper plasmids pAdDF6 (providing the 3 adenoviral helper genes) and pAAV2/9 (providing *rep* and *cap* viral genes), obtained from PennVector. Shuttle vectors were generated by direct cloning (GeneScript) of synthesized NheI-SalI fragments into pAcTnT or pmyog cut with the same restriction enzymes.

The AAV shuttle and helper plasmids were transfected into HEK293A cells by calcium phosphate coprecipitation. A total of 840 μg plasmid DNA (mixed in an equimolar ratio) was used per Hyperflask (Corning) seeded with 1.2 × 10^8^ cells the day before. At 72 h after transfection, cells were collected by centrifugation, and the cell pellet was resuspended in TMS (50 mM Tris–HCl, 150 mM NaCl, 2 mM MgCl_2_) on ice before digestion with DNase I and RNase A (0.1 mg/ml each; Roche) at 37°C for 60 min. Clarified supernatant containing the viral particles was obtained by iodixanol gradient centrifugation. Gradient fractions containing virus were concentrated using Amicon UltraCel columns (Millipore) and stored at −80°C. Known copy numbers (1 × 10^5^–10^8^) of the respective plasmid carrying the appropriate complementary DNA were used to construct standard curves.

### Intensity-controlled treadmill running

The exercise capacity test was modified from Kemi and colleagues [69]. Adult (12-week-old) TnTGFP or TnTp38γ/δ^act^ mice underwent a 20-min regular warm-up before they ran at fixed submaximal velocities of 0.15, 0.20, or 0.25 m/s at 25° inclination for 5 min at each velocity. The treadmill velocity was then increased by 0.03 m/s every 2 min until the mice were unable, or refused, to run further upon subtle electric stimulations located at the beginning of the treadmill platform; the total running time duration and total distance were recorded for each mouse [69].

### cDNA transfection-based experiments

Cells were plated at 60% confluence in DMEM/10% FBS at 12 to 15 h before transfection. Cells were transfected using the calcium phosphate method [70] with the cDNA expression plasmids indicated in Fig 7B and 7C and S11B and S11C Fig. The culture medium was replaced 12 h after transfection with fresh complete medium, and cells were harvested 48 h later. The plasmids used in the different experiments were pCEFL FLAG p38 gamma^D129A^ and pCMV FLAG p38 delta^F324S^ (kindly provided by David Engelberg, The Hebrew University of Jerusalem, Israel).

### In vitro kinase assay

Human GYS1 protein (1 μg) (DV-43557, DSTT, Dundee) was incubated with 1 μg of active recombinant p38γ and/or p38δ (provided by the MRC Protein Phosphorylation and Ubiquitylation Unit, Dundee, UK) in the presence of 200 μM cold ATP for 30 min. The reaction was stopped by adding SDS-containing sample buffer, and proteins were resolved by SDS-PAGE and visualized by staining with colloidal Coomassie Blue. The band containing GST-GYS1 was excised and treated with DTT to reduce disulfide bonds, and with iodoacetamide to derivatize cysteine residues. The protein was in-gel digested with trypsin, and the resulting peptides were extracted from the gel and analyzed by nanoscale-microcapillary reversed-phase liquid chromatography–tandem mass spectrometry (LC–MS/MS) [71,72].

### In vivo kinase assay

HEK293 were transfected with human FLAG-GYS1 alone or together with p38γ^act^, p38δ ^act^, or both, using the calcium phosphate transfection method [70]. Cells were harvested 48 h after transfection, and tissue extracts were prepared in Triton lysis buffer (20 mM Tris (pH 7.4), 1% Triton X-100, 10% glycerol, 137 mM NaCl, 2 mM EDTA, 25 mM β-glycerophosphate, 1 mM sodium orthovanadate, 1 mM phenylmethylsulfonyl fluoride, 10 μg/ml each aprotinin and leupeptin). Total protein concentrations were quantified with Bradford assay, and 3 mg of total protein were incubated with 2 μg of monoclonal ANTI-FLAG M2, Clone M2 (F1804, Sigma) coupled to 50 μl Protein G Dynabeads (Thermo Fisher) per experimental condition. After 2 h of incubation at 4°C with agitation, beads were washed 4 times in lysis buffer and heated for 5 min at 95°C in 20 μl sample buffer, and proteins were resolved by SDS–PAGE. For visualization, gels were stained with colloidal Coomassie Blue, and the GYS1 gel bands were excised. The band containing Flag-GYS1 was treated with DTT to reduce disulfide bonds, and with iodoacetamide to derivatize cysteine residues. The protein was in-gel digested with trypsin, and the resulting peptides were extracted and analyzed by nanoscale-microcapillary LC–MS/MS.

### Positron emission tomography (PET)

For the PET-CT acquisition, mice were anesthetized using isoflurane and oxygen. Ophthalmic gel was placed in the eyes to prevent drying. PET-CT studies were performed with a small-animal PET/CT scanner (nanoScan, Mediso, Hungary). CT study was acquired 60 min after intraperitoneal administration of 8 to 10 MBq of [^**18**^F]FDG using an X-ray beam current of 178 μA and a tube voltage of 45 kVp. After the CT scan, PET data were collected for 15 min and reconstructed using Teratomo 3D algorithm, in a 105 **×** 105 **×** 235 matrix (voxel dimensions of 0.4 mm). Regions of interest (ROIs) were delimited for the brown fat (BAT) to obtain the mean standard uptake value (SUVmean).

### Determination of glycolytic flux

The glycolytic flux was estimated by determining the rate of conversion of D-[3-^3^H]glucose into ^3^H_2_O, which, as previously validated [55], assesses the rate of ^3^H of C3-glucose interchange with water at triose-phosphate isomerase [73]. In essence, heart slices (10 to 40 mg) were preincubated for 1 h in 2 ml of a Krebs–Henseleit buffer (11 mM Na_2_HPO_4_, 122 mM NaCl, 3.1 mM KCl, 0.4 mM KH_2_PO_4_, 1.2 mM MgSO_4_, 1.3 mM CaCl_2_; pH (7.4) supplemented with 5.5 mM D-glucose at 37°C, followed by incubation in the presence of 5 μCi/ml of D-[3-^3^H]glucose in fresh Krebs–Henseleit buffer (2 ml) in glass 25-ml Erlenmeyer flasks equipped with a central well containing a tube with 0.5 ml of water. The flask atmosphere was gassed with a O_2_/CO_2_ (95/5) mixture for 20 s and stopped with a rubber cap, and the flasks were incubated in a thermostatized orbital shaker (Forma Benchtop Orbital Shaker, Model 420, Thermo Fisher) for 4 h at 37°C. ^3^H_2_O collected in the tube placed in the central well was linear with time up to at least 4 h. Incubations were finished by injecting 0.2 ml of 20% (v/v) HClO_4_ through the rubber cap, and flasks were further incubated for 72 h to allow the equilibration of ^3^H_2_O between the incubation medium and the water of the central well. The rate of glycolysis was expressed as nmol of D-[3-^3^H]glucose incorporated into ^3^H_2_O per h and per mg tissue.

### Temperature

BAT-adjacent interscapular temperature was quantified by thermographic images using a FLIR T430sc Infrared Camera (FLIR Systems, Wilsonville, OR) and analyzed through FlirIR software.

### Nuclear magnetic resonance analysis

Body, fat, and lean mass were quantified by nuclear magnetic resonance (Whole Body Composition Analyzer; EchoMRI, Houston, TX) and analyzed by ImageJ software.

### qRT-PCR

Expression of mRNA was examined by qRT-PCR using a 7900 Fast Real Time thermocycler and FAST SYBR GREEN assays (Applied Biosystems). Relative mRNA expression levels of *Pparα*, *Pppargc1α*, *Cpt1α*, *Cpt1β*, *Acox1*, *Lpl*, *Dagt2*, *Elov6*, *Hk2*, *Pepck*, *Ldha*, *Pkm2* and *Glut4*, were normalized to *Gapdh* mRNA measured in each sample.

The number of mitochondria was assessed by estimating the mitochondrial-to-chromosomal DNA ratio. The mitochondrial genes *Citb*, *Cox1*, and *Nd1* were normalized to genomic *Lpl*. Primer sequences are shown in Table 1.

**Table 1:** List of primers.

Gene		Sequence (5′–3′)
*Acox1*	FW	CCGCCACCTTCAATCCAGAG
	REV	CAAGTTCTCGATTTCTCGACG
*Cit b*	FW	TTGGGTTGTTTGATCCTGTTTCG
	REV	CTTCGCTTTCCACTTCATCTTACC
*Cox1*	FW	GTGCTGGGGCAGTGCTGGAG
	REV	TGGGGCCTGAGTAGCCCGTG
*Cpt1a*	FW	CTCCGCCTGAGCCATGAAG
	REV	CACCAGTGATGATGCCATTCT
*Cpt1b*	FW	GCACACCAGGCAGTAGCTTT
	REV	CAGGAGTTGATTCCAGACAGGTA
*Dgat2*	FW	GCGCTACTTCCGAGACTACTT
	REV	GGGCCTTATGCCAGGAAACT
*Elov6*	FW	GAGCAGAGGCGCAGAGAAC
	REV	ATGCCGACCACCAAAGATAA
*Gapdh*	FW	TGAAGCAGGCATCTGAGGG
	REV	CGAAGGTGGAAGAGTGGGA
*Glut4*	FW	TCATTGTCGGCATGGGTTT
	REV	GGCAAATAGAAGGAAGACGTAAGG
*Hk2*	FW	TGATCGCCTGCTTATTCACGG
	REV	AACCGCCTAGAAATCTCCAGA
*Ldha*	FW	TGTCTCCAGCAAAGACTACTGT
	REV	GACTGTACTTGACAATGTTGGGA
*Lpl*	FW	TTCCAGCCAGGATGCAACA
	REV	GGTCCACGTCTCCGAGTCC
*Nd1*	FW	ATATCCTAACACTCCTCGTCC
	REV	AGGGCCTTTTCGTAGTTG
*Pepck*	FW	CCATCACCTCCTGGAAGAACA
	REV	ACCCTCAATGGGTACTCCTTC
*Pkm2*	FW	CCTCGAATAGCTGCAAGTG
	REV	AAGGGGGACTACCCTCTGG
*Ppara*	FW	AGAGCCCCATCTGTCCTCTC
	REV	ACTGGTAGTCTGCAAAACCAAA
*Pppargc1a*	FW	TATGGAGTGACATAGAGTGTGCT
	REV	CCACTTCAATCCACCCAGAAAG

FW, forward primer; REV, reverse primer.

### Statistical analysis

Between-group differences were examined for statistical significance by two-tailed Student *t* test, or one-way or two-way ANOVA coupled to Tukey posttest, as indicated. Chi-squared tests were performed for categorical data. Error bars represent standard error mean (SEM).

## Supporting information

S1 Fig Mean intensity quantification of FLAG immunofluorescence in WT and TnTp38γ/δ^act^ hearts (Fig 1C) using ImageJ. Data are mean ± SEM (*n* = 4–5). ***p < 0.001 by Student *t* test. Raw data are given in S14 Fig. WT, wild-type.
(TIF)

S2 Fig Cardiac-specific p38γ/δ overexpression induce functional and geometrical changes in both male and female mice. Echocardiography analyses of hearts at PD14 from AAV-cTnT-GFP-Luc (TnTGFP; control mice) or TnTp38γ**/**δ^act^ mice (with AAV injection at PD1). Data are mean ± SEM. (*n =* 6–8). *p < 0.05, **p < 0.01, ***p < 0.001 by Student *t* test. Raw data are given in S14 Fig. AAV, adeno-associated virus; EF, ejection fraction; FS, fractional shortening; IVS;d, interventricular septum thickness in diastole; LVPW;d, left ventricle posterior wall thickness in diastole.
(TIF)

S3 Fig Overexpression of constitutively active p38γ and p38δ leads to morphological changes, fibrosis, and predisposes to a worse cardiac prognosis after MI. Mice were IV injected at PD1 with AAV-cTnT-GFP-Luc (TnTGFP) or AAV-cTnT-p38γ/δ_act_ (TnTp38γ/δ^act^) and analyzed at PD14. **(A)** Representative H&E staining of transverse heart sections (scale bar: 1 mm). **(B)** H&E staining of heart sections. Scale bar: 50 μm. **(C)** Masson’s trichrome staining quantification from heart sections (corresponds to the representative images in Fig 1K). **(D)** TnTGFP control mice or TnTp38γ/δ^act^ mice (with AAV injection at PD1) were subjected to MI at PD7 and evaluated after 4 weeks. Echocardiography measurements. Data are mean ± SEM (*n* = 4–10). **p < 0.01; ***p < 0.001 by Student *t* test. Raw data are given in S14 Fig. AAV, adeno-associated virus; FS, fractional shortening; H&E, hematoxylin and eosin; IVS;d, interventricular septum thickness in diastole; LVID;d, left ventricular internal diameter in diastole; LVPW;d, left ventricle posterior wall thickness in diastole; MI, myocardial infarction; WT, wild-type.
(TIF)

S4 Fig Cardiac-specific p38γ/δ^act^ overexpression decreases glycolysis but does not alter expression of enzymes involved in lipid metabolism or mitochondrial function. Mice were IV injected at PD1 with AAV-cTnT-GFP-Luc (TnTGFP) or AAV-cTnT-p38γ/δ_act_ (TnTp38/δ^act^) and killed at PD14. **(A)** Immunoblot expression analysis of lipid metabolic enzymes in cardiac tissue with its respective quantification. **(B)** Cardiac mitochondrial to chromosomal DNA ratio. **(C)** Immunoblot of mitochondrial complexes in heart lysates. **(D)** Cardiac mitochondrial ATP production, measured upon exposure to glutamate plus malate or free fatty acids. **(E)** qRT-PCR of enzymes involved in cardiac lipid metabolism. Data are mean ± SEM (*n =* 10–15). *p < 0.05, ***p < 0.001 by Student *t* test. Raw data are given in S14 Fig. qRT-PCR, real-time quantitative PCR.
(TIF)

S5 Fig Cardiac-specific p38γ/δ^act^ overexpression have impact on whole-body composition and lead to WAT lipolysis with no significant changes in liver metabolic enzymes. Mice were IV injected at PD1 with AAV-cTnT-GFP-Luc (TnTGFP) or AAV-cTnT-p38γ/δ_act_ (TnTp38/δ^act^) and analyzed at PD14. **(A)** Immunoblot analysis of FAS, p-AMPK, AMPK, and vinculin (load control) in hepatic lysates. Right panel, quantification. **(B)** Immunoblot analysis of PKA, p-HSL, HSL, p-AMPK, AMPK, p-ACC, ACC, p-p38, and vinculin (load control) in WAT lysates. Right panel, quantification. **(C)** Body weight. **(D)** MRI-estimated fat and lean mass. **(E)** Plasma insulin. Data are mean ± SEM. (*n* = 4–20). *p < 0.05; **p < 0.01; ***p < 0.001 by Student *t* test. Raw data are given in S14 Fig. WAT, white adipose tissue.
(TIF)

S6 Fig Immunoblot quantification of Fig 4C. Mice were IV injected at PD1 with AAV-cTnT-GFP-Luc (TnTGFP) or AAV-cTnT-p38γ/δ_act_ (TnTp38/δ^act^) and killed at PD14. Immunoblot quantification is shown for (A) heart (p-AKT/Vinculin, p38γ^act^/Vinculin and p38δ^act^/Vinculin), (B) liver (p-AKT/Vinculin), (C) muscle (p-AKT/Vinculin), and (D) WAT (p-AKT/Vinculin). Data are mean ± SEM (*n =* 3 or 4). *p < 0.05, **p < 0.01, ***p < 0.001 by ANOVA coupled to Tukey posttest. Raw data are given in S14 Fig. WAT, white adipose tissue.
(TIF)

S7 Fig Muscle-specific p38γ/δ^act^ overexpression after birth does not alter whole-body metabolism. Mice were IV injected at P1 with AAV-Myf4-EGFP (Myf4-EGFP) or AAV-Myf4-p38γ/δ_act_ (Myf4-p38γ/δ^act^) and analyzed at PD14. **(A)** Immunoblot analysis of p38γ/δ^act^ in muscle extracts. **(B)** Plasma glucose in fed or 4-h food-deprived mice. **(C)** GTT and ITT. Data are mean ± SEM (*n* = 7–9). Two-way ANOVA coupled to Tukey posttest. Raw data are given in S14 Fig. GTT, glucose tolerance test; ITT, insulin tolerance test.
(TIF)

S8 Fig Metabolic alterations precede the onsets of cardiac defects in TnTp38γ/δ^act^ mice. Mice were IV injected at PD1 with AAV-cTnT-GFP-Luc (TnTGFP) or AAV-cTnT-p38γ/δ_act_ (TnTp38γ/δ^act^), and metabolic tests were performed at PD7. **(A)** HWTL ratio. **(B)** Representative images of Masson’s trichrome staining of transverse heart sections (left) and quantification (right). Scale bar: 200 μm. **(C)** Representative images of PAS staining from heart sections (left) and quantification (right). Scale bar: 200 μm. **(D)** Cardiac glycogen content. **(E)** Left ventricle FS measured by echocardiography. **(F)** Blood glucose levels. **(G)** Plasma levels of triglycerides and NEFA. Data are mean ± SEM (*n =* 4–5). *p < 0.05; **p < 0.01 by Student *t* test. Raw data are given in S14 Fig. FS, fractional shortening; HTWL, heart weight to tibia length; NEFA, non-esterified fatty acid; PAS, periodic acid–Schiff.
(TIF)

S9 Fig Cardiac-specific overexpression of p38γ/δ^act^ decreases exercise capacity in adulthood. Mice were IV injected at PD1 with AAV-cTnT-GFP-Luc (TnTGFP) or AAV-cTnT-p38γ/δ_act_ (TnTp38γ/δ^act^) and analyzed at 12 weeks. (A) Immunoblot quantification of Fig 5A expressed as relative protein levels of exogenous active (p38γ^act^) to endogenous p38γ. (B) Exercise capacity was analyzed by measuring maximal distance and exercise duration until exhaustion. Data are mean ± SEM (*n* = 4–5). *p < 0.05 by Student *t* test. Raw data are given in S14 Fig.
(TIF)

S10 Fig Immunoblot analysis of p-PKA C (Thr197) in heart lysates of TNTGFP and TNTp38γ/δ^act^ mice. (TIF)

S11 Fig GYS1, a new phosphorylation target of p38γ and p38δ. **(A)** In an in vitro kinase assay, recombinant human GYS1 protein (2 μg) was incubated alone or in the presence of active recombinant p38γ or p38δ, or both, with 0.2 mM of cold ATP for 60 min. The table shows the GYS1 residues phosphorylated by p38γ, p38δ, or both. Data are representative of at least 3 independent experiments. **(B, C)** In an in vivo kinase assay, HEK-293 cells were transfected with p38γ, p38δ, or both, and GYS1 was immunoprecipitated to obtain the MS/MS spectra. GYS1 phosphorylated sites are indicated in red. The table shows the GYS1 residues phosphorylated by p38γ, p38δ, or both. No phosphopeptides were identified when HEK-293 cell were transfected with an empty vector without kinase. Raw data are given in S14 Fig. GYS1, glycogen synthase 1.
(TIF)

S12 Fig Cardiac-specific p38γ^act^ or p38δ^act^ overexpression alone is not sufficient to alter either whole-body metabolism or cardiac glycogen deposition. Mice were IV injected at PD1 with AAV-cTnT-GFP-Luc (TnTGFP), AAV-cTnT-p38γ_act_ (TnTp38γ^act^), or AAV-cTnT-p38δ^act^ (TnTp38δ^act^) and killed at PD14. **(A)** Immunoblot analysis of GYS1 phosphorylation at the GSK3 canonical site, S641. **(B)** Representative images of Masson’s trichrome and PAS staining on transverse heart sections. Scale bar: 200 μm. **(C)** Plasma glucose. **(D)** GTT and ITT. **(E)** Plasma NEFA and triglycerides. Data are mean ± SEM (*n =* 10) ***p <0.001 by one-way or two-way ANOVA coupled to Tukey posttest. Raw data are given in S14 Fig. GSK3, glycogen synthase kinase-3; GTT, glucose tolerance test; GYS1, glycogen synthase 1; ITT, insulin tolerance test; NEFA, non-esterified fatty acid; PAS, periodic acid–Schiff.
(TIF)

S13 Fig Maternal HFD feeding has almost no effects on cardiac and thermogenic capacity in TNT-GFP mice. CD1 females were crossed; after pregnancy confirmation by vaginal plug appearance, they were fed a HFD for the entire experiment (e.g., pregnancy and lactation). Neonates were IV injected at PD1 with AAV-cTnT-GFP-Luc (TnTGFP); during their lactation, mother remained on the same diet (e.g., ND or HFD) as during pregnancy. Pups were killed at PD14 and analyzed. (A) Percentage of mice at PD14 with normal or abnormal mitral valve flow (E/A) as an indicator of diastolic dysfunction. (B) Echocardiography measured parameters. (C) LVID,d. (D) BAT temperature chart from mice at PD14. Data are mean ± SEM (*n* = 9 or 10). **p < 0.01 by Student *t* test or chi-squared test. Raw data are given in S14 Fig. BAT, brown adipose tissue; FS, fractional shortening; HFD, high-fat diet; LVID,d, left ventricular internal diameter in diastole; LV mass, left vetricular mass; LV Vol, d, left ventricular volume in diastole; ND, normal diet.
(TIF)

S14 Fig Excel spreadsheet with raw data from all figures. (XLSX)

S15 Fig Raw western blot images. (PDF)

## Abbreviations

AAV: adeno-associated virus
ACC: acetyl-CoA carboxylase
ANP: atrial natriuretic peptide
ASM: acid-soluble metabolite
BAT: brown adipose tissue
BNP: B-type/ventricular natriuretic peptide
FBS: fetal bovine serum
FS: fractional shortening
GSD: glycogen storage disease
GSK3: glycogen synthase kinase-3
GTT: glucose tolerance test
GYS1: glycogen synthase 1
GYS2: glycogen synthase 2
H&E: hematoxylin and eosin
HF: heart failure
HFD: high-fat diet
HIF: hypoxia-inducible factor
HW/TL: heart weight to tibia length
ITT: insulin tolerance test
LC–MS/MS: liquid chromatography–tandem mass spectrometry
MI: myocardial infarction
NEFA: non-esterified fatty acid
ORO: oil red O
PAS: periodic acid–Schiff
PD: postnatal day
PET: positron emission tomography
SAPK: stress-activated protein kinase
SEM: standard error of the mean
TLC: thin layer chromatography
WAT: white adipose tissue
WGA: wheat germ agglutinin
