# Supplementary figures and images for: p38γ and p38δ regulate postnatal cardiac metabolism through glycogen synthase 1
Source: PLoS Biol. 2021 Nov 10;19(11):e3001447. doi: 10.1371/journal.pbio.3001447 (PMC8612745; doi:10.1371/journal.pbio.3001447)

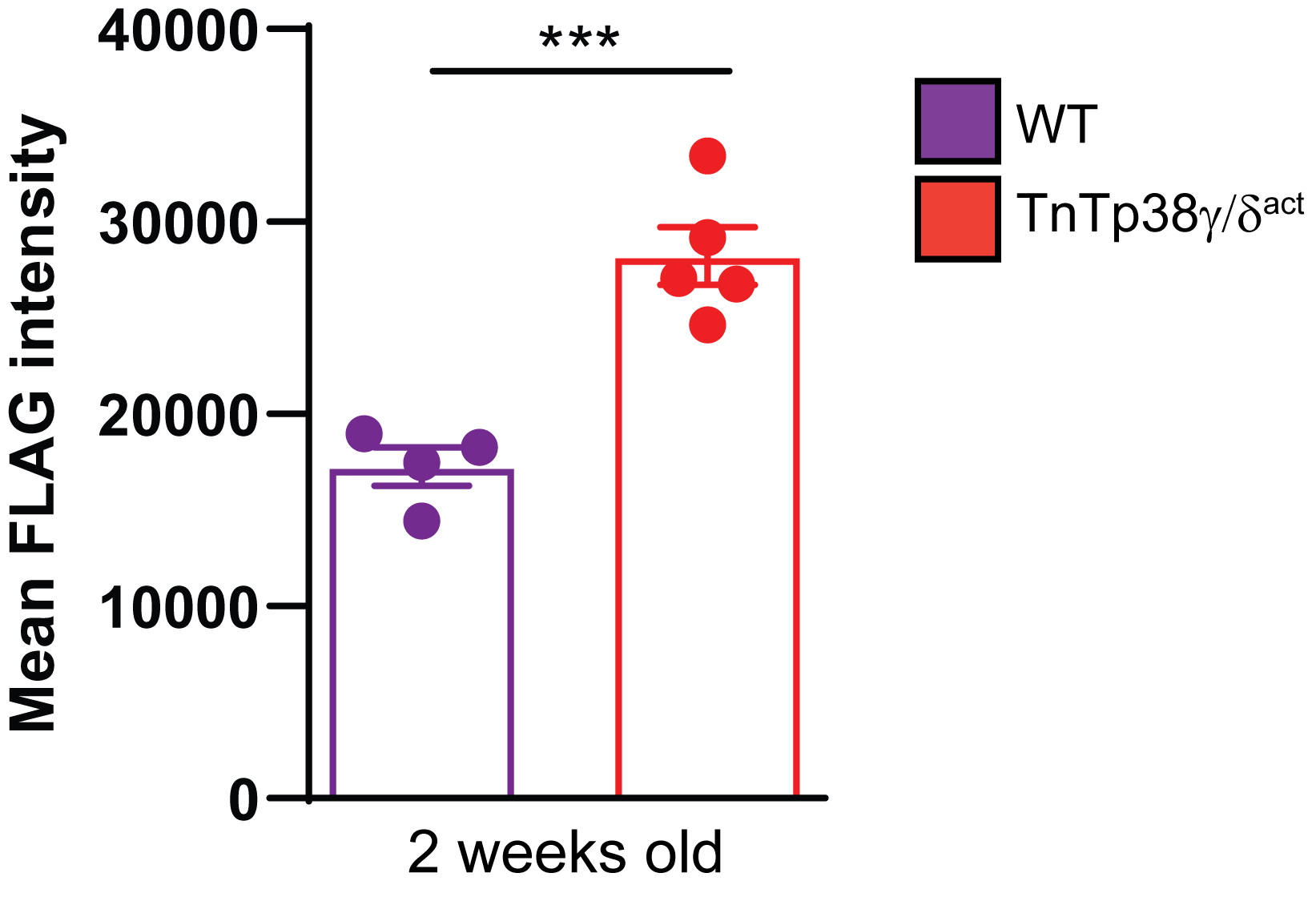

Supplement: S1 Fig — Data are mean ± SEM (n = 4–5). ***p < 0.001 by Student t test. Raw data are given in S14 Fig. WT, wild-type. (TIF) [file pbio.3001447.s001.tif]

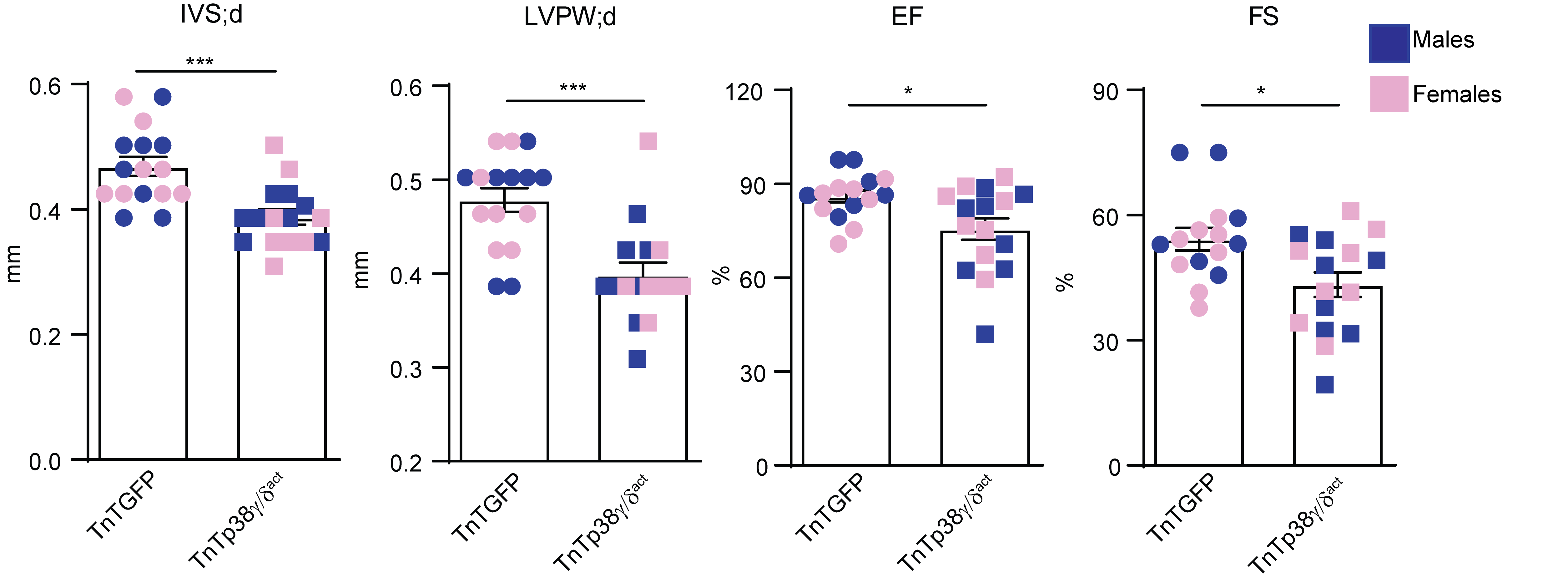

Supplement: S2 Fig — Echocardiography analyses of hearts at PD14 from AAV-cTnT-GFP-Luc (TnTGFP; control mice) or TnTp38γ/δact mice (with AAV injection at PD1). Data are mean ± SEM. (n = 6–8). *p < 0.05, **p < 0.01, ***p < 0.001 by Student t test. Raw data are given in S14 Fig. AAV, adeno-associated virus; EF, ejection fraction; FS, fractional shortening; IVS;d, interventricular septum thickness in diastole; LVPW;d, left ventricle posterior wall thickness in diastole. (TIF) [file pbio.3001447.s002.tif]

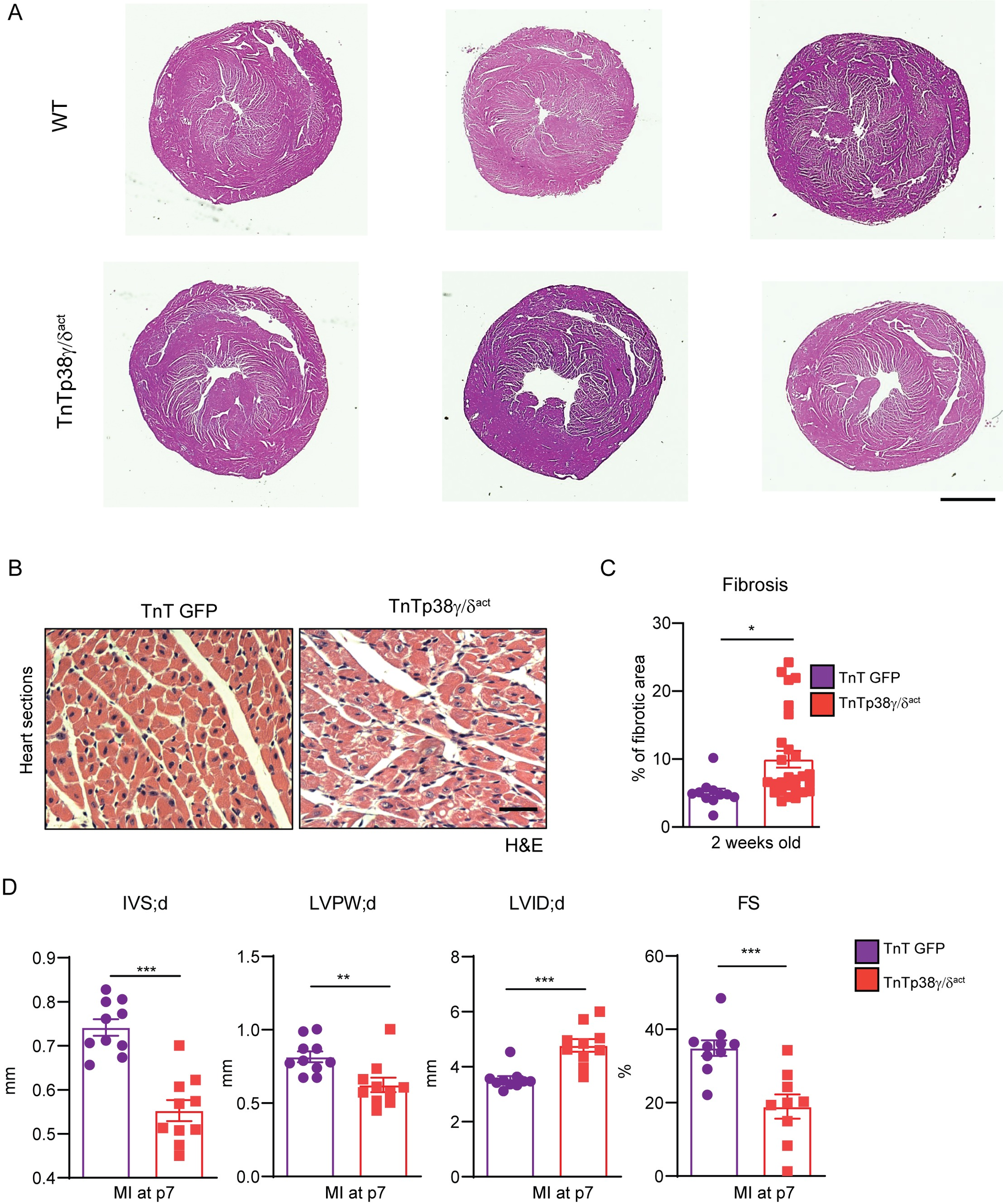

Supplement: S3 Fig — Mice were IV injected at PD1 with AAV-cTnT-GFP-Luc (TnTGFP) or AAV-cTnT-p38γ/δact (TnTp38γ/δact) and analyzed at PD14. (A) Representative H&E staining of transverse heart sections (scale bar: 1 mm). (B) H&E staining of heart sections. Scale bar: 50 μm. (C) Masson’s trichrome staining quantification from heart sections (corresponds to the representative images in Fig 1K). (D) TnTGFP control mice or TnTp38γ/δact mice (with AAV injection at PD1) were subjected to MI at PD7 and evaluated after 4 weeks. Echocardiography measurements. Data are mean ± SEM (n = 4–10). **p < 0.01; ***p < 0.001 by Student t test. Raw data are given in S14 Fig. AAV, adeno-associated virus; FS, fractional shortening; H&E, hematoxylin and eosin; IVS;d, interventricular septum thickness in diastole; LVID;d, left ventricular internal diameter in diastole; LVPW;d, left ventricle posterior wall thickness in diastole; MI, myocardial infarction; WT, wild-type. (TIF) [file pbio.3001447.s003.tif]

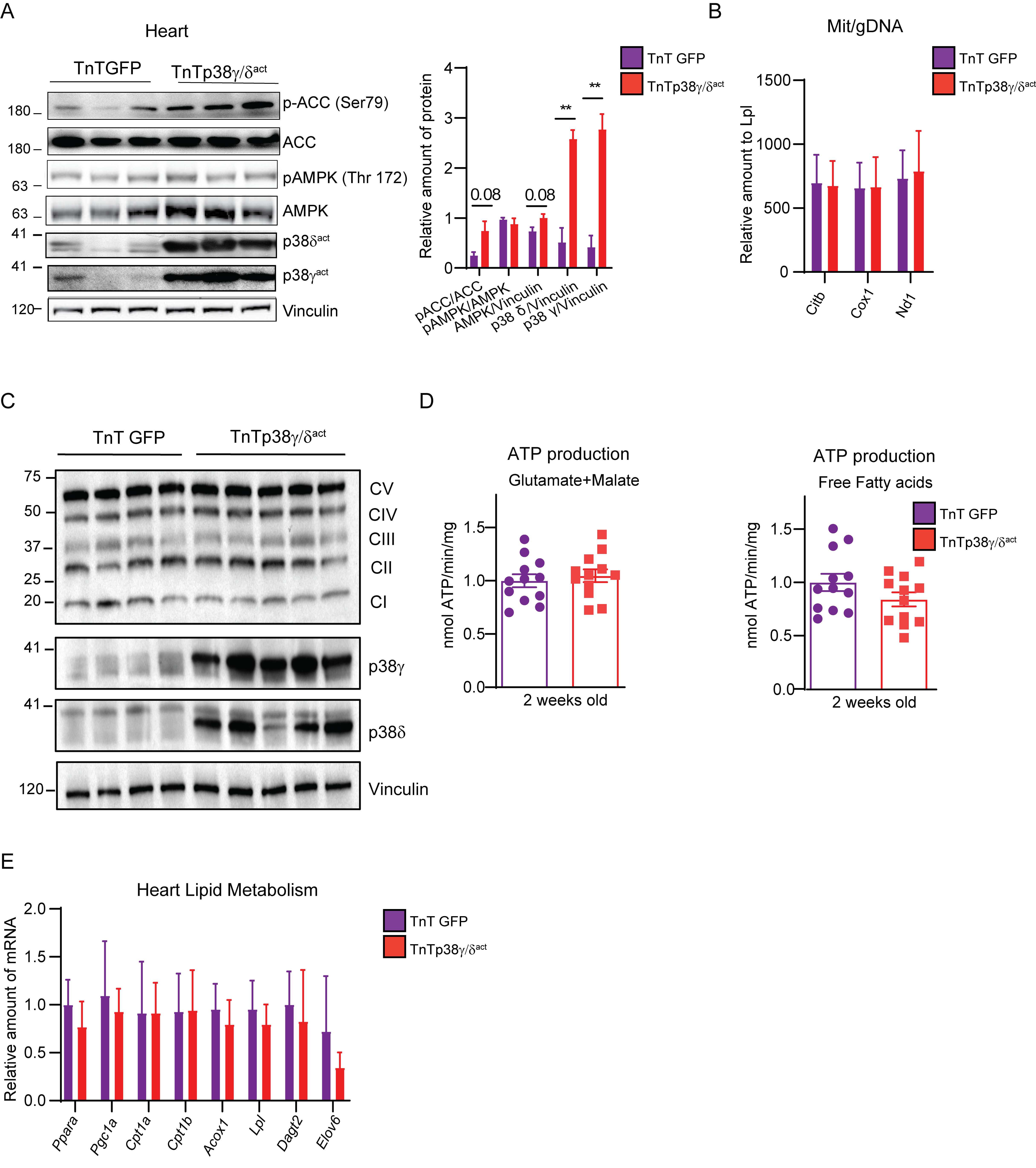

Supplement: S4 Fig — Mice were IV injected at PD1 with AAV-cTnT-GFP-Luc (TnTGFP) or AAV-cTnT-p38γ/δact (TnTp38/δact) and killed at PD14. (A) Immunoblot expression analysis of lipid metabolic enzymes in cardiac tissue with its respective quantification. (B) Cardiac mitochondrial to chromosomal DNA ratio. (C) Immunoblot of mitochondrial complexes in heart lysates. (D) Cardiac mitochondrial ATP production, measured upon exposure to glutamate plus malate or free fatty acids. (E) qRT-PCR of enzymes involved in cardiac lipid metabolism. Data are mean ± SEM (n = 10–15). *p < 0.05, ***p < 0.001 by Student t test. Raw data are given in S14 Fig. qRT-PCR, real-time quantitative PCR. (TIF) [file pbio.3001447.s004.tif]

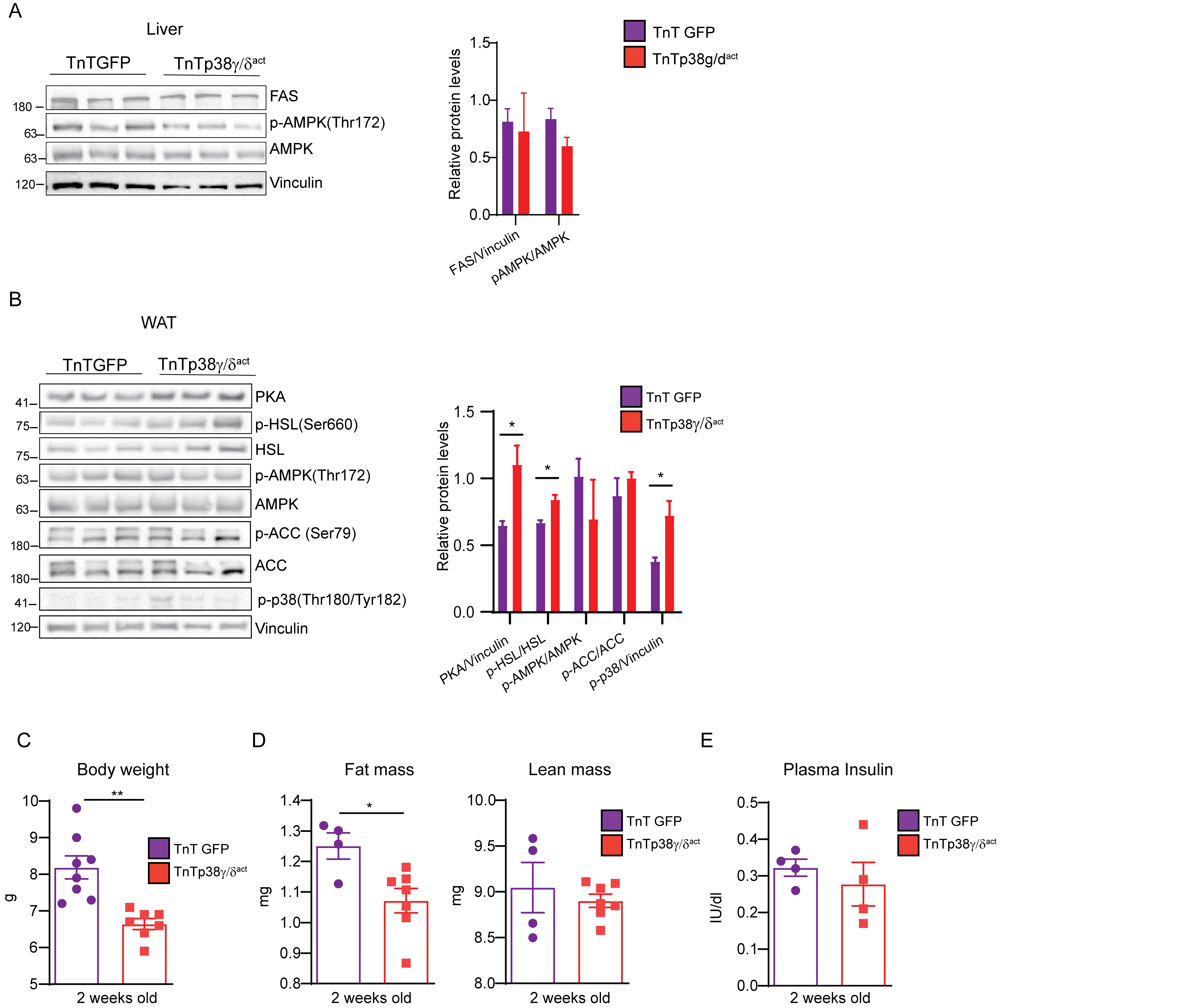

Supplement: S5 Fig — Mice were IV injected at PD1 with AAV-cTnT-GFP-Luc (TnTGFP) or AAV-cTnT-p38γ/δact (TnTp38/δact) and analyzed at PD14. (A) Immunoblot analysis of FAS, p-AMPK, AMPK, and vinculin (load control) in hepatic lysates. Right panel, quantification. (B) Immunoblot analysis of PKA, p-HSL, HSL, p-AMPK, AMPK, p-ACC, ACC, p-p38, and vinculin (load control) in WAT lysates. Right panel, quantification. (C) Body weight. (D) MRI-estimated fat and lean mass. (E) Plasma insulin. Data are mean ± SEM. (n = 4–20). *p < 0.05; **p < 0.01; ***p < 0.001 by Student t test. Raw data are given in S14 Fig. WAT, white adipose tissue. (TIF) [file pbio.3001447.s005.tif]

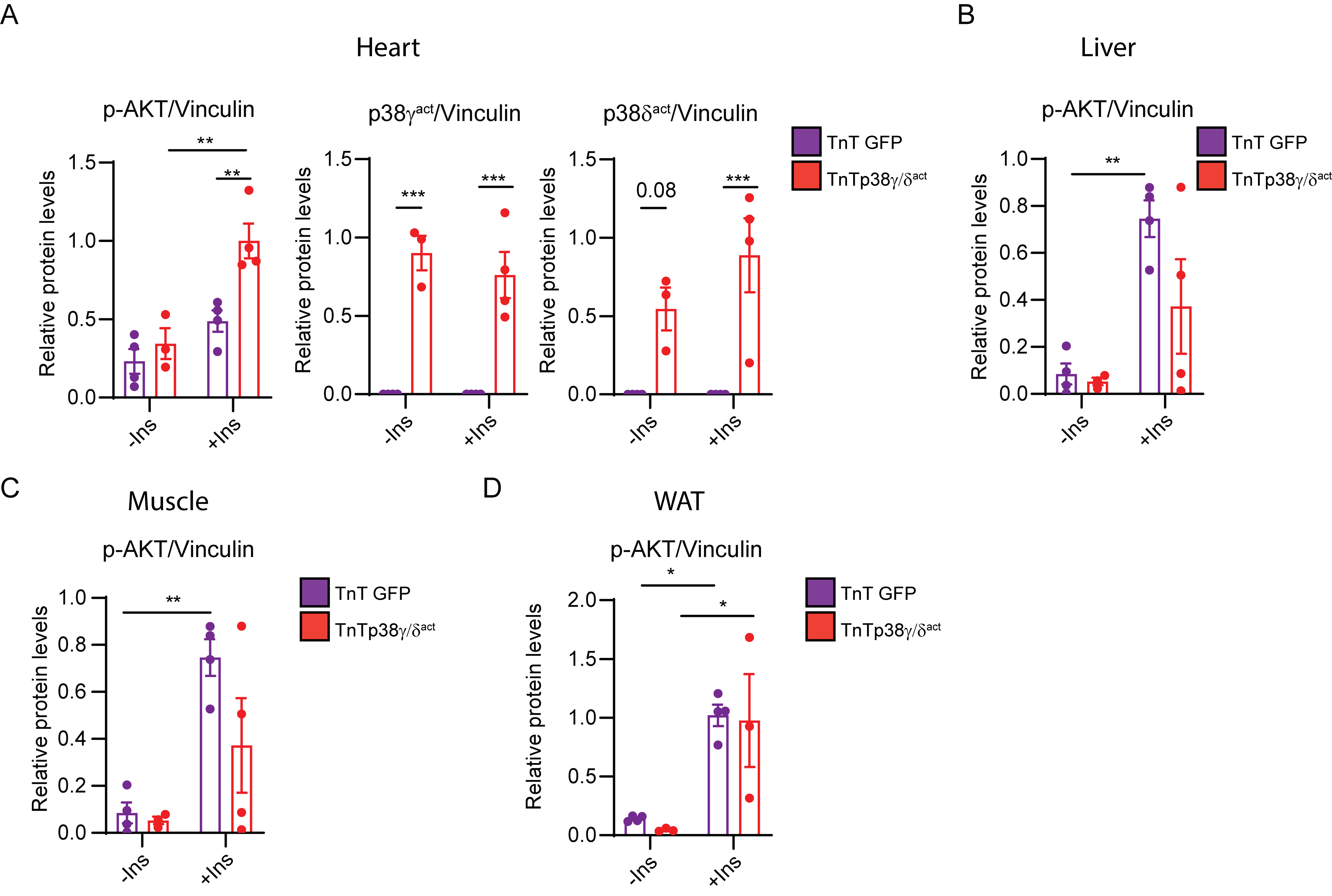

Supplement: S6 Fig — Mice were IV injected at PD1 with AAV-cTnT-GFP-Luc (TnTGFP) or AAV-cTnT-p38γ/δact (TnTp38/δact) and killed at PD14. Immunoblot quantification is shown for (A) heart (p-AKT/Vinculin, p38γact/Vinculin and p38δact/Vinculin), (B) liver (p-AKT/Vinculin), (C) muscle (p-AKT/Vinculin), and (D) WAT (p-AKT/Vinculin). Data are mean ± SEM (n = 3 or 4). *p < 0.05, **p < 0.01, ***p < 0.001 by ANOVA coupled to Tukey posttest. Raw data are given in S14 Fig. WAT, white adipose tissue. (TIF) [file pbio.3001447.s006.tif]

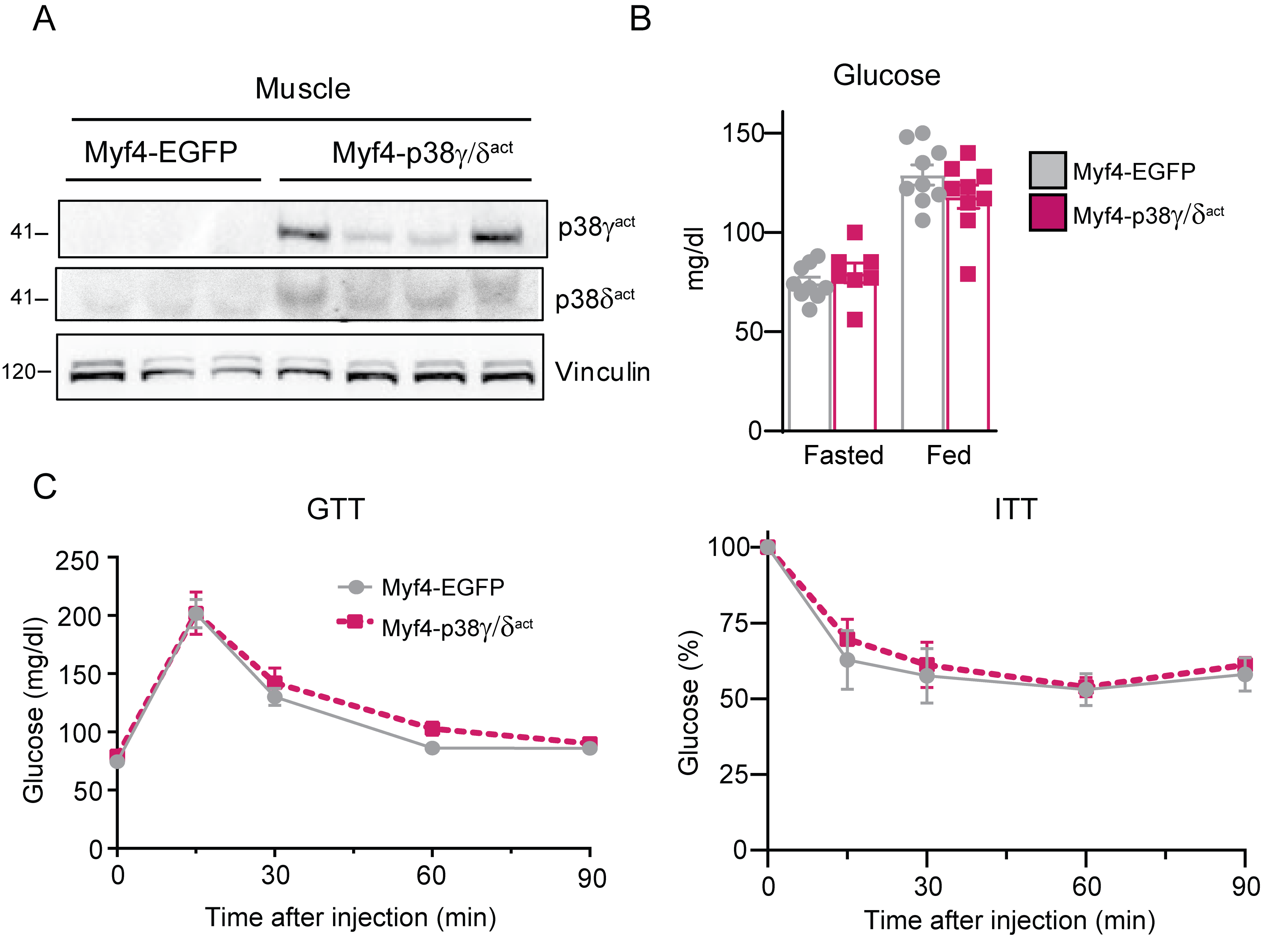

Supplement: S7 Fig — Mice were IV injected at P1 with AAV-Myf4-EGFP (Myf4-EGFP) or AAV-Myf4-p38γ/δact (Myf4-p38γ/δact) and analyzed at PD14. (A) Immunoblot analysis of p38γ/δact in muscle extracts. (B) Plasma glucose in fed or 4-h food-deprived mice. (C) GTT and ITT. Data are mean ± SEM (n = 7–9). Two-way ANOVA coupled to Tukey posttest. Raw data are given in S14 Fig. GTT, glucose tolerance test; ITT, insulin tolerance test. (TIF) [file pbio.3001447.s007.tif]

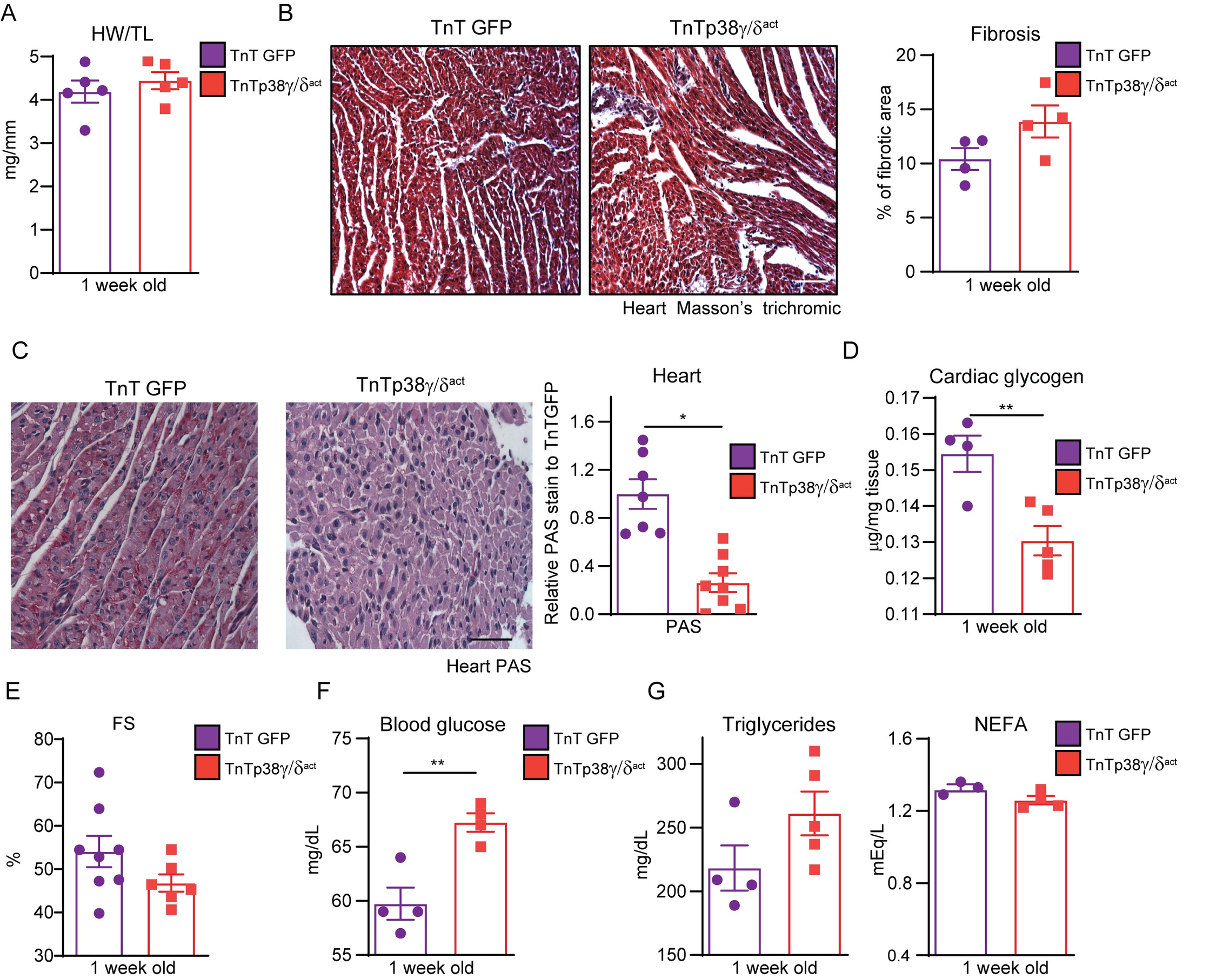

Supplement: S8 Fig — Mice were IV injected at PD1 with AAV-cTnT-GFP-Luc (TnTGFP) or AAV-cTnT-p38γ/δact (TnTp38γ/δact), and metabolic tests were performed at PD7. (A) HWTL ratio. (B) Representative images of Masson’s trichrome staining of transverse heart sections (left) and quantification (right). Scale bar: 200 μm. (C) Representative images of PAS staining from heart sections (left) and quantification (right). Scale bar: 200 μm. (D) Cardiac glycogen content. (E) Left ventricle FS measured by echocardiography. (F) Blood glucose levels. (G) Plasma levels of triglycerides and NEFA. Data are mean ± SEM (n = 4–5). *p < 0.05; **p < 0.01 by Student t test. Raw data are given in S14 Fig. FS, fractional shortening; HTWL, heart weight to tibia length; NEFA, non-esterified fatty acid; PAS, periodic acid–Schiff. (TIF) [file pbio.3001447.s008.tif]

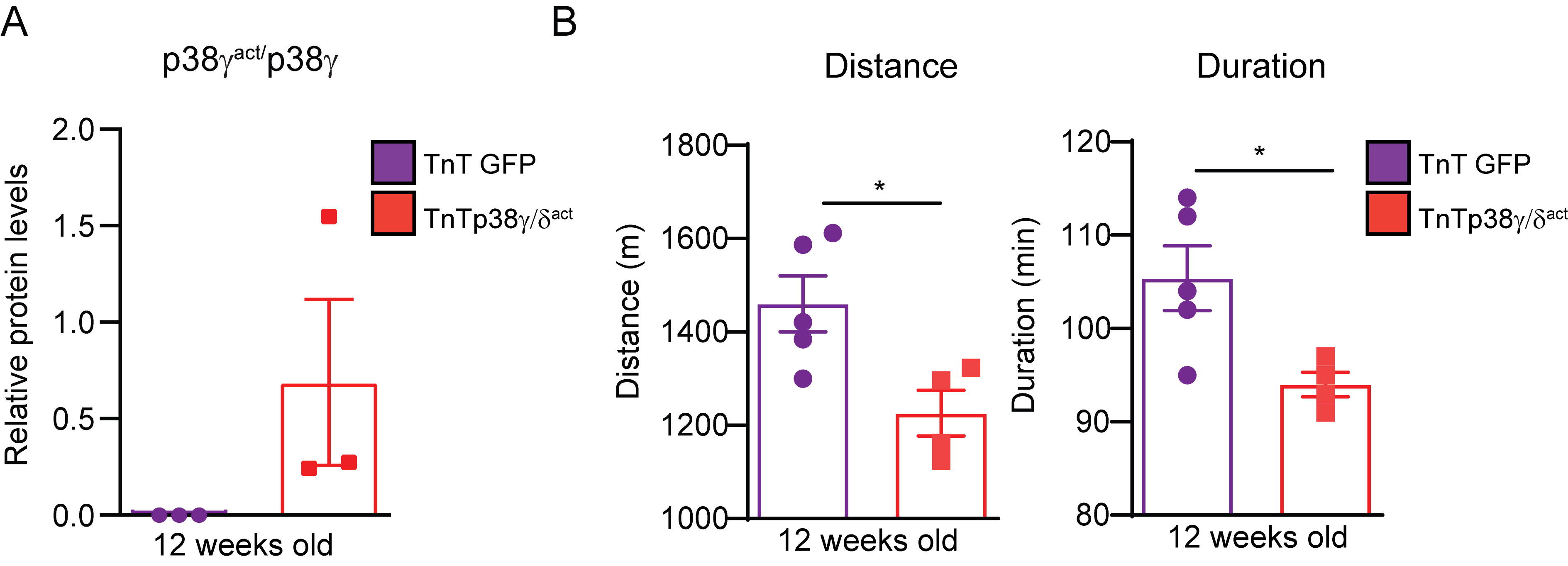

Supplement: S9 Fig — Mice were IV injected at PD1 with AAV-cTnT-GFP-Luc (TnTGFP) or AAV-cTnT-p38γ/δact (TnTp38γ/δact) and analyzed at 12 weeks. (A) Immunoblot quantification of Fig 5A expressed as relative protein levels of exogenous active (p38γact) to endogenous p38γ. (B) Exercise capacity was analyzed by measuring maximal distance and exercise duration until exhaustion. Data are mean ± SEM (n = 4–5). *p < 0.05 by Student t test. Raw data are given in S14 Fig. (TIF) [file pbio.3001447.s009.tif]

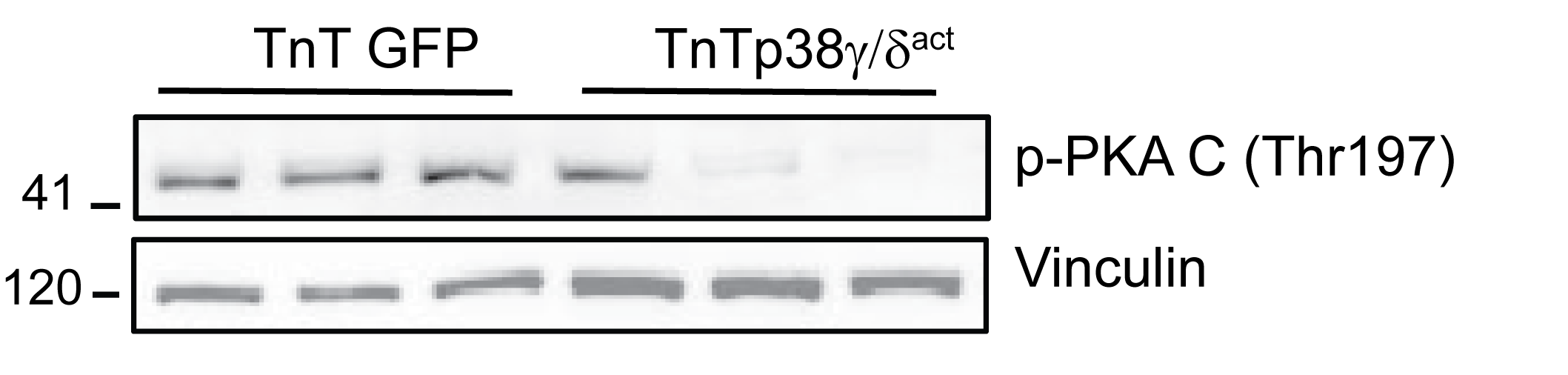

Supplement: S10 Fig — (TIF) [file pbio.3001447.s010.tif]

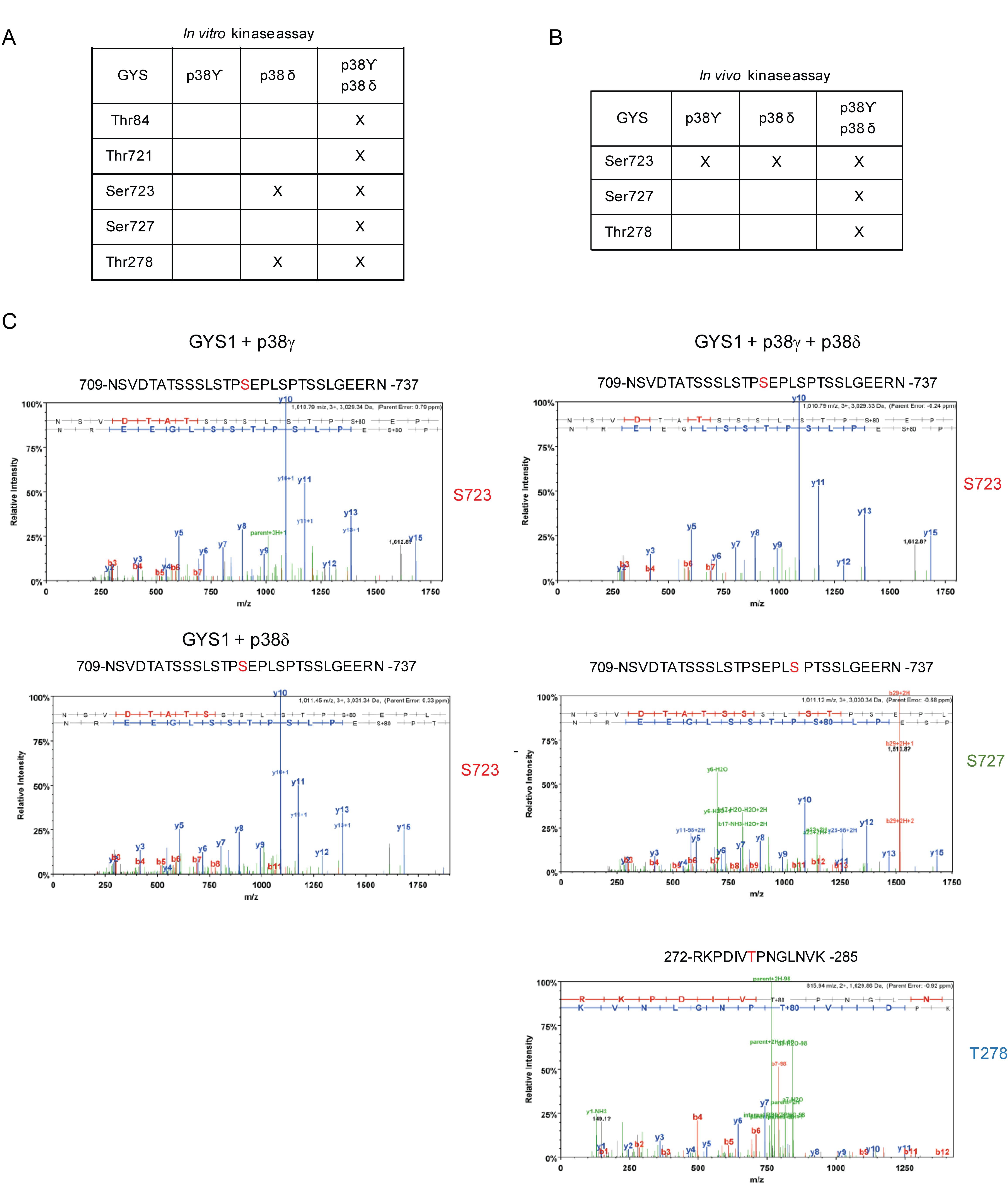

Supplement: S11 Fig — (A) In an in vitro kinase assay, recombinant human GYS1 protein (2 μg) was incubated alone or in the presence of active recombinant p38γ or p38δ, or both, with 0.2 mM of cold ATP for 60 min. The table shows the GYS1 residues phosphorylated by p38γ, p38δ, or both. Data are representative of at least 3 independent experiments. (B, C) In an in vivo kinase assay, HEK-293 cells were transfected with p38γ, p38δ, or both, and GYS1 was immunoprecipitated to obtain the MS/MS spectra. GYS1 phosphorylated sites are indicated in red. The table shows the GYS1 residues phosphorylated by p38γ, p38δ, or both. No phosphopeptides were identified when HEK-293 cell were transfected with an empty vector without kinase. Raw data are given in S14 Fig. GYS1, glycogen synthase 1. (TIF) [file pbio.3001447.s011.tif]

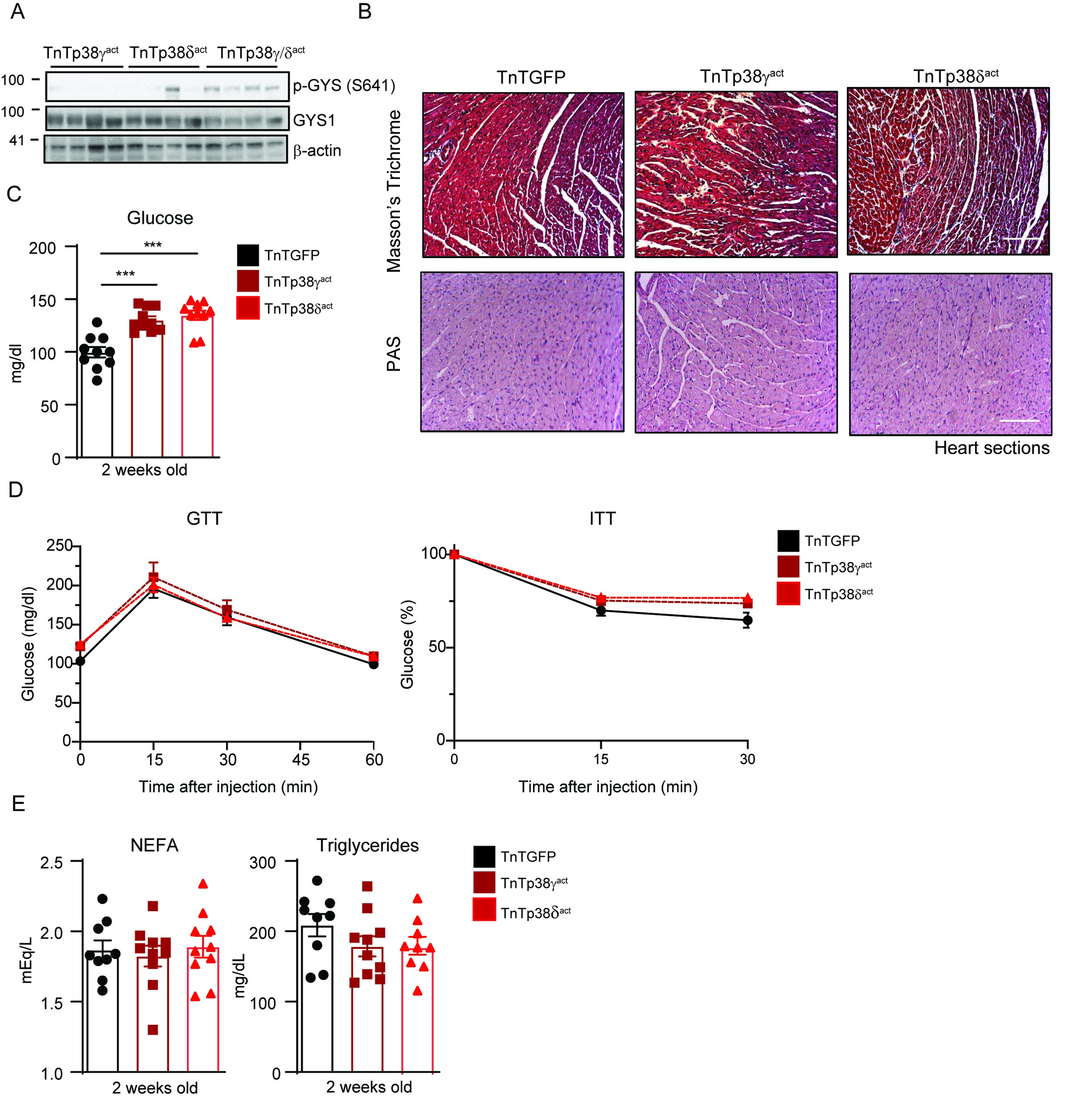

Supplement: S12 Fig — Mice were IV injected at PD1 with AAV-cTnT-GFP-Luc (TnTGFP), AAV-cTnT-p38γact (TnTp38γact), or AAV-cTnT-p38δact (TnTp38δact) and killed at PD14. (A) Immunoblot analysis of GYS1 phosphorylation at the GSK3 canonical site, S641. (B) Representative images of Masson’s trichrome and PAS staining on transverse heart sections. Scale bar: 200 μm. (C) Plasma glucose. (D) GTT and ITT. (E) Plasma NEFA and triglycerides. Data are mean ± SEM (n = 10) ***p <0.001 by one-way or two-way ANOVA coupled to Tukey posttest. Raw data are given in S14 Fig. GSK3, glycogen synthase kinase-3; GTT, glucose tolerance test; GYS1, glycogen synthase 1; ITT, insulin tolerance test; NEFA, non-esterified fatty acid; PAS, periodic acid–Schiff. (TIF) [file pbio.3001447.s012.tif]

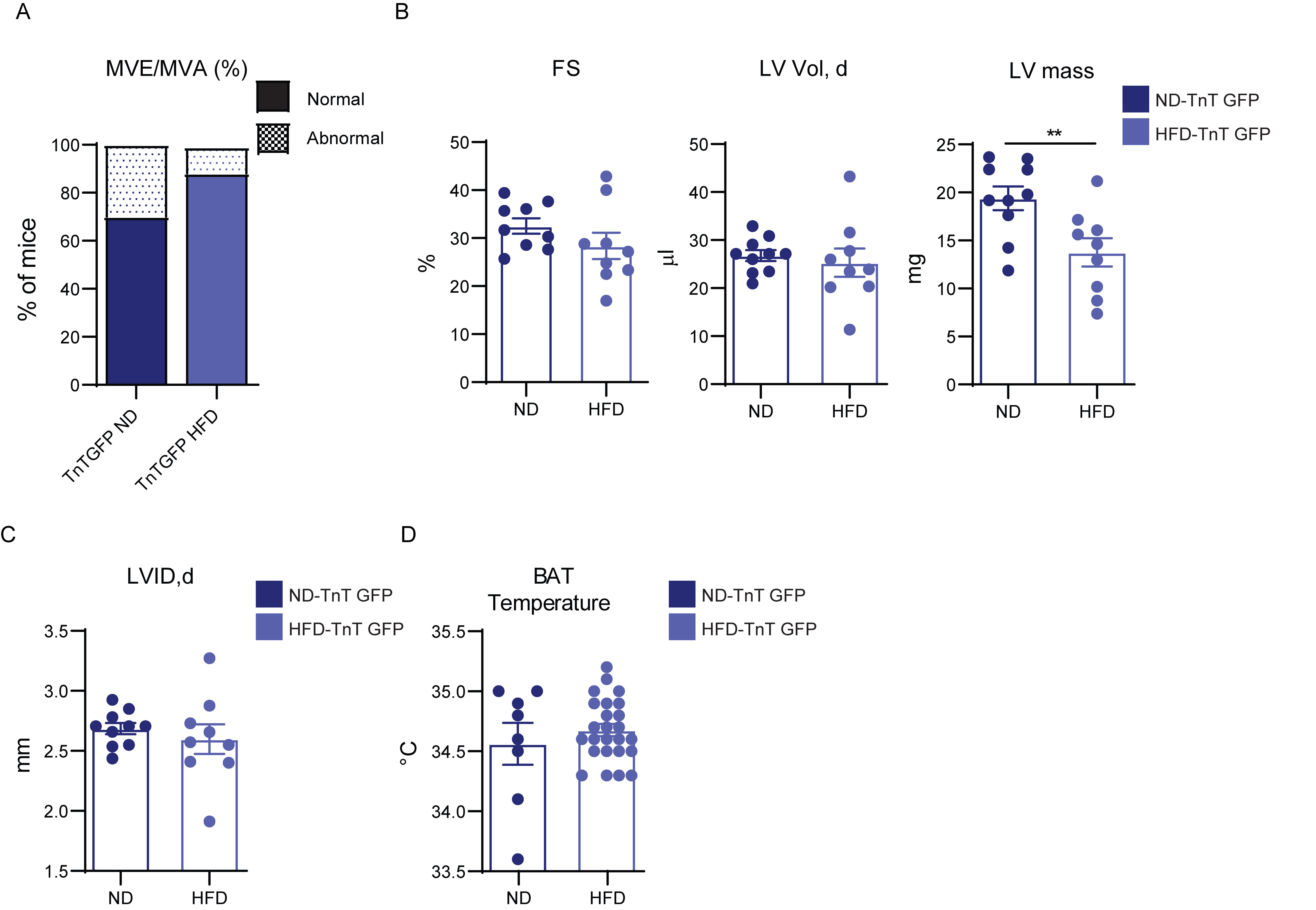

Supplement: S13 Fig — CD1 females were crossed; after pregnancy confirmation by vaginal plug appearance, they were fed a HFD for the entire experiment (e.g., pregnancy and lactation). Neonates were IV injected at PD1 with AAV-cTnT-GFP-Luc (TnTGFP); during their lactation, mother remained on the same diet (e.g., ND or HFD) as during pregnancy. Pups were killed at PD14 and analyzed. (A) Percentage of mice at PD14 with normal or abnormal mitral valve flow (E/A) as an indicator of diastolic dysfunction. (B) Echocardiography measured parameters. (C) LVID,d. (D) BAT temperature chart from mice at PD14. Data are mean ± SEM (n = 9 or 10). **p < 0.01 by Student t test or chi-squared test. Raw data are given in S14 Fig. BAT, brown adipose tissue; FS, fractional shortening; HFD, high-fat diet; LVID,d, left ventricular internal diameter in diastole; LV mass, left vetricular mass; LV Vol, d, left ventricular volume in diastole; ND, normal diet. (TIF) [file pbio.3001447.s013.tif]
