# Supplementary material for: p38γ and p38δ regulate postnatal cardiac metabolism through glycogen synthase 1
Source: PLoS Biol. 2021 Nov 10;19(11):e3001447. doi: 10.1371/journal.pbio.3001447 (PMC8612745; doi:10.1371/journal.pbio.3001447)
Supplement: S15 Fig — (PDF) [file pbio.3001447.s015.pdf]

Figure 1A

p-p38δ
p-p38γ

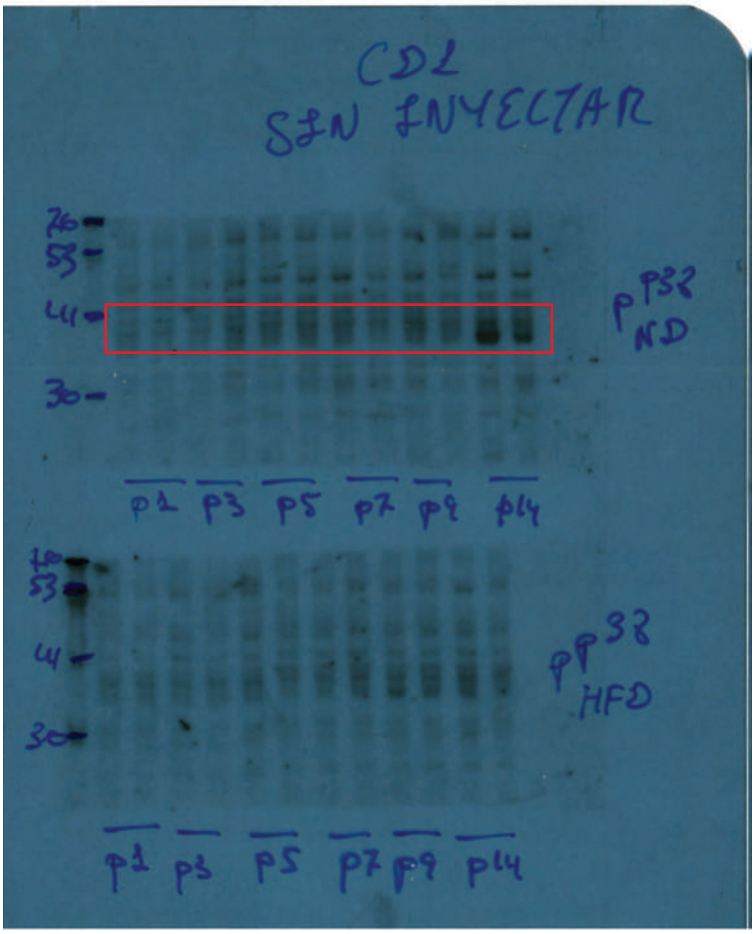

p38γ

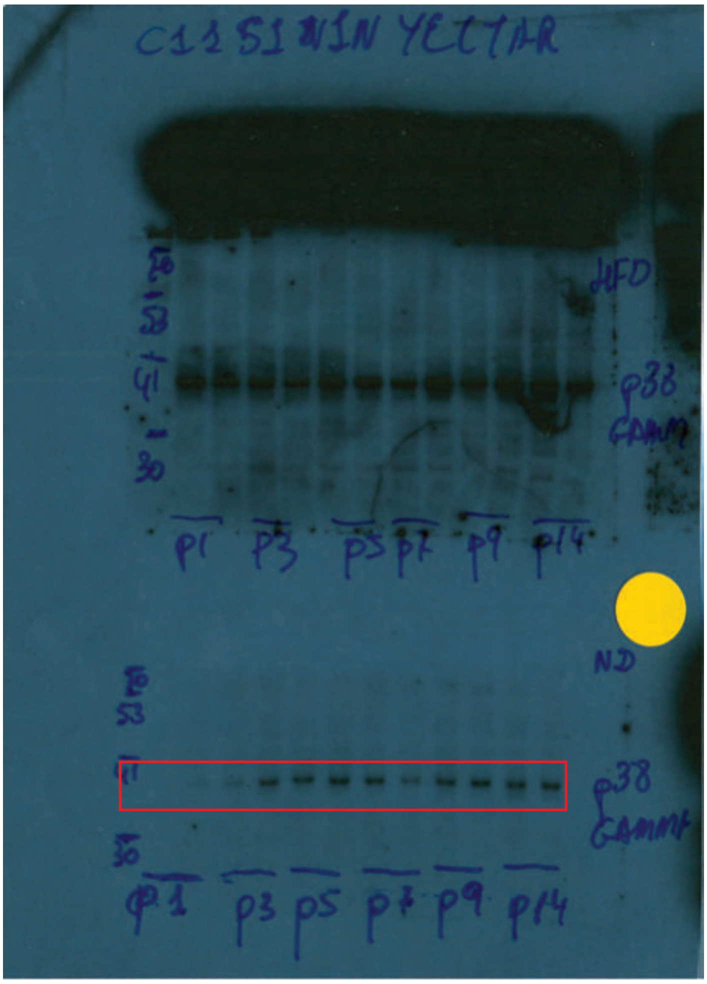

Vinculin

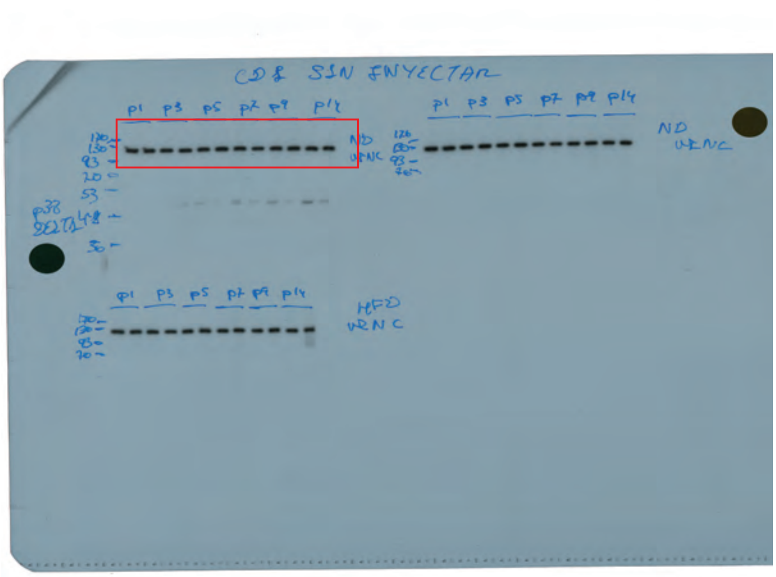

p38δ

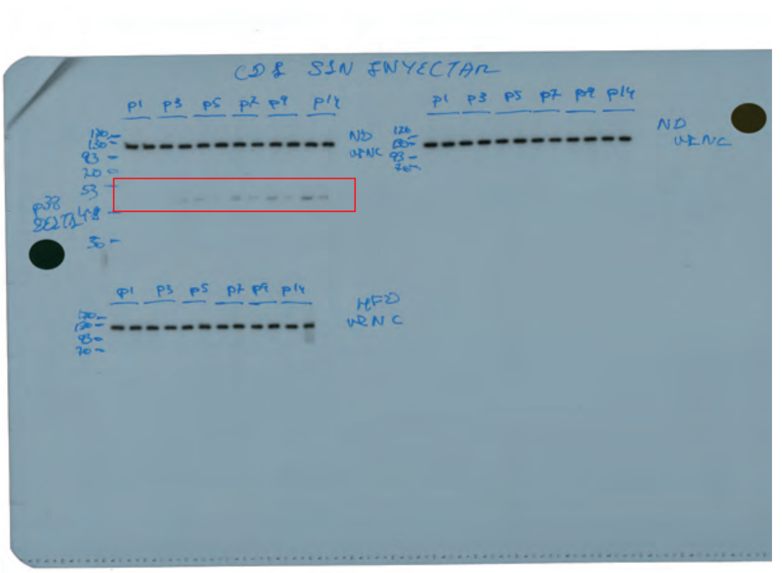

Chemiluminescence Western Blotting
X ray film detection

Figure 1B

Anti-Flag

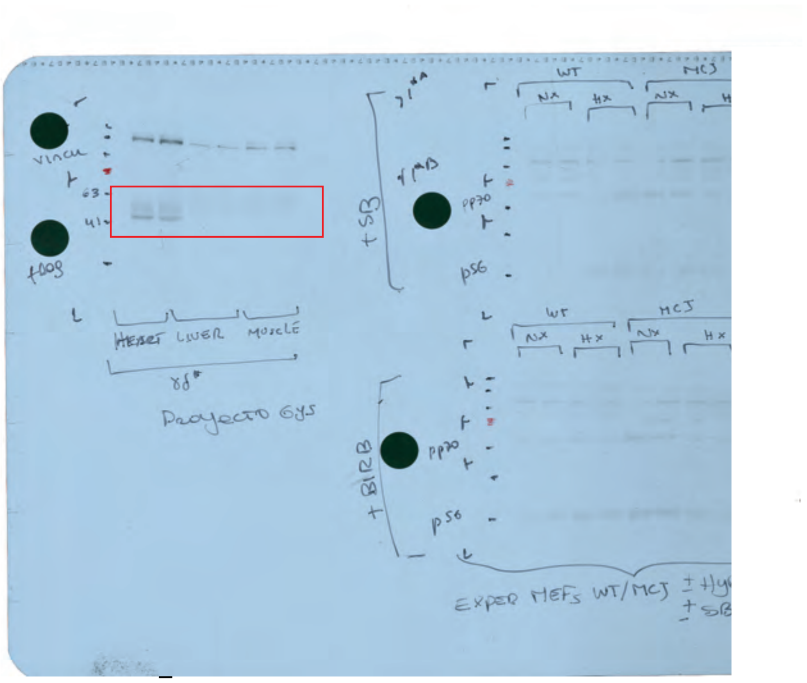

Vinculin

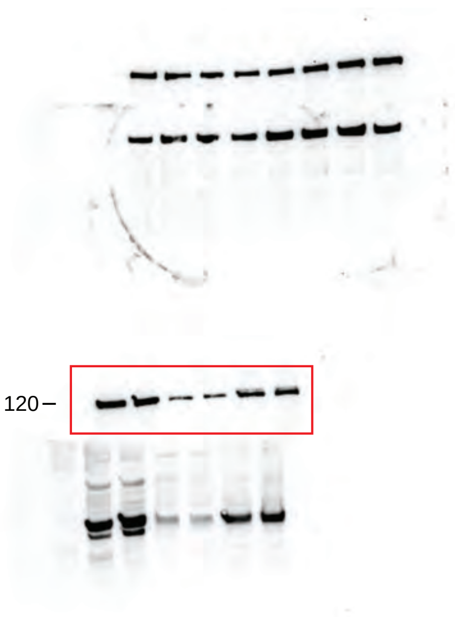

Chemiluminescence Western Blotting
X ray film detection

Chemiluminescence Western Blotting
IBright 1500 detection

Figure 4C

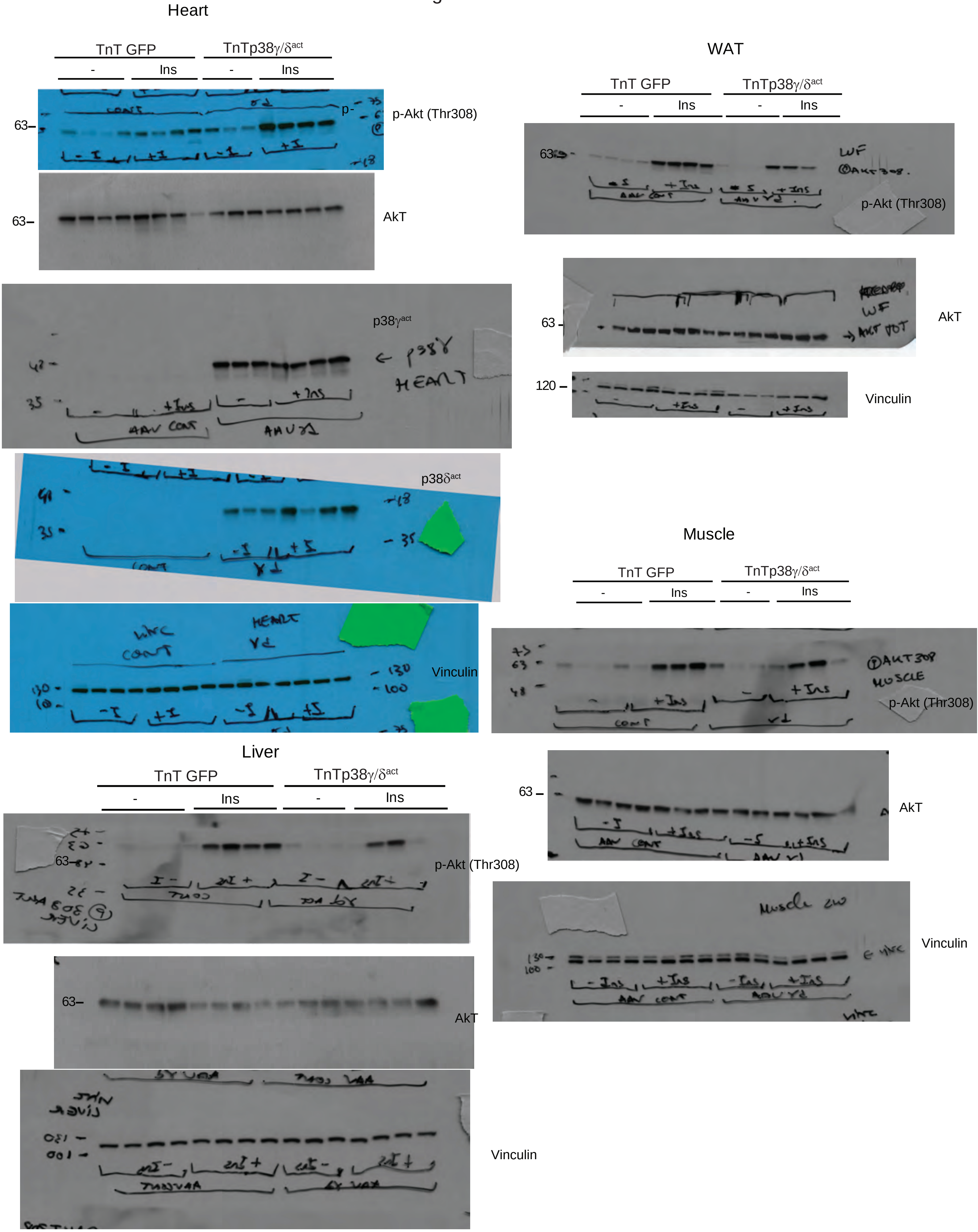

Chemiluminescence Western Blotting
X ray film detection

Figure 5A

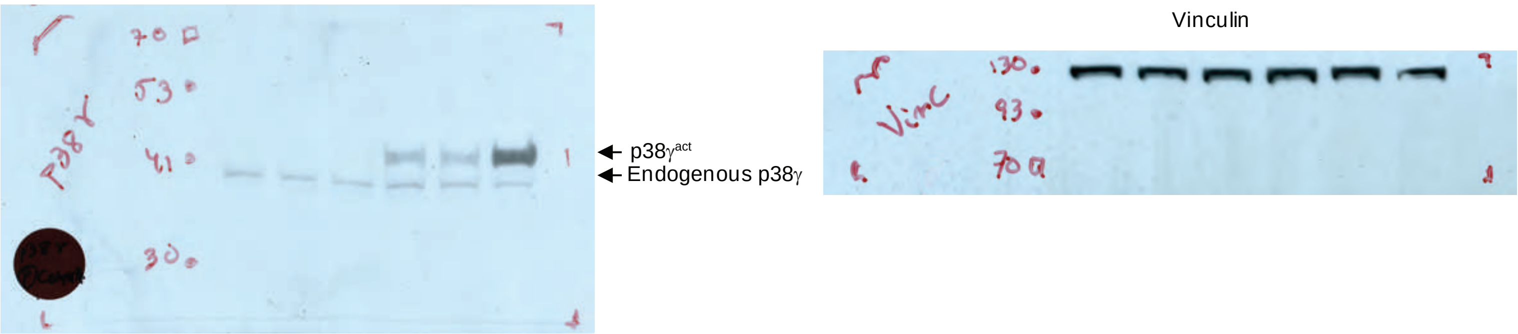

Chemiluminescence Western Blotting
X ray film detection

Figure 6A

p38γ

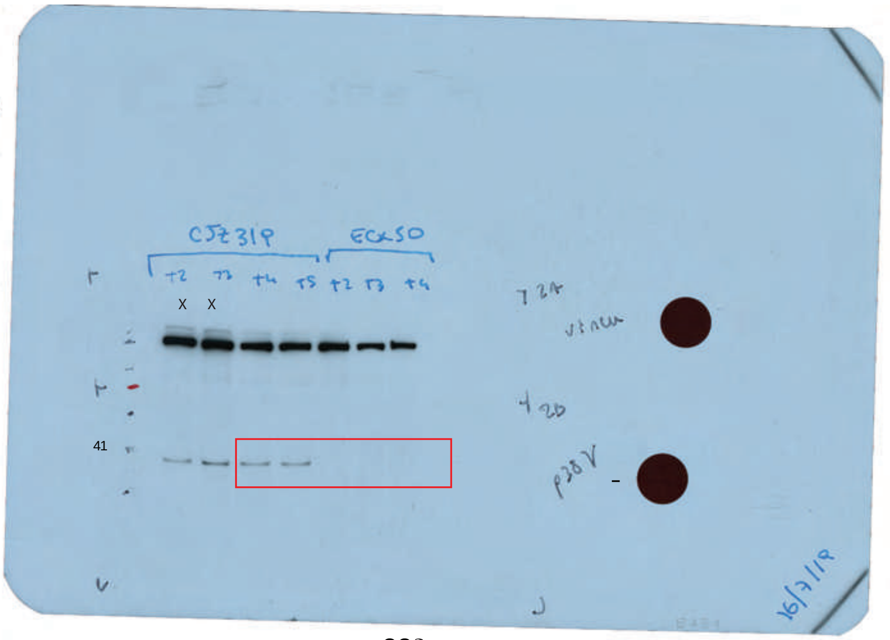

Chemiluminescence Western Blotting
X ray film detection

p38δ

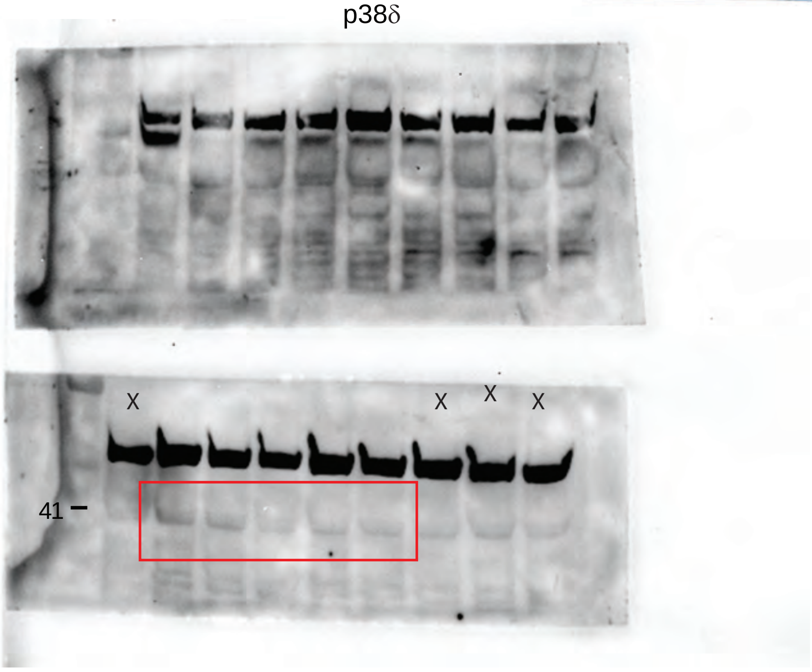

Chemiluminescence Western Blotting
IBright 1500 detection

Vinculin

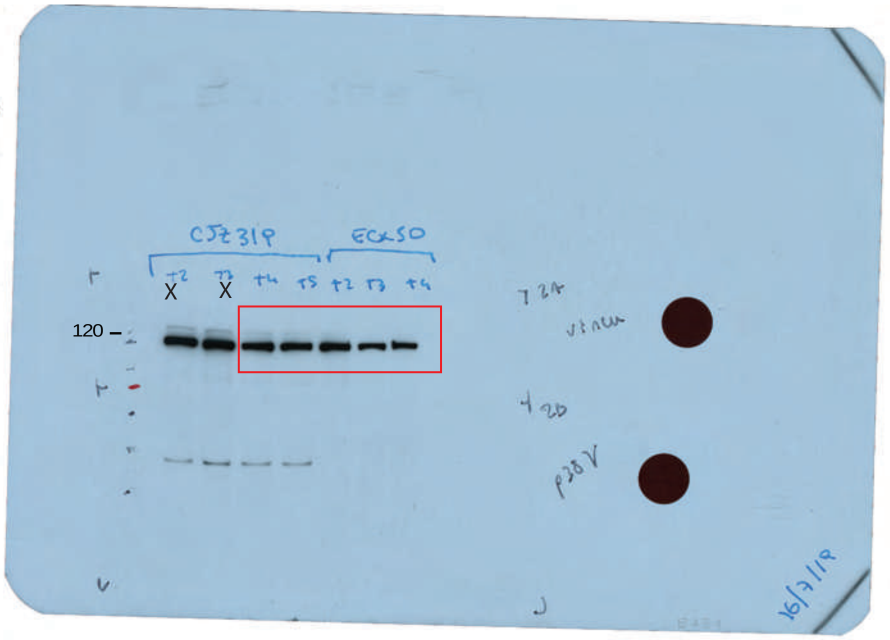

Chemiluminescence Western Blotting
X ray film detection

Figure 7A

p-GYS (S641)

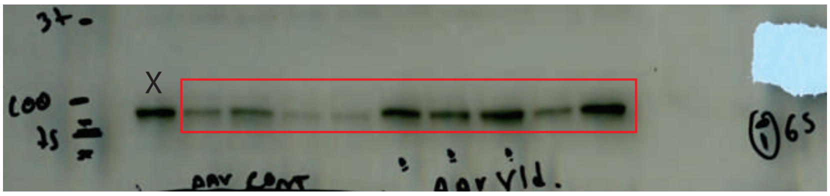

GYS1

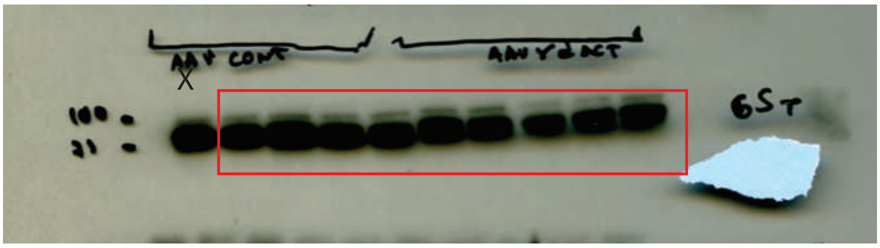

p-GSK3α/β (S21/9)

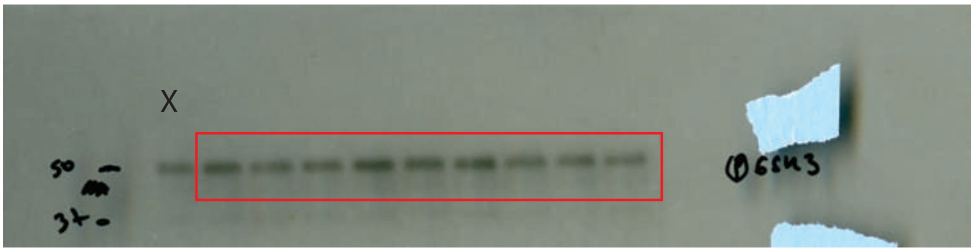

GSK3β

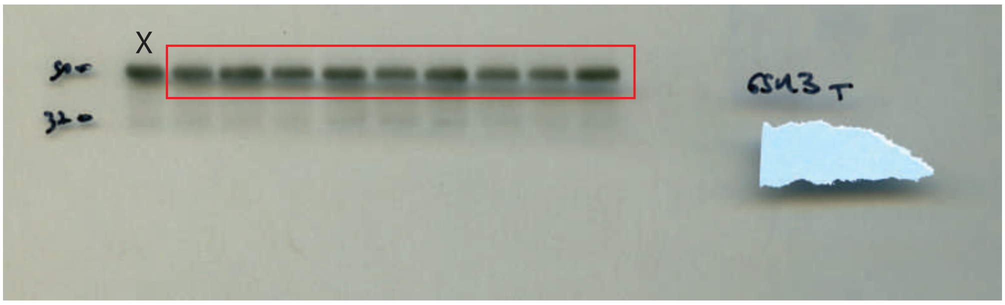

p-Akt(Thr 308)

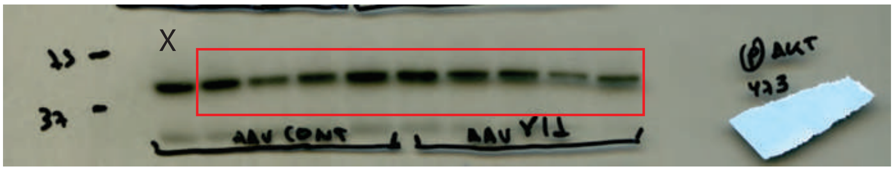

Akt

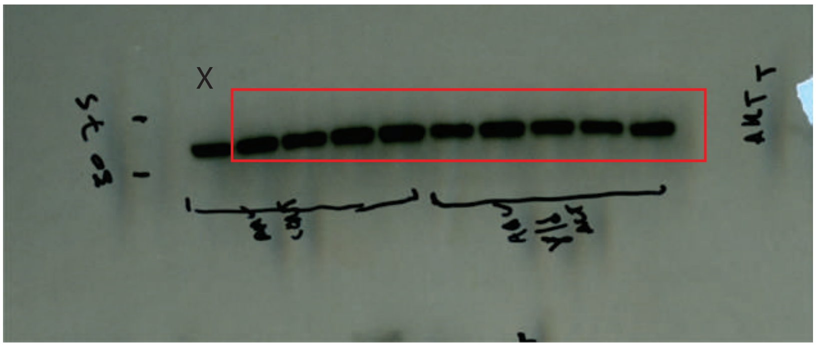

Vinculin

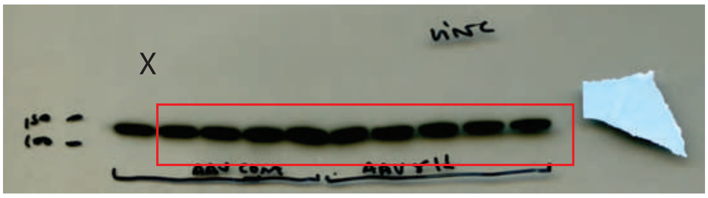

Chemiluminescence Western Blotting
X ray film detection

Figure 7D

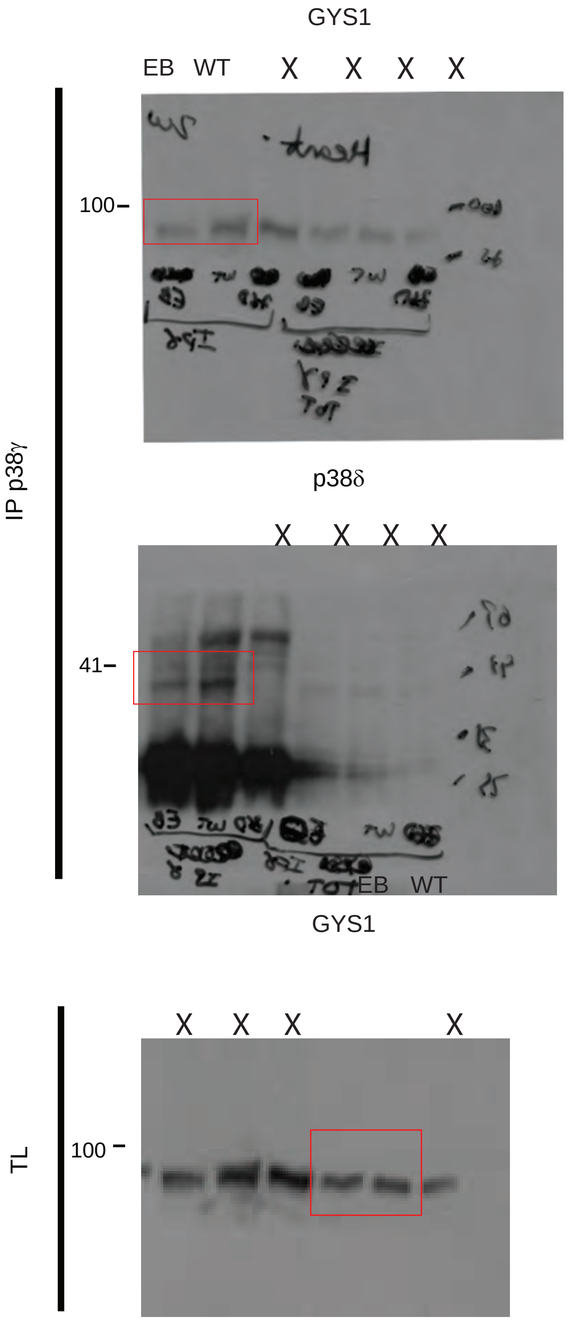

The GYS1 blots are exactly the same gels with different exposure time as the IP and the Total lysate (TL) were charged next to each other

Chemiluminescence Western Blotting
X ray film detection

Figure 7C

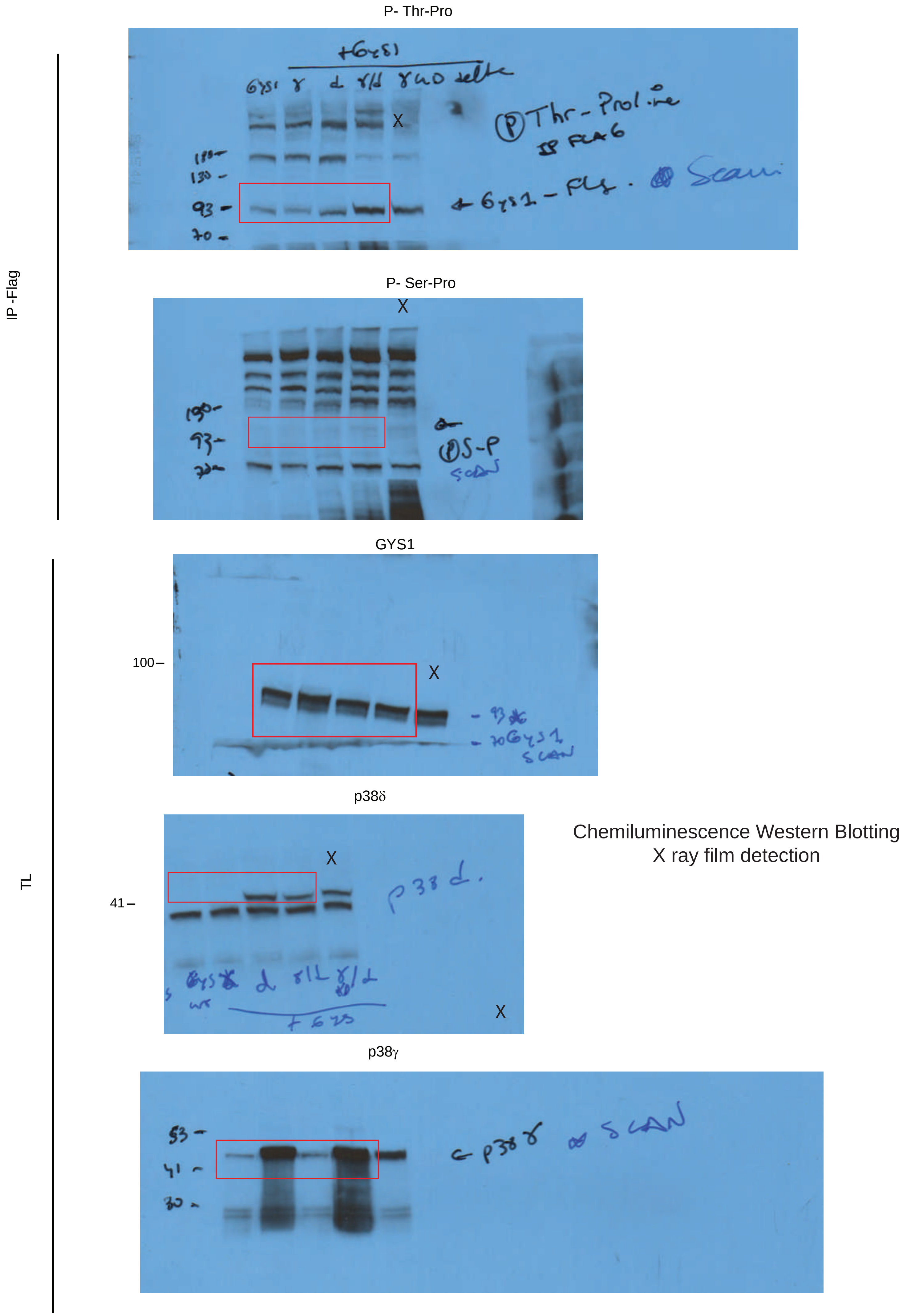
P- Thr-Pro
P- Ser-Pro
GYS1
100
p38δ
41
p38γ
IP -Flag
TL
X
Chemiluminescence Western Blotting
X ray film detection

Figure 7E

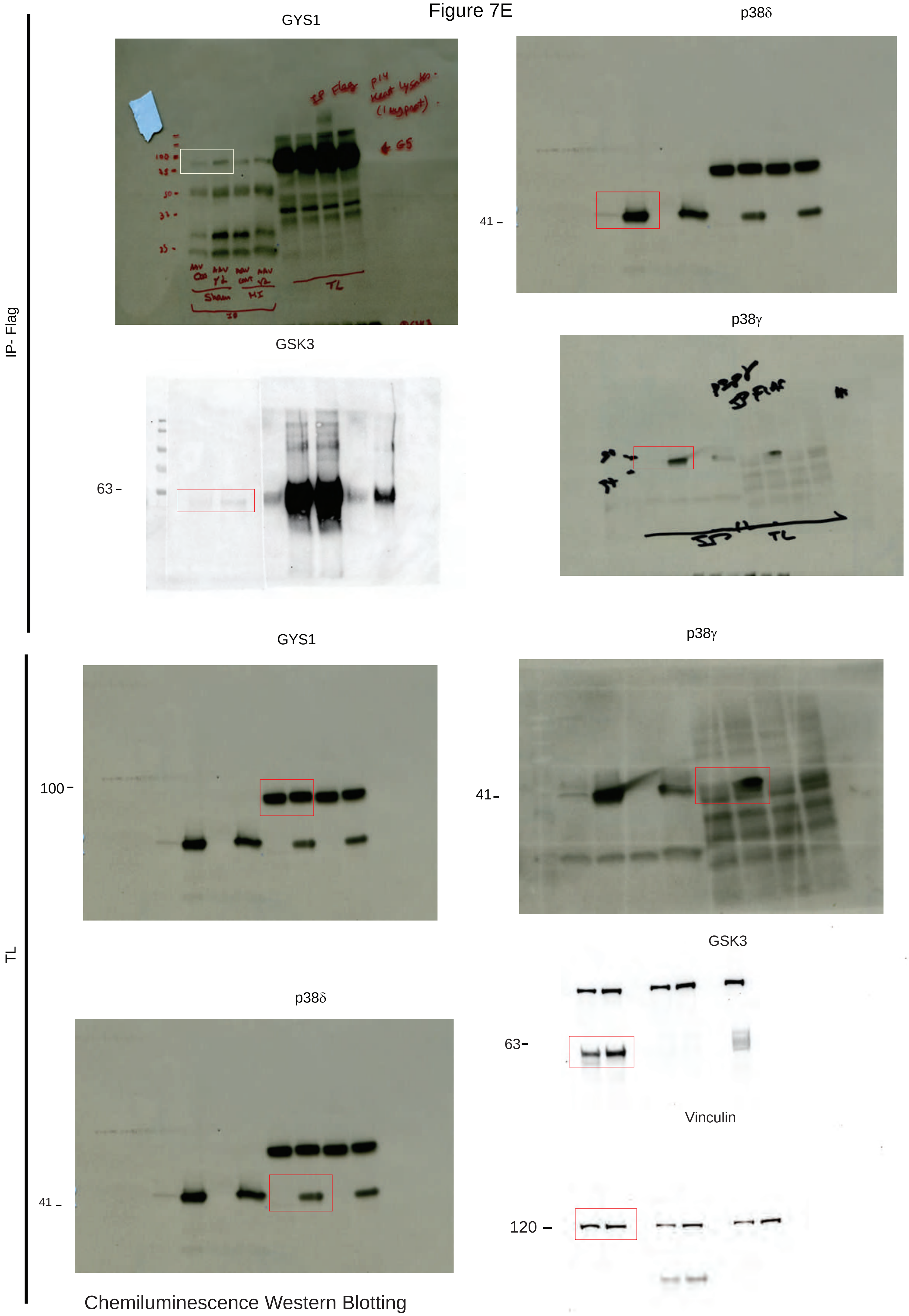

Chemiluminescence Western Blotting
X ray film and iBright 15000 detection

Figure 7F

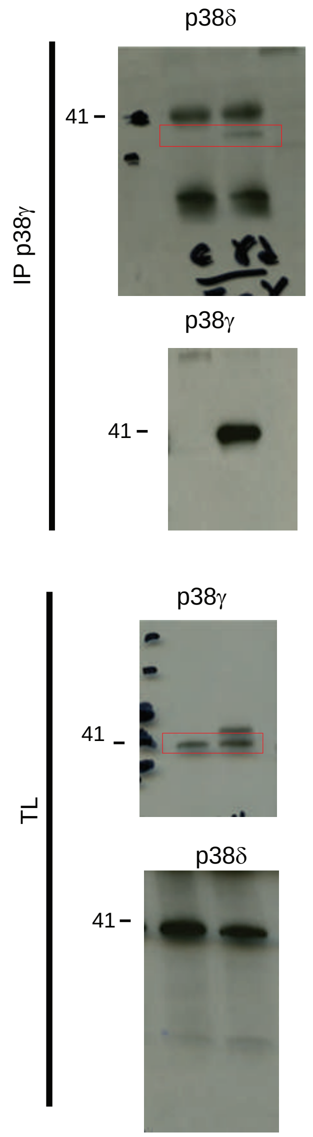

Chemiluminescence Western Blotting
X ray film detection

Figure 7G

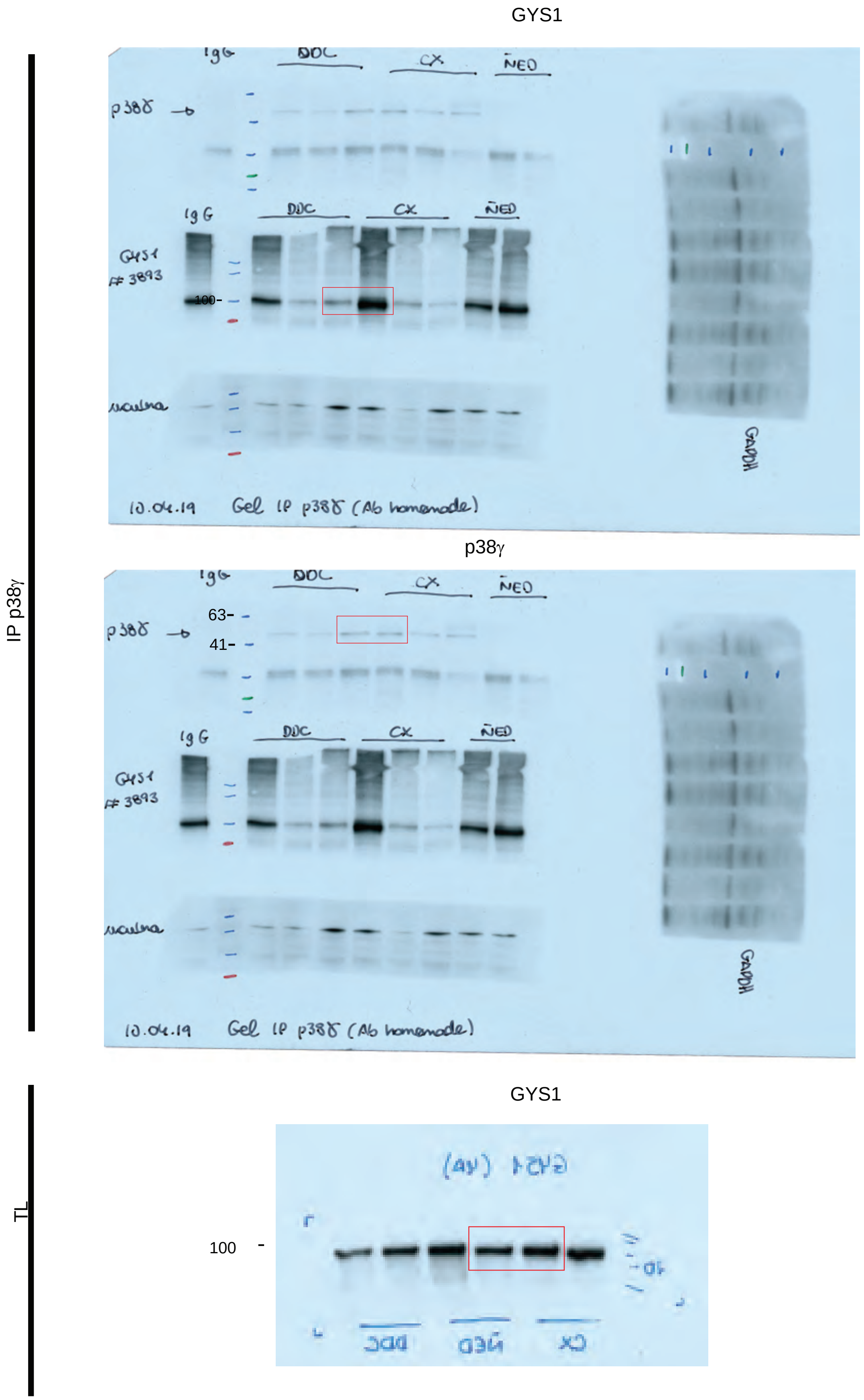

Chemiluminescence Western Blotting
X ray film detection

Figure 8A

GYS1

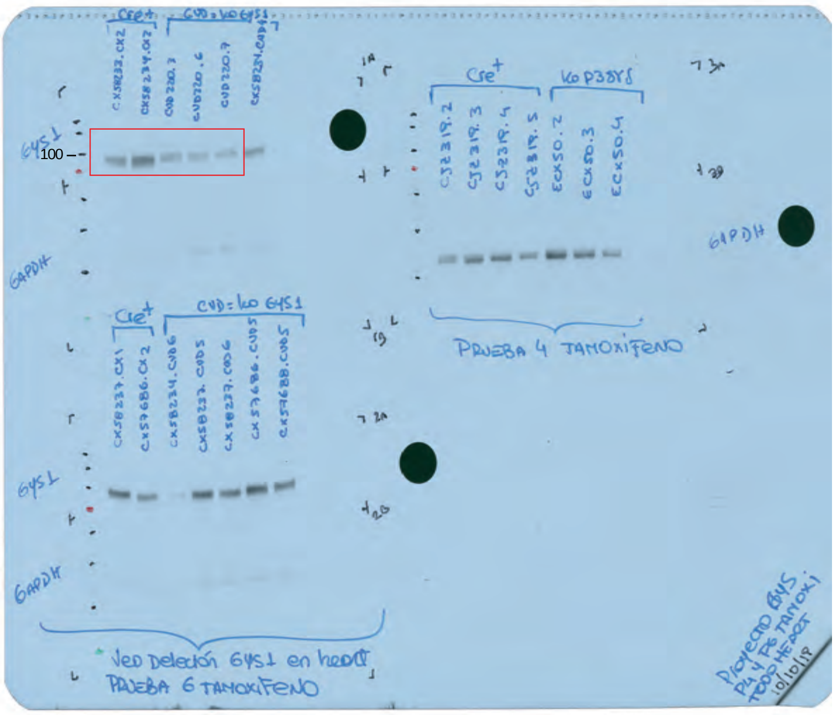

GAPDH

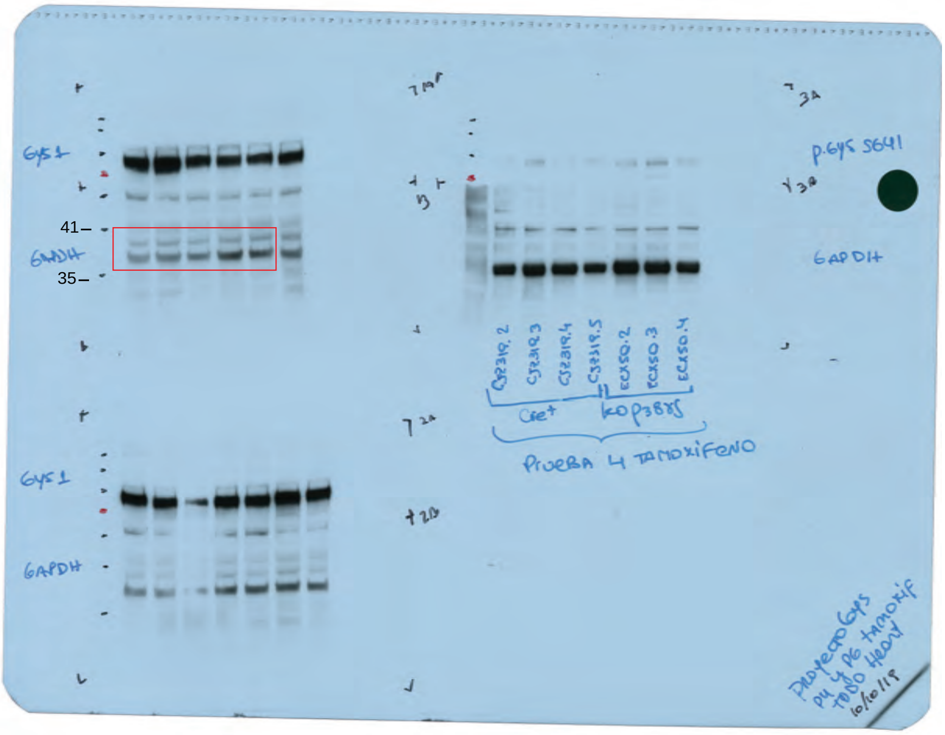

Chemiluminescence Western Blotting
X ray film detection

Supplementary Figure 4A

p-ACC

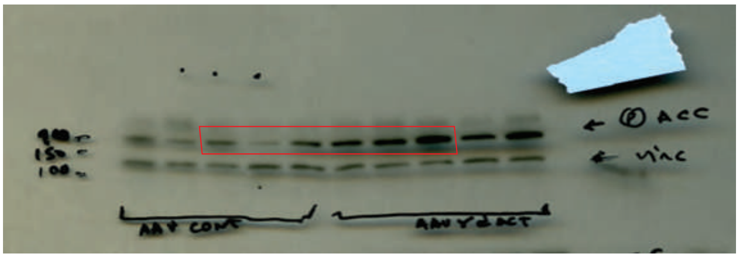

ACC

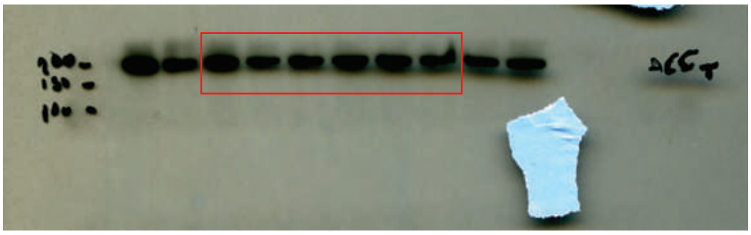

pAMPK(Thr 172)

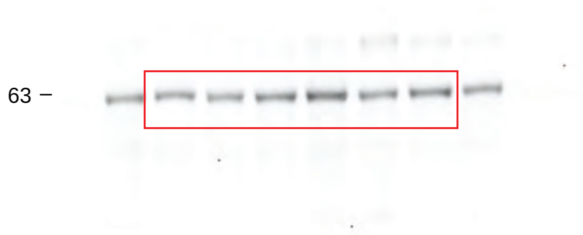

AMPK

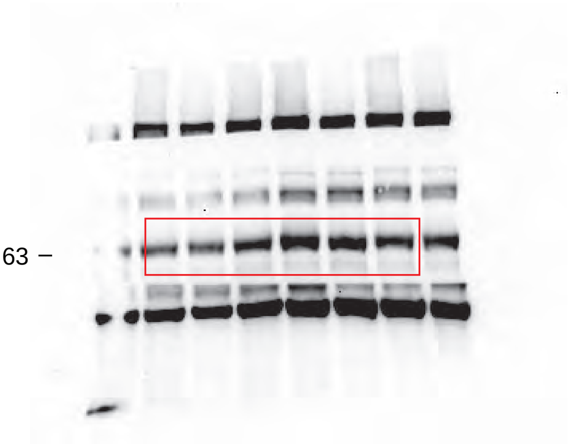

p38δ[act]

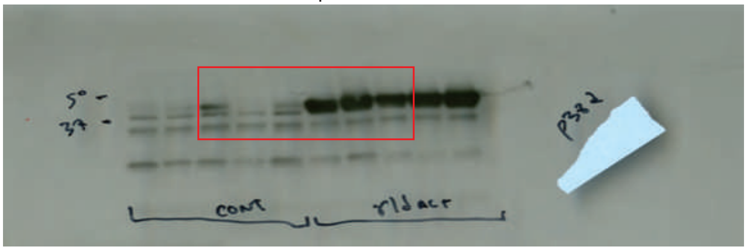

p38γ[act]

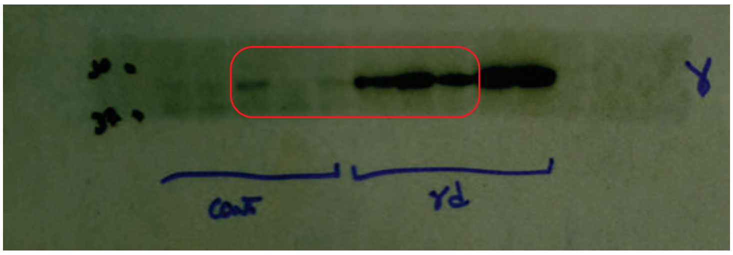

Vinculin

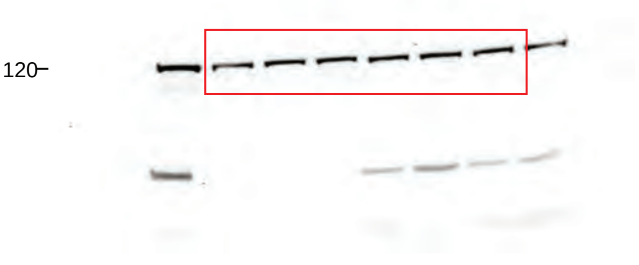

Chemiluminescence Western Blotting
X ray film and iBright 1500 detection

Supplementary Figure 4C

Mitochondria

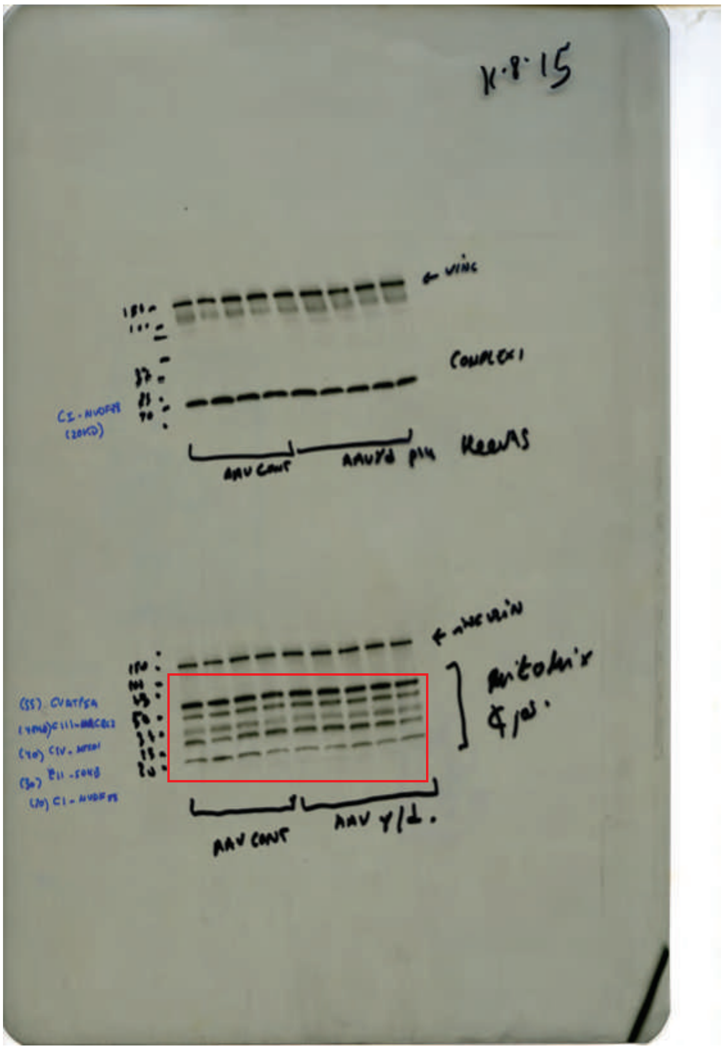

p38δ

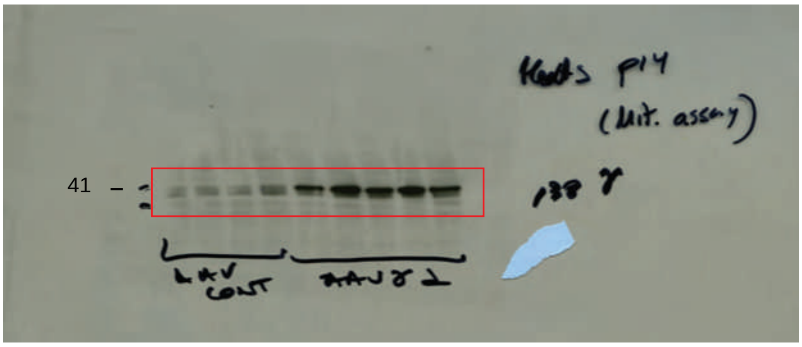

Vinculin

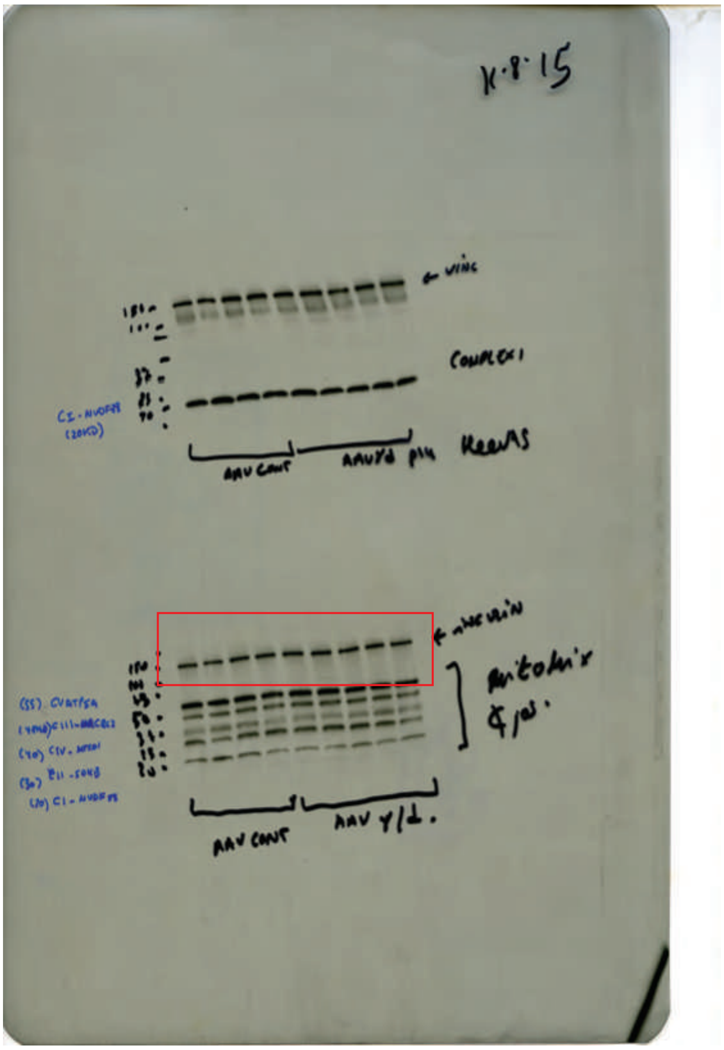

p38γ

41

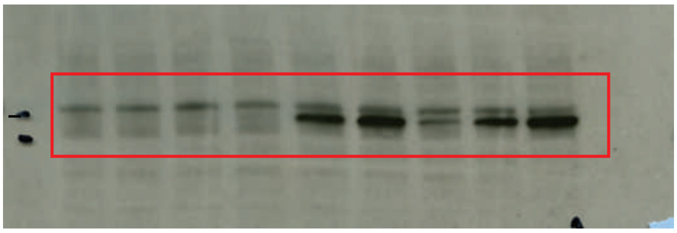

Chemiluminescence Western Blotting
X ray film detection

Supplementary Figure 5A

Liver

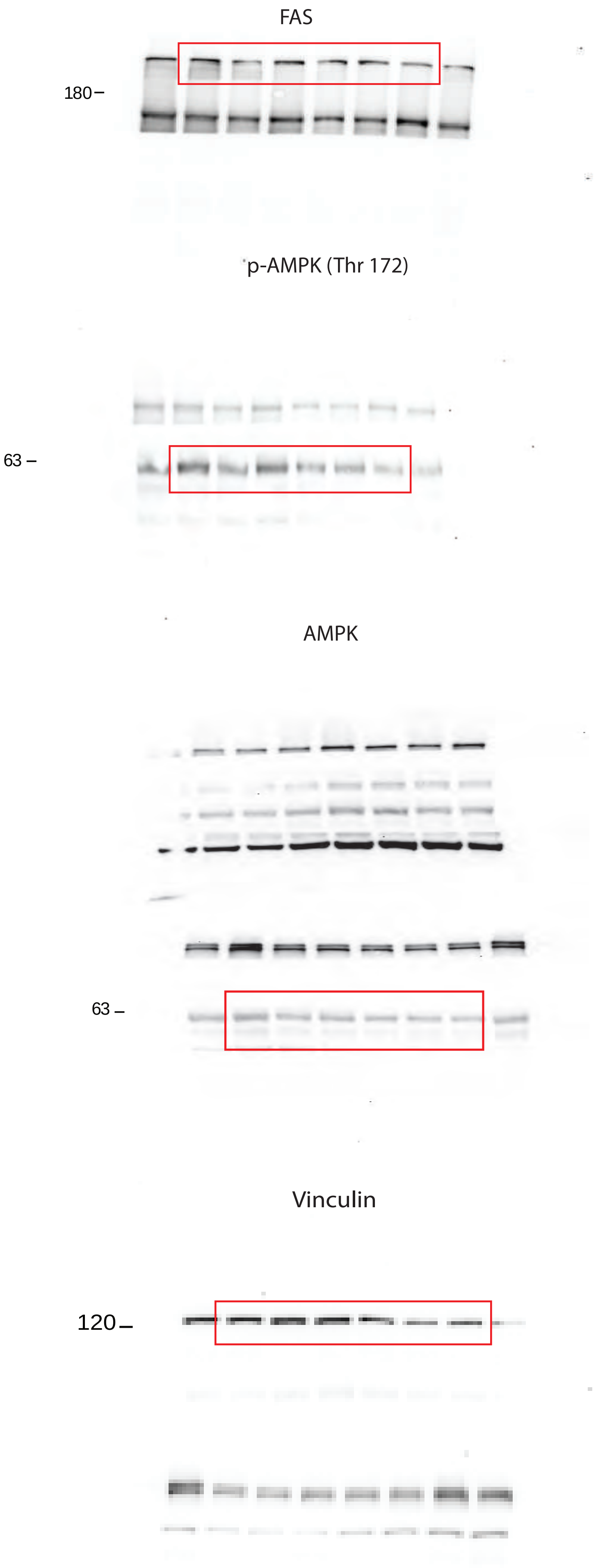

Chemiluminescence Western Blotting
IBright 1500 detection

Supplementary Figure 5B

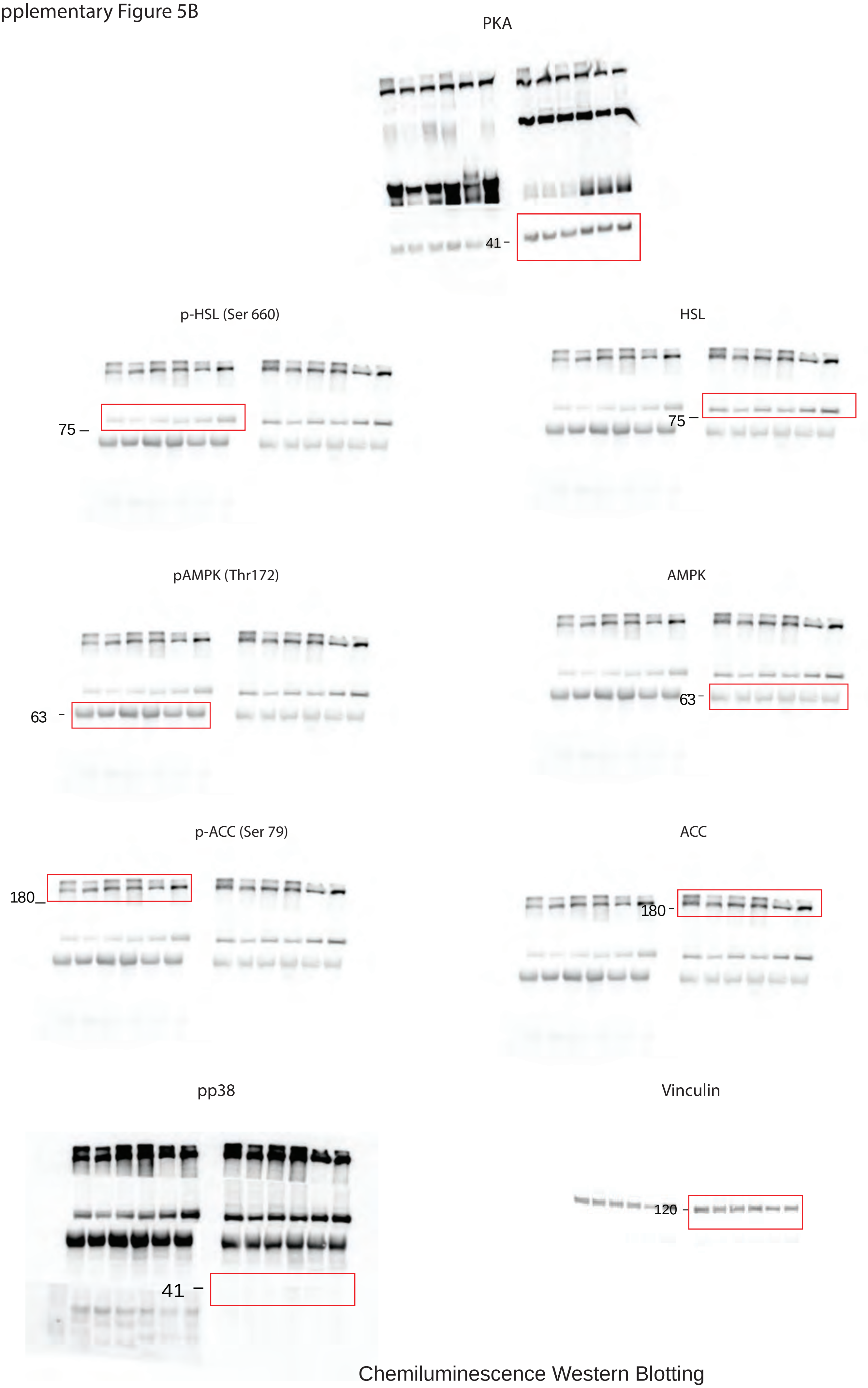

Chemiluminescence Western Blotting
IBright 1500 detection

Supplementary Figure 7A

p38γ^{act}

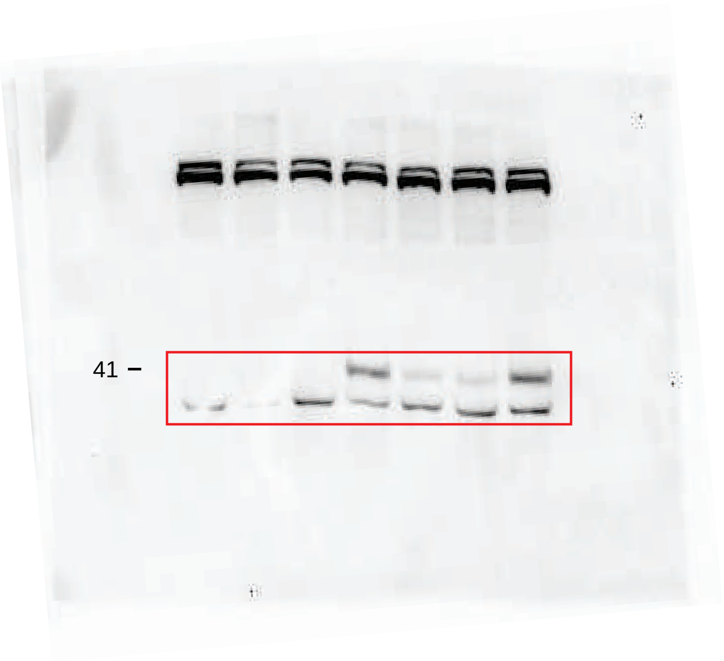

p38δ^{act}

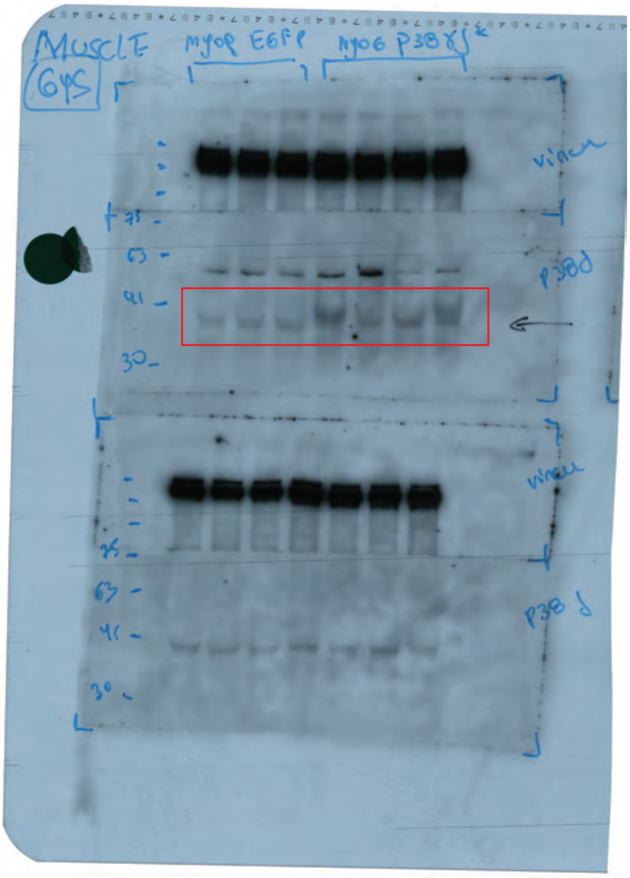

Vinculin

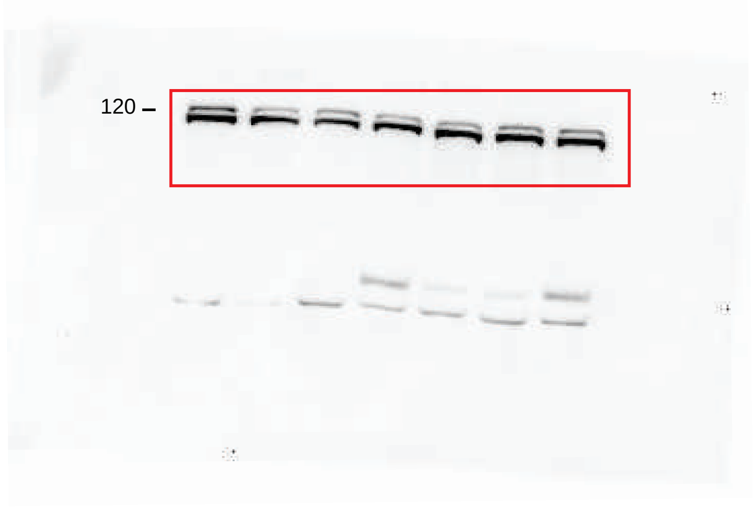

Chemiluminescence Western Blotting X ray film and iBright 1500 detection

Supplementary Figure 10

p-PKA C (Thr197)

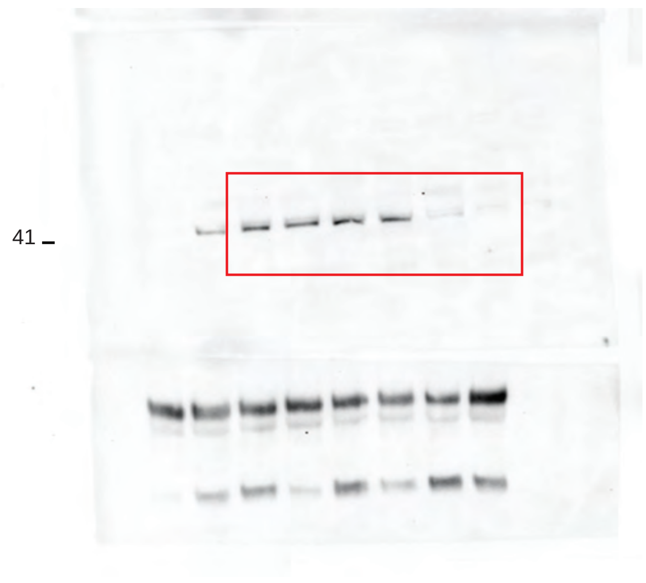

Vinculin

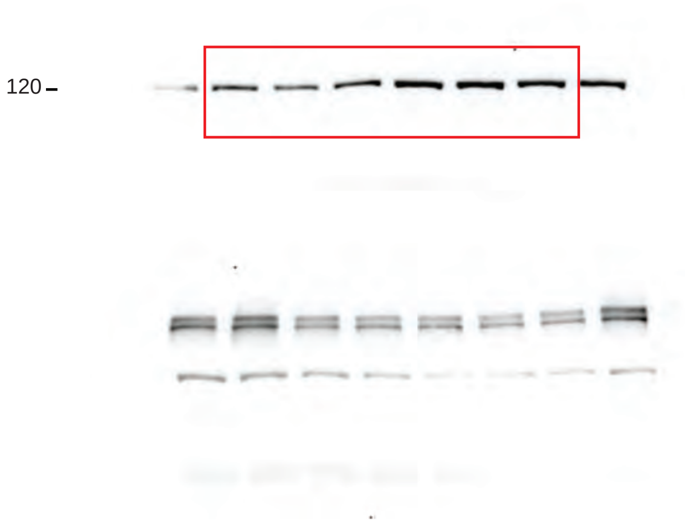

Chemiluminescence Western Blotting
iBright 1500 detection

Supplementary Figure 12A

p-GYS (S641)

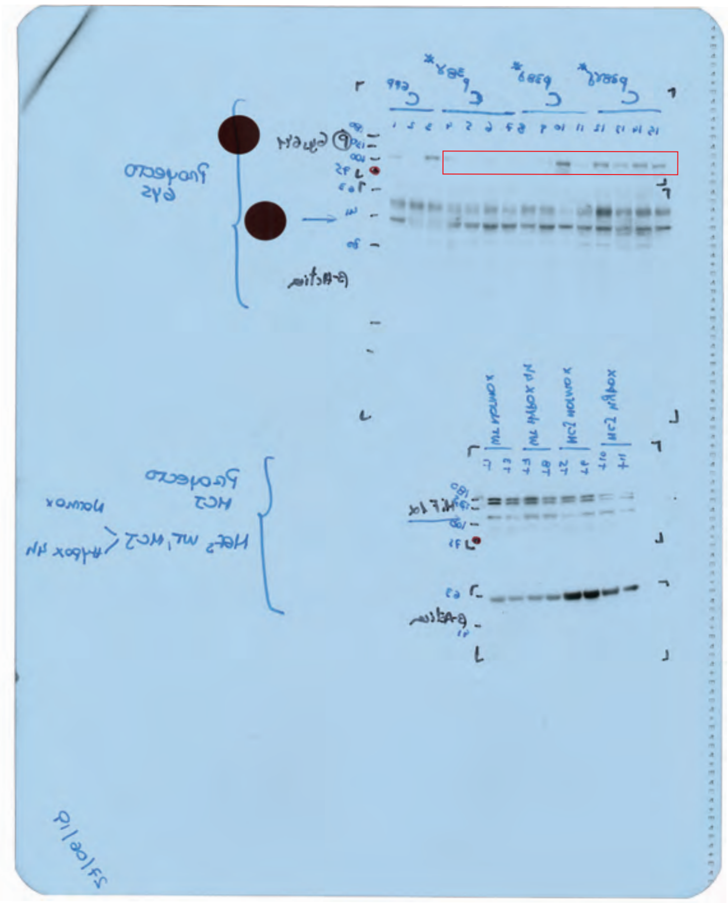

β-actin

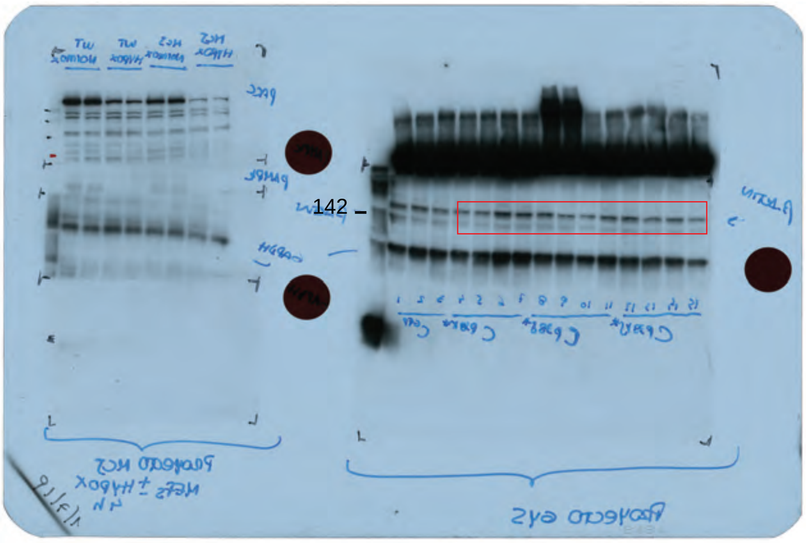

GYS1

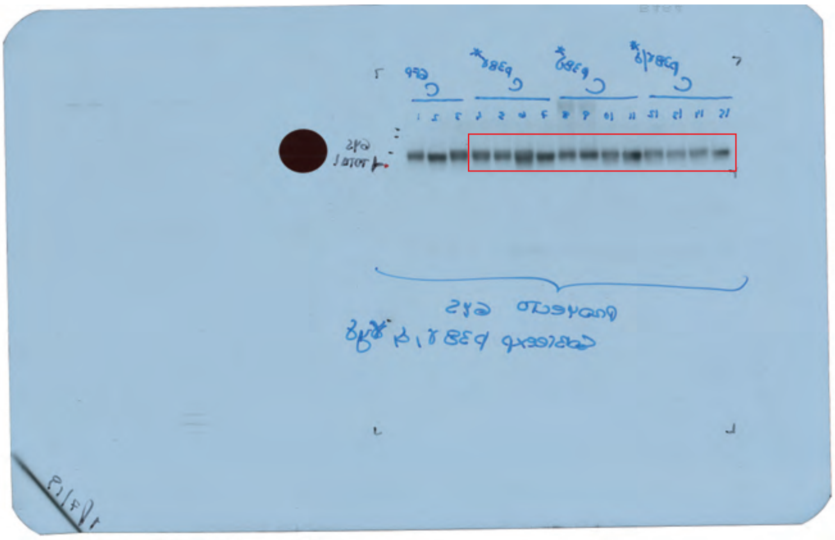

Chemiluminescence Western Blotting
X ray film detection
